# Engineering cellular communication between light-activated synthetic cells and bacteria

**DOI:** 10.1038/s41589-023-01374-7

**Published:** 2023-07-06

**Authors:** Jefferson M. Smith, Denis Hartmann, Michael J. Booth

**Affiliations:** 1 Department of Chemistry, University of Oxford, Oxford, UK; 2 Department of Chemistry, University College London, London, UK

**Keywords:** Synthetic biology, DNA, Synthetic biology

## Abstract

Gene-expressing compartments assembled from simple, modular parts, are a versatile platform for creating minimal synthetic cells with life-like functions. By incorporating gene regulatory motifs into their encapsulated DNA templates, in situ gene expression and, thereby, synthetic cell function can be controlled according to specific stimuli. In this work, cell-free protein synthesis within synthetic cells was controlled using light by encoding genes of interest on light-activated DNA templates. Light-activated DNA contained a photocleavable blockade within the T7 promoter region that tightly repressed transcription until the blocking groups were removed with ultraviolet light. In this way, synthetic cells were activated remotely, in a spatiotemporally controlled manner. By applying this strategy to the expression of an acyl homoserine lactone synthase, BjaI, quorum-sensing-based communication between synthetic cells and bacteria was controlled with light. This work provides a framework for the remote-controlled production and delivery of small molecules from nonliving matter to living matter, with applications in biology and medicine.

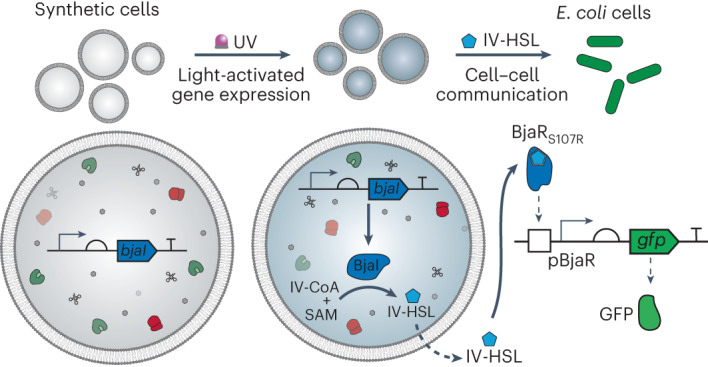

## Abstract (web summary)

Synthetic cells, modular gene-expressing compartments, have shown promising applications in biology and medicine; however, more diverse tools are required for their control and communication. Now, photocaged promoters and cell-free synthesis of an acyl homoserine lactone have been used to demonstrate light-activated communication between synthetic cells and living cells.

## Main

The process of reprogramming living systems to install new functionalities outside of their natural capabilities is a notoriously difficult endeavor. A radical approach that seeks to circumvent this process involves reducing the complexity of the system and constructing it from the bottom up using simple, modular parts. In this manner, nonliving compartments capable of life-like behaviors, denoted as synthetic cells, have been constructed to represent simplistic models of living cells. While the term synthetic cell is broad and encompasses various compartmentalized systems^1^, liposomes containing a cell-free protein synthesis (CFPS) system have become a prevalent model system, offering a neutral breadboard into which biological circuitry can be assembled^2^. Like living cells, these liposome-based synthetic cells are bound by lipid membranes, and their behaviors, including differentiation^3^ and communication^4–11^, are programmed according to DNA templates. However, their makeup is not restricted by the design rules imposed by nature, meaning they can also accommodate nonbiological components alongside their biological components to attain entirely new functionalities.

Despite the seemingly endless array of parts available to engineer synthetic cells, comparatively, few have been explored^12^. For example, of the enumerable gene regulatory networks observed in nature, in situ gene expression inside synthetic cells is often controlled at either the DNA or mRNA level using only a few well-defined small-molecule-sensitive transcription factors^6,10,11^ and translational riboswitches^4,6,13,14^, respectively. Although these tools exhibit stringent control over gene expression in vivo^15,16^, this is not recapitulated in vitro, as gene expression occurs even in the absence of the cognate ligand^4,6,13,14^. A limited subset of parts has also been deployed in most synthetic cell communication networks^12^. In this case, synthetic cells typically communicate with other synthetic cells or living cells by releasing entrapped membrane-impermeable signaling molecules using α-hemolysin^4,6,10,17,18^, or by synthesizing membrane-permeable acyl homoserine lactones (AHSLs) used in bacterial quorum sensing (QS)^5,7,8^. LuxI/R^5,11^, EsaI/R^7,10^, LasI/R^5^ and RhlI/R^8^ QS pathways have all been explored in this manner; however, they are not without their limitations. For example, both LuxI/R and EsaI/R synthesize and recognize N-(3-oxo-hexanoyl)-l-homoserine lactone (3OC6-HSL) so can’t be used in conjunction with one another, while RhlR requires high concentrations of N-butyryl-l-homoserine lactone (C4-HSL) to activate gene expression^8^. Furthermore, all the AHSL synthases explored to date either used substrate homologs^8^ or the substrates were first assembled by housekeeping enzymes present in the cell lysate^5,7^.

For synthetic cells to become a mature technology with far-reaching applications in influencing the biology of living systems or as controllable delivery devices, a more diverse tool kit is required. In this work, we explored new synthetic and biological parts to expand synthetic cell capabilities in the following two specific areas: gene expression control and chemical communication.

First, we describe an alternative means of regulating gene expression that does not rely on naturally occurring small-molecule-responsive transcription factors. Instead, gene expression inside the synthetic cells was controlled using ultraviolet (UV) light by incorporating light-sensitive 2-nitrobenzyl groups into the DNA templates. Although this has previously been achieved by installing 1-(4,5-dimethoxy-2-nitrophenyl) diazoethane groups into the backbone of plasmid-based DNA templates^19^, we specifically installed the light-sensitive groups within the T7 promoters of linear DNA templates to achieve a tighter OFF-state in the absence of UV light^20,21^. The modified promoter had the same nucleobase sequence as the standard T7 promoter, except seven thymines were replaced by amino-C6-thymine bases to enable photocleavable biotinylated (PCB) linkers to be installed into the major groove of the promoter region. Subsequent binding of monovalent streptavidin (mSA) to each biotin group created a steric blockade that impeded T7 RNA polymerase from binding to the T7 promoter. However, when exposed to UV light, the 2-nitrobenzyl group was photocleaved, and mSA was liberated. Thus, in situ gene expression inside the synthetic cells was tightly repressed in the absence of UV light but restored after exposure to UV light. Light-activated DNA (LA-DNA) templates were used to control in situ gene expression and remotely activate synthetic cells in a spatiotemporally controlled manner. Incorporating mechanisms of targeted remote control into synthetic cells, for instance, using light activation, will be an important development for their future application, enabling applications not possible with conventional small-molecule activators^12^.

Subsequently, we applied LA-DNA to control intercellular communication between synthetic and living cells. To this end, we reconstituted the BjaI/BjaR QS components from *Bradyrhizobium japonicum* to establish AHSL-producing synthetic cells (senders) and AHSL-responsive *Escherichia coli* cells (receivers). This system was chosen because the BjaI enzyme produces a branched AHSL, N-isovaleryl-l-homoserine lactone (IV-HSL), that is structurally dissimilar to most other AHSLs, and the BjaR transcription factor activates gene expression in response to picomolar concentrations of IV-HSL^22^. Furthermore, the native substrates for the BjaI AHSL synthase, S-adenosylmethionine (SAM) and isovaleryl coenzyme A (IV-CoA), are commercially available. Here *bjaI* was expressed inside the synthetic cells and catalyzed the in situ synthesis of membrane-permeable IV-HSL from membrane-impermeable IV-CoA and SAM. BjaR-expressing *E. coli* cells acted as receiver cells and coupled IV-HSL binding to the expression of a reporter gene held downstream of a BjaR-regulated promoter. During these experiments, we directed the evolution of the BjaR transcription factor toward a tighter OFF-state, greater dynamic range and improved sensitivity to IV-HSL and generated BjaR receiver cells with 135-fold activation of gene expression and EC_50_ = 0.6 nM. These developments offer new ways to control synthetic cell activities and provide foundations for their application as targeted drug-delivery devices and in studying communication in living systems.

## Results

### LA gene expression in synthetic cells

Genetically controlled synthetic cells were constructed by placing the cellular machinery necessary for CFPS inside giant unilamellar vesicles (GUVs). This was accomplished by encapsulating an inner solution containing PURExpress, 200 mM sucrose and TexasRed (TR)-dextran into egg phosphatidylcholine (Egg PC)-derived GUVs by emulsion droplet phase transfer^23^ (Supplementary Fig. 1). Gene expression within the synthetic cells was programmed according to linear DNA templates held within the inner solution, which would ultimately be modified to create the LA-DNA. These comprised a T7 promoter, strong ribosome-binding site (RBS), gene of interest (GOI) and T7 terminator (see Supplementary Methods for template sequences).

In the first instance, the linear DNA templates encoded mNeonGreen (*mNG*) to allow in situ gene expression to be monitored using fluorescence microscopy. These templates also contained a T7g10 leader sequence^24,25^ that was shown to improve protein titers in bulk CFPS reactions (Supplementary Fig. 2). When the linear *mNG*-encoding DNA templates were introduced into the synthetic cells, fluorescent GUVs were observed, confirming the successful in situ transcription and translation of functional mNG (Supplementary Fig. 3). In the absence of a DNA template, *mNG* expression was not observed, confirming the synthetic cells were programmed according to the DNA template (Supplementary Fig. 3). Fluorescence microscopy images highlighted *mNG* expression across the synthetic cell population were somewhat heterogeneous, although this was expected due to the stochasticity of solute partitioning during emulsion phase transfer^26^ and differing degrees of lipid–inner solution interactions across the polydisperse GUVs^27^. To better represent the properties of the synthetic cell population, an image processing workflow was developed that was able to measure the diameters and fluorescence intensity of individual synthetic cells (Supplementary Fig. 4a; see Methods for code). This indicated that the synthetic cells were typically 3–20 µm in diameter (mean diameter = 6.5 ± 2.3 µm; Supplementary Fig. 4b), and their fluorescence intensities spanned an order of magnitude after 8 h of incubation at 37 °C (Supplementary Fig. 4c).

**Fig. 1:**
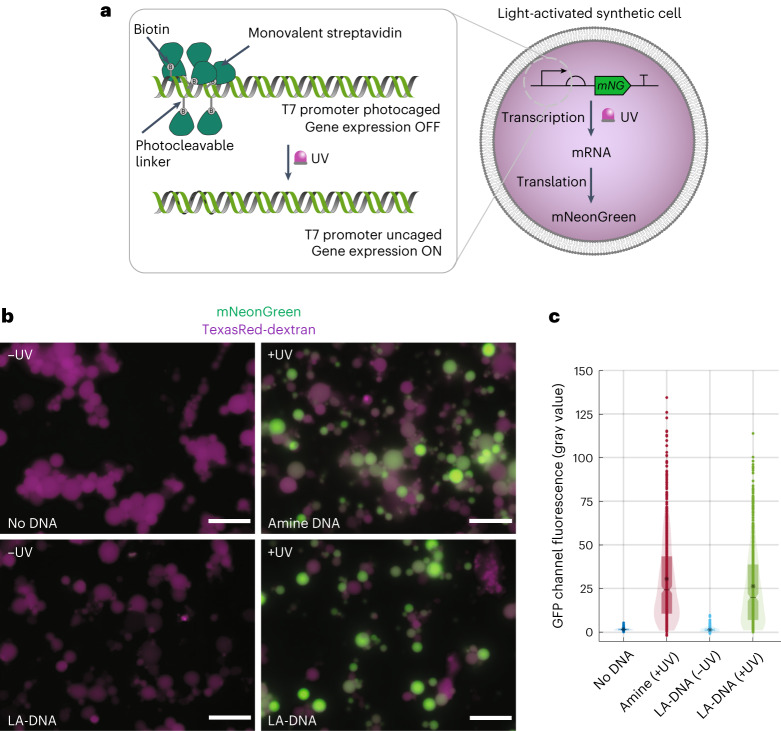
Light-activated gene expression in synthetic cells. **a**, PURExpress and LA-DNA templates were housed inside GUVs. In the absence of light, the photosensitive blockade prevented T7 RNA polymerase from binding the T7 promoter and transcribing the downstream gene of interest. After irradiation with UV light, T7 RNA polymerase could bind the uncaged T7 promoter to initiate the transcription, and subsequently translation of the protein of interest. **b**, Epifluorescence microscopy images of light-activated *mNG* expression inside synthetic cells with and without UV light exposure. Synthetic cells that were not irradiated with UV light expressed minimal mNG, and their GFP channel fluorescence intensity was consistent with synthetic cells that contained PURExpress but no DNA template. Synthetic cells exposed to UV light expressed *mNG*, as demonstrated by the increase in green channel fluorescence. mNG fluorescence intensity was consistent with synthetic cells prepared with amine-modified mNG DNA templates. Scale bar = 20 µm. Images are representative of *n* = 3 independent experiments. **c**, Quantification of *mNG* expression in the individual GUVs using a circle detection-based image analysis script. Fluorescence intensity was comparable across LA-mNG DNA (−UV) and no DNA samples, and the LA-mNG DNA (+UV) and amine mNG DNA (+UV) samples. (Median fluorescence intensity (no DNA) = 1.54 gray units (*n* = 432); median fluorescence intensity (LA-DNA (−UV)) = 1.09 gray units (*n* = 568); median fluorescence intensity (amine DNA) = 25.2 gray units (*n* = 627); Median fluorescence intensity (LA-DNA (+UV)) = 20.2 gray units (*n* = 524).) The box plot, notch and asterisk in **c** represent the interquartile range, mean and median fluorescence intensity, respectively. Source data

To control in situ gene expression with light, we installed photo-actuated T7 promoters into the linear DNA templates by using a modified oligonucleotide containing PCB linkers as a PCR primer, as described previously^20^ (Supplementary Fig. 5). LA-DNA was then formed upon the binding of mSA to the seven PCB groups within the T7 promoter of the modified DNA templates (Supplementary Fig. 6a). The resulting LA-DNA performed as expected; the photocleavable blocking groups were liberated from the DNA templates in a UV dose-dependent manner (Supplementary Fig. 6a), and the LA-T7 promoter tightly repressed *mNG* expression in bulk CFPS reactions until UV light (365 nm) was applied (Supplementary Fig. 6b). Subsequently, LA synthetic cells were assembled by encapsulating LA-DNA inside the gene-expressing GUVs (Fig. 1a). The fluorescence intensity of LA-DNA containing synthetic cells (−UV) was comparable to synthetic cells prepared without a DNA template, as gene expression was tightly repressed inside the compartments in the absence of UV light (Fig. 1b,c). In contrast, in situ *mNG* expression was recovered in >85% of synthetic cells after they were irradiated with UV light (Fig. 1b,c; fluorescence intensity threshold = mean pixel intensity of no DNA control GUVs + 3 s.d.). Like LA-mNG expression in bulk, gene expression in the LA-mNG DNA synthetic cells (+UV) reached ~80% of the intensity observed in synthetic cells containing amine *mNG* DNA (amine DNA represents the LA-DNA after 100% photocleavage; Fig. 1c). Both the proportion of *mNG*-expressing vesicles and the fluorescence intensity of those vesicles increased in line with LA-DNA concentration (Supplementary Fig. 7), and as the UV light exposure time was increased from 5 min to 10 min (Supplementary Fig. 8). However, beyond 10 min irradiation, *mNG* expression began to decrease. Thus, the LA-DNA templates retained sufficient blocking groups to prevent transcription at UV doses shorter than 10 min in duration, but gene expression was lower with prolonged UV light exposure as DNA/RNA/protein damage to the CFPS system exceeded any improvements afforded by the increased accessibility of T7 promoter sites.

**Fig. 2:**
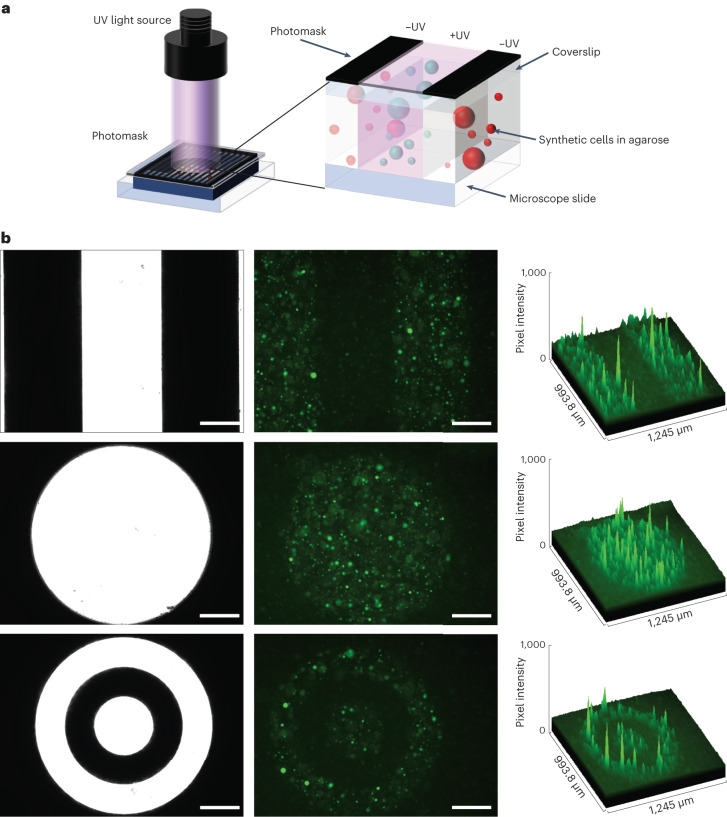
Spatially controlled activation of light-activated synthetic cells. **a**, Synthetic cells containing LA-mNG DNA were immobilized inside 1.5% agarose, and gene expression was activated with photomask-patterned UV light. Dark regions of the photomask prevented the passage of light, and the vesicles beneath did not express *mNG*. Lighter regions permitted the passage of light, uncaged the LA-DNA and stimulated gene expression. **b**, Synthetic cells only expressed mNG in the areas exposed to UV light. The *mNG* expression patterns closely matched the photomask designs. Left, photomasks; center, epifluorescence microscopy images; right, surface plots. Scale bar = 200 µm. Images are representative of *n* = 3 independent experiments.

The major advantages of using light over small molecules to activate gene expression are that light acts completely orthogonally to all small-molecule-based regulation and, unlike small molecules, can be applied in a spatially controlled manner. We exploited this to selectively activate gene expression inside light-activated synthetic cells. Patterned UV light was generated by projecting a collimated light-emitting diode through a film photomask, onto LA synthetic cells held within imaging chambers (Fig. 2a). LA-mNG DNA containing synthetic cells were held below the patterned light source inside a 1.5% ultralow gelling agarose hydrogel to prevent the GUVs from moving after light activation and maintain the integrity of the patterned structures. Synthetic cells were irradiated with the patterned UV light for 10 min, and after 6 h incubation at 37 °C, mNG fluorescence was observed only in the synthetic cells residing within the light-exposed areas (Fig. 2b). Simple photomask designs containing bulky features >200 µm in size produced the best-patterned structures, although finer details down to ~100 µm were also resolved in the more complex pattern designs (Supplementary Fig. 9). Overall, this confirmed that gene expression inside GUVs could be remotely controlled with UV light and specific populations of the LA synthetic cells could be activated via selective irradiation.

### Optimization of IV-HSL-activated gene expression in *E. coli*

**Fig. 3:**
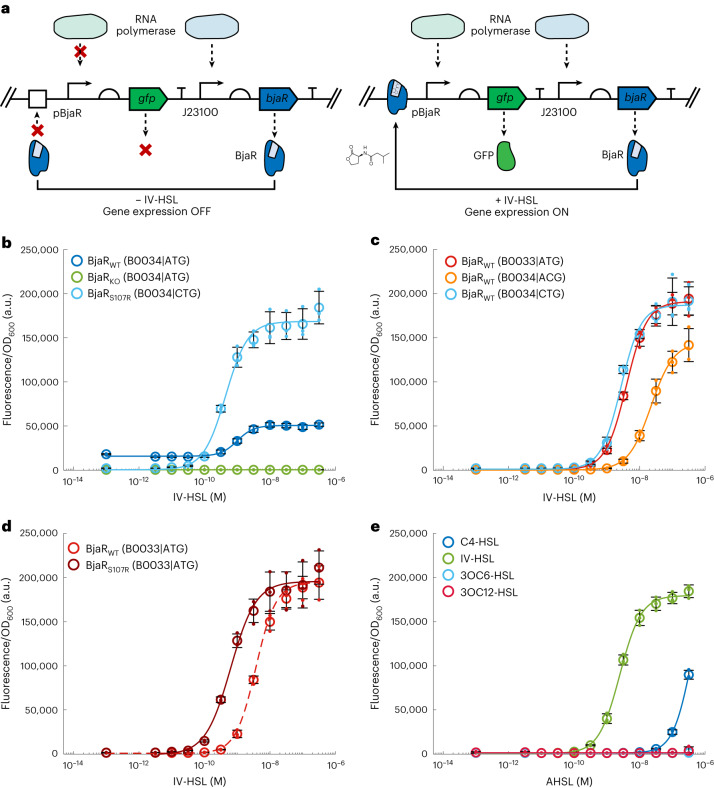
BjaR gene circuit is highly sensitive to IV-HSL. **a**, In the absence of IV-HSL, BjaR cannot recognize its consensus binding sequence and *gfp* expression is turned off. On binding to IV-HSL, BjaR binds its operator site upstream of the pBjaR promoter, recruiting RNA polymerase and activating *gfp* expression. **b**, IV-HSL increased BjaR_WT_ receiver cell GFP fluorescence 3.4-fold; however, even in the absence of IV-HSL, the receiver cells still produced considerable amounts of GFP. BjaR_KO_ receiver cells showed minimal *gfp* expression in the absence or presence of IV-HSL. Cells containing the BjaR_S107R (CTG)_ variant had a tighter OFF-state (lower baseline *gfp* expression) and an enhanced ON-state (*gfp* expression in the presence of IV-HSL), as well as marginal improvements in sensitivity to IV-HSL. **c**, The influence of the ATG → CTG variant was reproduced by placing a weak RBS sequence upstream of the *bjaR*_WT_ gene sequence. An even tighter OFF-state was observed for the ATG → ACG start codon variant, which offers a lower translation initiation rate than ATG and CTG codons. **d**, Receiver cells containing the S107R variant were >6× more sensitive to IV-HSL than BjaR_WT_. Data from separate experiments were compared on the same axis. **e**, BjaR_WT (B0033|ATG)_ receiver cells were ~100× less sensitive to C4-HSL than IV-HSL and did not express *gfp* in response to 3OC6-HSL and 3OC12-HSL. Legend brackets indicate (RBS|start codon). Plots indicate the mean values ± s.d. of *n* = 3 independent experiments. Source data

Next, we set out to establish a unidirectional communication pathway by reconstituting the BjaI and BjaR QS machinery, separately, within synthetic cells and *E. coli*, respectively. As a starting point, IV-HSL-responsive receiver cells were created by transforming *E. coli* XL10-Gold cells with a reporter plasmid, pSB1A3–*bjaR*–*gfp*^28^. This plasmid contained a *bjaR* gene under the control of a constitutively active J23100 promoter and a *gfp* gene under the control of a pBjaR promoter. The pBjaR promoter was formed by substituting the lux-box of the pLux promoter with the BjaR-binding site (Supplementary Fig. 10). Hence, GFP should only be produced when IV-HSL-bound BjaR interacts with the pBjaR promoter and recruits an RNA polymerase (Fig. 3a). Although this reporter plasmid had been characterized previously^28^, *gfp* expression was initiated with C4-HSL rather than the cognate ligand, IV-HSL. Thus, we first evaluated the dose–response relationship between synthetic IV-HSL (synthesis described in Methods) and GFP production by BjaR receiver cells. The cells were grown in M9 minimal media to an optical density at 600 nm (OD_600_) = 0.05, then transferred to a 96-well plate containing IV-HSL and incubated at 37 °C, 800 r.p.m., for 4 h. The receiver cells showed increased *gfp* expression in response to as little as 100 pM IV-HSL, and expression saturated at ~100 nM IV-HSL, confirming the gene circuit was highly sensitive to IV-HSL (EC_50_ = 1.00 ± 0.11 nM; Fig. 3b). However, *gfp* expression was also observed in the absence of IV-HSL and, consequently, the reporter circuit had a poor dynamic range (3.6-fold activation; Fig. 3b). To elucidate the root cause of the high background fluorescence, a BjaR_KO_ variant of the reporter plasmid was constructed. Knocking out BjaR completely ablated *gfp* expression, both in the absence and presence of IV-HSL (Fig. 3b). Therefore, the high OFF-state was attributed to BjaR-mediated transcription activation in the absence of IV-HSL, rather than host transcription factors recognizing the pBjaR promoter, or transcription factor independent *gfp* expression.

In pursuit of a BjaR reporter circuit with a lower baseline *gfp* expression, we used directed evolution with ON-state selection to generate more stringent BjaR variants as this approach has been successfully applied to other Lux-family transcription factors^29^ (Supplementary Fig. 11a). First, random variants were introduced throughout the entire length of the *bjaR* gene by error-prone PCR; 100 µM MnCl_2_ was added to Taq PCRs to introduce approximately two variants into the gene per reaction^29^. The resulting variant *bjaR* gene library was then cloned into a modified reporter plasmid, pSB1A3–*bjaR–gfp–kanR*, containing both *gfp* and the kanamycin resistance gene (*kanR*) downstream of the pBjaR promoter, and transformed into *E. coli* cells (Supplementary Fig. 11b). To remove nonfunctional BjaR variants that had acquired deleterious mutations from the library but retain cells that possessed a functional BjaR variant, the cells underwent ON-selection in the presence of kanamycin. This selection process recovered both the desirable BjaR variants—those carrying variants that afforded a tight OFF-state without compromising *kanR* expression in response to low concentrations of IV-HSL, as well as undesirable BjaR variants— those which survived via leaky *kanR* expression. Hence, to enrich the library with these highly stringent BjaR variants, the cells were washed and grown in M9 minimal media containing ampicillin but no IV-HSL. During this outgrowth step, cells that contained stringent BjaR variants produced less KanR and GFP and, therefore, experienced lower cellular burden and grew faster than their leakier counterparts. Plasmids from all cells present after outgrowth were then used as the DNA template in the next round of evolution.

After four rounds of random mutagenesis, ON-selection and outgrowth, the OFF- and ON-states of 45 BjaR variants were screened by measuring their GFP fluorescence in the absence and presence of 1 nM IV-HSL. Forty of these variants demonstrated an improved dynamic range over BjaR_WT_, with 14 showing >25-fold activation of *gfp* expression (Supplementary Fig. 11c). DNA sequencing of the top three performing variants, colony_11, colony_28 and colony_36, revealed that they all contained the same mutations within the translation initiation codon (ATG → CTG) and Ser107 (AGC → CGC, Ser → Arg). Interestingly, another stringent BjaR variant identified in preliminary work also carried a variant in the start codon (ATG → ACG), highlighting the importance of mutating the initiation codon in generating a tight OFF-state. To understand the true impact of these mutations on receiver cell performance, the *bjaR*_S107R (CTG)_ and *bjaR*_WT (ACG)_ genes were cloned back into the reporter plasmid backbone without *kanR*. Under this context, BjaR_S107R (CTG)_ receivers demonstrated a ~88-fold activation of *gfp* expression granted by a 7.2-fold decrease in the OFF-state and 3.6-fold increase in the ON-state, relative to BjaR_WT_, and were also more sensitive to IV-HSL (EC_50_ BjaR_S107R (CTG)_ = 0.44 ± 0.04 nM; Fig. 3b). Meanwhile, the BjaR_WT (ACG)_ receivers demonstrated a ~309-fold activation of *gfp* expression granted by a 31.6-fold decrease in the OFF-state, and a 2.7-fold increase in the ON-state, but were far less sensitive to IV-HSL (EC_50_ BjaR_WT (ACG)_ = 22.3 ± 2.59 nM).

Given different mutations within Met1, both drastically improved the OFF-state of the receiver cells, the changes in the ATG start codon likely perturbed the rate of translation initiation but still lead to the incorporation of an N-formylmethionine^30^. Using RBSs with decreasing strengths to decouple the translation initiation rate from the identity of the initiation codon (Supplementary Fig. 12a), we demonstrated that simply reducing the pool of BjaR available within the cells created receivers that mirrored the dose–response behavior of those that initiate translation via a CTG start codon (Fig. 3c and Supplementary Fig. 12b). In fact, the dose–response curves for BjaR receivers carrying the weakest RBS sequence tested (BjaR_WT (B0033|ATG)_; whereby (RBS|initiation codon)) and the CTG start codon alone (BjaR_WT (B0034|CTG)_) had similar hill coefficients (1.38 versus 1.41), EC_50_ (BjaR_WT (B0033|ATG)_ = 3.93 ± 0.38 nM versus BjaR_WT (B0034|CTG)_ = 2.68 ± 0.48 nM) and fluorescence maxima (Fig. 3c). These findings are consistent with the predicted translation initiation rates of the respective sequences—translation from the BBa_B0033 RBS provides 1% of protein yield compared to the BBa_B0034 RBS, just as an CTG start codon provides 1% of the protein yield compared to an ATG start codon^30,31^. This also offers support as to why the ON-state *gfp* expression level increased in variants with a tighter OFF-state; more cellular resources were available for GFP synthesis when comparatively fewer were allocated to BjaR synthesis. To elucidate the impact of the S107R variant on gene circuit performance, we introduced the S107R variant alone back into the *bjaR*_WT_ gene, in the context of the BBa_B0033 RBS background. The BjaR_WT (B0033|ATG)_ and BjaR_S107R (B0033|ATG)_ receiver strains had identical dose–response behaviors, except the BjaR_S107R (B0033|ATG)_ variant was >6× more sensitive to IV-HSL than the BjaR_WT (B0033|ATG)_ (EC_50_ BjaR_S107R (B0033|ATG)_ = 0.63 ± 0.10 nM; Fig. 3d). Overall, cells transformed with the BjaR_S107R (B0033|ATG)_ reporter plasmid granted the best combination of performance (135-fold activation range) and sensitivity and exhibited a 1.15-fold increase in the ON-state, plus a 1.33-fold decrease in the OFF-state compared to the BjaR_S107R (B0034|CTG)_ variants obtained by directed evolution (Supplementary Fig. 12c).

In addition to quantifying how these BjaR receiver cells responded to their branched, cognate ligand, we also sought to determine whether the BjaR receiver cells could be activated by structurally dissimilar AHSLs involved in the LuxI/R, EsaI/R, LasI/R and RhlI/R QS systems previously used in synthetic cell communication^5,7,8^. To this end, we measured the fluorescence of BjaR receiver cells grown in the presence of their activators, 3OC6-HSL, 3OC12-HSL and C4-HSL, respectively (Supplementary Fig. 13a). BjaR_WT (B0033|ATG)_ receiver cells were partially activated by the maximal C4-HSL concentration tested (0.36 µM); however, ~126× more C4-HSL was required to achieve the same fluorescence output compared to IV-HSL (Fig. 3e). The same trend was observed for BjaR_S107R (B0033|ATG)_ receiver cells, except the IV-HSL and C4-HSL dose–response curves were shifted toward lower AHSL concentrations proportionally with one another (Supplementary Fig. 13b,c). This suggests that the S107R variant does not influence the substrate specificity of BjaR and the interaction between the AHSL- and BjaR-binding site is strengthened via a motif present in both AHSLs. For both of the receiver cells tested, minimal GFP was produced by cells grown in 3OC6-HSL or 3OC12-HSL (Fig. 3e and Supplementary Fig. 13b), confirming the BjaR ligand-binding pocket preferentially accommodates smaller acyl chains devoid of C-3 carbonyl groups. Hence, BjaR receiver cells demonstrated substrate-level orthogonality with the LuxI/R, EsaI/R and LasI/R QS systems used previously in synthetic cell communication.

### LA communication between synthetic cells and living cells

**Fig. 4:**
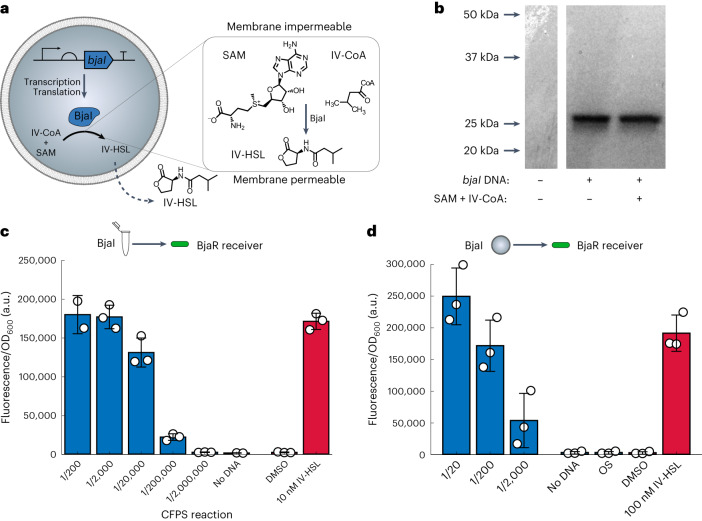
IV-HSL biosynthesis by *bjaI*-expressing synthetic cells. **a**, IV-HSL biosynthesis by synthetic cells catalyzed by the in situ expression of the *bjaI* AHSL synthase. Membrane-impermeable substrates, IV-CoA and SAM, are converted into the membrane-permeable signaling molecule IV-HSL. IV-HSL subsequently diffuses across the lipid bilayer into the external solution. **b**, Full-length BjaI was obtained in bulk PURExpress reactions in the absence or presence of IV-CoA and SAM. **c**,**d**, BjaI produced in bulk CFPS reactions (**c**) and inside GUVs (**d**) was enzymatically active and synthesized IV-HSL from SAM and IV-CoA. In total, 17.9 ± 2.6 µM IV-HSL was produced by bulk BjaI CFPS reactions. In total, 458.5 ± 119.6 nM IV-HSL was produced in synthetic cells. IV-HSL produced in bulk or by synthetic cells was quantified using BjaR_S107R (B0034|CTG)_ and BjaR_S107R (B0033|ATG)_ receiver cells, respectively. The *x* axis labels represent the dilution factor of the CFPS reaction in the cultured cells, that is, 1/200 indicates that 1 µl of CFPS was added to 200 µl of cells. Columns and bars indicate the mean ± s.d. of *n* = 3 independent experiments, shown as open circles. Source data

With an IV-HSL-sensitive BjaR receiver cell in place, we turned our attention to creating IV-HSL-producing synthetic cells. This was accomplished by producing the BjaI AHSL synthase within synthetic cells containing its native substrates, IV-CoA and SAM. IV-CoA (851.65 g mol^−1^) and SAM (399.44 g mol^−1^) are bulky, charged, membrane-impermeable molecules (log *P*_IV-CoA_ = −4.3; log *P*_SAM_ = −2.8), while IV-HSL produced by BjaI is smaller (185.22 g mol^−1^) and more hydrophobic (log *P*_IV-HSL_ = 0.9) so passes freely across lipid bilayers. Hence, by expressing *bjaI* in GUVs containing SAM and IV-CoA, we anticipated IV-HSL would be synthesized in situ and then diffuse across the lipid bilayer, as is the case with other AHSLs generated in synthetic cells^5,7,8^ (Fig. 4a).

We first validated *bjaI* expression using bulk CFPS reactions containing (^35^S)-methionine to visualize the newly synthesized protein. The full-length BjaI protein (25 kDa) was successfully translated, and its expression was not influenced by the presence of SAM and IV-CoA (Fig. 4b). To establish whether the BjaI was enzymatically active, CFPS reactions containing 80 µM IV-CoA and 300 µM SAM were incubated at 37 °C for 5 h, and then serially diluted and incubated with BjaR_S107R (B0034|CTG)_ receiver cells. Indeed, IV-HSL was successfully synthesized within the CFPS reactions as the reaction mixture containing the complete set of components (*bjaI* DNA + SAM + IV-CoA) fully activated *gfp* expression in the receivers, while no fluorescence output was observed from CFPS reactions containing SAM and IV-CoA, but without *bjaI* DNA template (Fig. 4c,d). By comparing the fluorescence of the receivers in response to the biosynthetic IV-HSL with the dose–response curves obtained using the synthetic IV-HSL (Fig. 3c), we estimated that the CFPS reaction had a concentration 17.9 ± 2.6 µM IV-HSL; thus, 17.9 ± 2.6 pmol µl^−1^ IV-HSL was generated in the bulk CFPS reactions (Fig. 4c). Similarly, on transferring this system inside GUVs, BjaI-producing synthetic cells also synthesized sufficient IV-HSL to fully activate *gfp* expression in BjaR_S107R (B0033|ATG)_ receiver cells. IV-HSL was present at 458.5 ± 119.6 nM in the 25 µl synthetic cells samples; thus, 2.28 ± 0.60 pmol µl^−1^ IV-HSL was generated by the GUVs. The loss in specific activity between CFPS performed in bulk compared to inside GUVs was attributed to the loss of inner solution during emulsion phase transfer and when the GUVs ruptured, less efficient CFPS in GUVs compared to in bulk, and absence of gene expression in some GUVs.

**Fig. 5:**
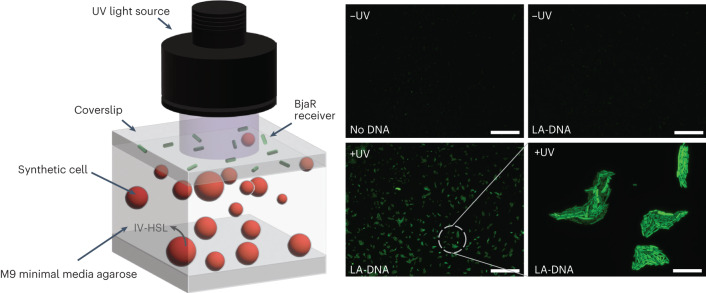
Light-activated communication between synthetic cells and living cells. Light-activated sender synthetic cells were immobilized inside agarose pads containing M9 minimal media. BjaR_S107R (B0034|CTG)_ receiver cells were subsequently applied on top of the synthetic cell-laden agarose pads. When irradiated with UV light, BjaI and subsequently IV-HSL were produced inside the synthetic cells. IV-HSL diffused across the lipid membrane, into the agarose, and activated *gfp* expression in the receiver cells held above. GFP fluorescence was not observed in receivers grown on LA-SC agarose pads that were not exposed to UV light, or on agarose pads containing SCs without *bjaI* DNA templates. Scale bar = 200 µm. Scale bar (bottom right) = 20 µm. Images are representative of *n* = 2 independent experiments.

Until now, synthetic cells had been prepared using 1× PURExpress and 200 mM sucrose and had an osmolarity of ~1,200 mOsm—far exceeding that of M9 minimal media (~300 mOsm). Before we could coculture the synthetic cells with the receiver cells, we first reduced the osmolarity of the inner solution to minimize the influx of water, GUV swelling and membrane rupture that would ensue when transferring them into M9 minimal media. PURExpress is reported to tolerate slight deviation from its working concentration, and so we tested IV-HSL production by BjaI obtained using PURExpress operating below the 1× working concentration. This confirmed that IV-HSL yields were only slightly perturbed at 0.4× the working PURExpress concentration in bulk (Supplementary Fig. 14a), and IV-HSL was still synthesized by GUVs containing 0.5× PURExpress (Supplementary Fig. 14b). GUVs prepared with an inner solution comprising 0.5× PURExpress, without murine RNase inhibitor, and 50 mM sucrose produced 32.5 ± 13.6 nM IV-HSL (0.16 ± 0.068 pmol µl^−1^ inner solution). These synthetic cells were then interfaced with living cells by growing BjaR receiver cells on BjaI DNA synthetic cell-laden agarose pads that were prepared with M9 minimal media. In this way, IV-HSL synthesized inside the GUVs by BjaI would passively diffuse across the lipid bilayer and into the agarose pad to activate *gfp* expression in the receiver cells. Fluorescence microscopy of TR-dextran labeled SCs containing 0.5× inner solution indicated that the reduced osmolarity SCs were stable in the agarose pads for at least 16 h at 37 °C (Supplementary Fig. 15a) and that BjaR_S107R (B0034|CTG)_ receiver cells were equally fluorescent when grown on M9 agarose pads containing 10 nM IV-HSL or BjaI synthetic cell-laden agarose pads. Hence, IV-HSL was successfully synthesized and released into the agarose pad at concentrations sufficient to fully activate *gfp* expression (Supplementary Fig. 15b). Using this platform, we subsequently demonstrated remotely controlled synthetic cell-to-living cell communication by encoding the *bjaI* gene on LA-DNA. The fluorescence of BjaR receiver cells grown on LA-DNA synthetic cells containing agarose pads was equivalent to that of receiver cells grown on agarose-containing GUVs without the *bjaI* template, indicating that minimal IV-HSL was produced by the synthetic cells. Meanwhile, *gfp* expression was successfully activated after irradiation with UV light, although the cells were less fluorescent than with 10 nM IV-HSL or amine BjaI DNA synthetic cells, because IV-HSL yields were below the concentration threshold for full reporter gene expression (Fig. 5).

## Discussion

Synthetic cells are a versatile technology with the potential to serve as smart delivery devices or as chassis for creating life from scratch. Despite the development of new tools and improvements in synthetic cell assembly methods, the biological parts used to regulate their activity have limited their reach to highly controlled laboratory environments^12^. In the field’s preliminary work, well-established arabinose and IPTG-inducible transcription factors and theophylline-responsive riboswitches were used to control in situ gene expression^5,6^. Still, each performed poorly in vitro and represented a leaky, insensitive route of transcription/translation control. Later, the transition to AHSL-sensitive transcription factors afforded synthetic cells the ability to sense and produce more biologically useful QS molecules, which are central to coordinating collective bacterial behaviors. Although this marked considerable progress toward integrating synthetic cells with living cells, the most frequently adopted QS systems used to date, LuxR/LuxI and EsaR/EsaI, recognize and synthesize the same AHSL (3OC6-HSL), limiting the variety of synthetic cell activators that work orthogonally^5,7,10,11^.

In this work, we diverged from using naturally derived parts to control gene expression, instead utilizing chemically modified LA-DNA templates to tightly and precisely control the location of synthetic cell activation with UV light. This LA-DNA approach was subsequently implemented to regulate communication with *E. coli* cells using the BjaI/BjaR QS system, adding this unique branched AHSL into the synthetic cell communication toolbox. We believe this system is ideally suited to synthetic cell communication. It couples an acyl-CoA-dependent synthase, BjaI, which efficiently synthesizes IV-HSL from its commercially available substrates, IV-CoA and SAM, with a highly sensitive IV-HSL-dependent transcription factor, BjaR, that activates gene expression at picomolar concentrations of IV-HSL. This strongly contrasts the properties of the RhlI/RhlR QS used previously in synthetic cells communication, which utilizes a CoA substrate less readily than its native ACP-derived substrate^32^, and requires >100 µM C4-HSL to fully activate expression from pRhlI promoters^33^. To access the full capabilities of BjaR-regulated gene expression, we used directed evolution to identify variants that improved the dynamic range of the receiver cells. We found that mutations at the initiation methionine drastically decreased the OFF-state and improved the ON-state, while a mutation at Serine107 (S107R) rendered the receiver cells more sensitive to IV-HSL. By using a weak RBS sequence in place of the ATG → CTG methionine variant, the same improvements in OFF- and ON-states were achieved suggesting a decrease in translation initiation of BjaR was responsible. This simple change might also be useful to modulate the dose–response behavior of related QS reporter systems^28^. While this work was underway, another example of IV-HSL responsive gene expression was described that used a separate BjaR reporter plasmid system containing a mutated pBjaR promoter^34^. It may be the case that this too reduced the potential of BjaR to activate the pBjaR promoter by reducing the rate of transcription initiation. By encoding the *bjaI* gene on the LA-DNA templates, IV-HSL biosynthesis was activated by exposing the synthetic cells to UV light. IV-HSL was membrane permeable and readily diffused across the lipid bilayer. We hoped to spatially pattern communication with living cells; however, this was not possible with the current setup as *bjaI* expression occurred on a much longer timescale than IV-HSL diffusion, and the membrane-permeable nature of IV-HSL meant it was not retained within the cells.

In the current iteration of LA-DNA presented here, UV light was necessary to stimulate photocleavage of the 2-nitrobenzyl groups contained within the LA-T7 promoters; however, the modular nature of the LA-DNA templates makes them fully customizable. By installing longer wavelength photocages within the photocleavable biotin group, gene expression might be regulated according to more biocompatible and tissue-penetrating visible or near-infrared light. In the future, LA synthetic cells might be used to study the mechanisms of cellular communication or initiate communication pathways between living cells in a spatiotemporal manner. For instance, LA synthetic cells might be a useful tool for studying communication in biofilms by producing AHSLs or quorum-quenching molecules originating at precise locations. Additionally, near-infrared-activated synthetic cells might be used as drug-delivery devices that produce and secrete a therapeutic small molecule or protein at a target site.

## Methods

### Materials

All consumables, solvents and reagents were purchased from Sigma/Merck unless stated otherwise. NHS–PCB–Biotin was purchased from Ambergen and stored in dry dimethylformamide at −80 °C. DreamTaq DNA polymerase master mix, TR-dextran (10 kDa) and 25 µl gene frames were purchased from Thermo Fisher Scientific. PURExpress and all other enzymes were purchased from New England Biolabs. *E. coli* XL10-Gold cells were purchased from Agilent. Egg PC was purchased from Avanti Polar Lipids. The 1 mm diamond drill bit was purchased from Eternal Tools. The M365L2-C5 UV lamp was purchased from Thorlabs. Standard DNA oligonucleotides were synthesized by integrated DNA technologies. Photomasks were designed using AutoCAD and purchased from JD Photodata. Amine-modified DNA oligonucleotides were synthesized by ATD-Bio. pMAT–*mNG* was synthesized by GeneArt. pSB1A3–*bjaR–gfp* was a gift from K. Haynes (Arizona State University). pET24a was a gift from B. Davis (University of Oxford). Monovalent streptavidin was a gift from M. Howarth (University of Oxford). All DNA sequences can be found in Supplementary Data.

### Plasmids

*mNG* and *BjaI* genes were introduced into the PURExpress control template (now referred to as pPURE) in place of *DHFR* to create pPURE–*mNG* and pPURE–*BjaI*. pPURE–*mVenus* was prepared in the previous work^20^. pPURE–T7g10–*gp10(1-9)::mNG* and pPURE–T7g10–*gp10(1-9)::mVenus* were prepared by replacing the 5′ UTR sequence of the pPURE plasmids with the T7g10 leader sequence^25^ and introducing a ten amino acid leader at the N-terminus of the respective genes. pPURE-*gp10(1-9)::mNG* was prepared by introducing only the ten amino acid leader sequence 5′ to the *mNG* gene. pSB1A3–*bjaR*_KO_–*gfp* was prepared by truncating the *bjaR* gene (*bjaR*_1–12_)_._ pSB1A3–*bjaR–gfp–kanR* was prepared by placing the *kanR* gene, in frame, downstream of the *gfp* gene and pBjaR promoter. pSB1A3–*bjaR*_S107R (B0034|CTG)_ *gfp* and pSB1A3–*bjaR*_WT (B0034|ACG)_–*gfp* were prepared by transferring the *bjaR* variant genes identified in the directed evolution screen back into the pSB1A3–*bjaR–gfp* backbone. pSB1A3–*bjaR*_WT (B0031–33|ATG)_–*gfp* were prepared by replacing the BBa_B0034 RBS sequence with other RBSs from the Registry of Standard Biological Parts community library^31^. pSB1A3–*bjaR*_WT (B0034|CTG)_–*gfp* and pSB1A3–*bjaR*_S107R (B0033|ATG)_–*gfp* were prepared by site-directed mutagenesis. Plasmid assembly is described, in full, in the Supplementary Methods. Primer sequences are provided in Supplementary Table 2.

### Preparation of linear DNA templates

Linear DNA templates were obtained by PCR of pPURE plasmids encoding the desired GOI. PCRs were performed with Phusion DNA polymerase master mix using 1 ng plasmid and 500 nM T7 for/Rev1 primers. Reactions were cycled according to the following program: 98 °C for 30 s, 35 cycles (98 °C for 10 s, 54 °C for 20 s and 72 °C for 30 s), 72 °C for 5 min, 4 °C HOLD. PCR products were validated by agarose gel electrophoresis and purified using a QIAquick PCR purification kit. DNA templates were ethanol precipitated and resuspended in Milli-Q H_2_O to 75–100 ng µl^−1^.

### Modification of amine DNA

The PCB-modified forward primer was prepared as described previously^20^. In total, 10 µM T7 for amine was combined with PCB–NHS (final concentration = 5 mM), 100 mM sodium bicarbonate and DMF (final concentration = 50% vol/vol) to a final volume of 50 µl in 500 µl Protein LoBind tubes and incubated in the dark for 3 h with gentle shaking. After 3 h, reactions were quenched with 5 µl (1 M) Tris (pH 8.0). A total of 445 µl Milli-Q H_2_O were added to the tube and then transferred to a 0.5 ml (3 kDa) Amicon column. Samples were centrifuged at 14,000*g* for 15 min, topped up to 500 µl with 10 mM Tris (pH 8.0) and then centrifuged again (three times in total). In total, 2 × 50 µl samples were injected onto a Discovery C-18 column pre-equilibrated with 5% ACN, 95% buffer A (10 mM ammonium acetate) and separated with a 5–35% buffer A:ACN gradient over 40 min, flow rate 2 ml min^−1^. Fractions corresponding with the major peak were pooled, lyophilized, redissolved in 500 µl Milli-Q H_2_O and lyophilized again to remove trace ammonium acetate. Lyophilized oligos were made up in 500 µl Milli-Q H_2_O and washed four times in a 0.5 ml (3 kDa) Amicon centrifugal filter column.

### Formation of LA-DNA

PCB–DNA templates were prepared by PCR with DreamTaq DNA polymerase master mix, 325 nM T7 for PCB/250 nM Rev1 primers, and 1 ng DNA template with the following thermal cycler program: 95 °C for 2 min, 35 cycles (95 °C for 30 s, 49 °C for 30 s and 72 °C for 1 min), 72 °C for 5 min, 4 °C HOLD. PCR products were purified using the QIAquick PCR purification kit, and ethanol was precipitated and resuspended in Milli-Q H_2_O. In total, 1 µg of linear PCB–DNA was added to a 50× molar excess of monovalent streptavidin and made up to 50 ng µl^−1^ with 10 mM Tris (pH 8.0). DNA was incubated with mSA at 24 °C, 600 r.p.m. for 3 h in the dark. Samples were then kept at 4 °C for at least 12 h before use. Amine-modified DNA representing the 100% photocleaved LA-DNA was prepared in the same way, except the T7 for amine primer was used in place of T7 for PCB.

### Gel electrophoresis of LA-DNA

A total of 50 ng of LA-DNA was irradiated with a 365 nm UV lamp (M365L2-C5 (Thorlabs) held 28.5 cm from the samples and collimated to a beam ~2.2 cm in diameter) for 0–15 min at 0.75 mW (measured at the center of the beam using a PM100A power meter console (Thorlabs) and S120VC photodiode sensor (Thorlabs)) and then combined with 6× loading dye and loaded into wells of a 1.5% TAE agarose gel. Gels were run in 1× TAE buffer at 100 V for 1 h and 15 min and then stained in 3× GelRed for up to 1 h. Gels were imaged using a BioRad Geldoc XR+ gel imager. The UV lamp setup described here was maintained in all remaining experiments.

### Bulk CFPS of fluorescent proteins

A total of 3 µl CFPS reactions were prepared with 1× PURExpress and supplemented with 0.8 U µl^−1^ murine RNase inhibitor and 5 ng µl^−1^ linear mNG/mVenus DNA template or 7.5 ng µl^−1^ amine/LA-mNG DNA. As indicated, reactions were irradiated with UV light (0.75 mW) for 1–5 min. Reactions were incubated in a thermal cycler at 37 °C for 4 h and then held at 4 °C for at least 15 min. Reactions were worked up with 47 µl PBS and transferred to Perkin Elmer 384-well black flat-bottom OptiPlates. Fluorescence was measured using a Tecan Infinite M1000 fluorescence plate reader (bandwidth, 5 nm; *z* position, 23,179 µm; Ex_mNG_, 506 nm; Em_mNG_, 517 nm; Ex_mVenus_, 515 nm and Em_mVenus_, 528 nm).

### Imaging chambers

One millimeter holes were drilled through 25 mm × 75 mm microscope slides using a 1 mm diamond tool bit and Dremel 3000 rotary tool to correspond with diagonal corners of 25 µl gene frames. All slides and coverslips (22 mm × 22 mm) were cleaned with 2% decon, isopropanol and Milli-Q H_2_O, then sonicated in Milli-Q H_2_O for at least 10 min and dried under N_2_ flow before use. Coverslips were O_2_ plasma treated for 5 min, before 25 µl gene frames were attached. A total of 0.1% BSA in PBS was added inside the gene frames and incubated at room temperature for 15 min. BSA was removed, and coverslips were washed with the outer solution, twice. The outer solution was removed, and gene frames were applied to the drilled microscope slides. A total of 25 µl of samples were introduced through the drilled holes, and then the holes were sealed with double-sided tape. Samples were incubated coverslip side down (see Supplementary Fig. 16 for illustration).

### Assembly of mNG and LA-mNG synthetic cells

Glass vials (2 ml) were cleaned with isopropanol and then held under vacuum for >1 h. Egg PC dissolved in chloroform (50 mg ml^−1^ stock) was transferred to the vial using Hamilton syringes and then held under a gentle stream of N_2_ to evaporate the chloroform. The vials were tilted at 45° and rotated slowly while held under N_2_ flow to distribute the lipids evenly up the walls. Vials containing lipid films were held under a vacuum in a desiccator for 2–3 h to remove residual chloroform. Mineral oil (filtered through 0.22 µm PES membrane) was added to Egg PC films, by mass, to a final concentration of 5 mg ml^−1^ Egg PC. Vials were vortexed for 1 min and then incubated in a heat block at 80 °C for 10 min with the lids removed. Lids were reapplied to vials and sealed tightly using Teflon tape and parafilm, then vortexed aggressively for 1 min and sonicated in a sonication bath heated to 50 °C for 1 h. Lipid-containing oil was stored at room temperature overnight and vortexed, then sonicated for 10 min at room temperature immediately before use.

A total of 250 µl of 5 mg ml^−1^ Egg PC in mineral oil was transferred to 1.5 ml centrifuge tubes and placed on ice. A total of 5 µl inner solution (PURExpress containing 5–10 ng µl^−1^ DNA, 0.8 U µl^−1^ murine RNase inhibitor, 25 µM TR-dextran (10 kDa) and 200 mM sucrose) was added into the chilled lipid-containing oil, ensuring the tip was constantly moved through the lipid-containing oil to disperse the inner solution. Tubes were passed across a centrifuge rack 3–5 times using light pressure to form cloudy water-in-oil (W/O) emulsions that were placed on ice for ~5 min. Meanwhile, 100 µl of lipid-containing oil was layered on top of 250 µl of chilled outer solution (50 mM HEPES, 400 mM potassium glutamate and 200 mM glucose (pH 7.6)) and placed at room temperature. W/O emulsions were then added on top of this oil phase, and this tube was placed on ice for ~5 min. Centrifuge tubes containing the W/O emulsion above the outer solution were centrifuged at 16,000*g*, 4 °C, for 30 min.

After centrifugation, the oil phase and ~200 µl of the outer solution were removed from the tube and discarded. Using a fresh tip, ~10 µl of the remaining outer solution was ejected against the GUV pellet to displace it from the tube, and the intact GUV pellet was transferred to a new tube containing 250 µl outer solution. The pellet was subsequently resuspended by gently pipetting up and down. Vesicles were centrifuged at 10,000*g*, 4 °C for 10 min, then the outer solution was removed and the pellets were gently resuspended in 25 µl of fresh outer solution.

### *mNG* expression in synthetic cells

To assess *mNG* expression from inside the synthetic cells, the GUV pellets were resuspended in 25 µl outer solution and loaded into homemade imaging chambers. Synthetic cells were incubated in a 37 °C incubator for 2–8 h, coverslip side down. As indicated, LA-mNG SCs were irradiated with UV light for 0–12.5 min at 0.75 mW. Fluorescence microscopy was performed using a Lecia DMi8 inverted epifluorescence microscope using a ×100 oil immersion objective lens. GUVs were imaged using the brightfield, TXR and GFP filters.

### Patterning *mNG* expression in synthetic cells

The pelleted LA-mNG GUVs were resuspended in 15 µl molten 1.5% ultralow gelling point agarose prepared with outer solution, maintained at 28 °C and added into a 25 µl gene frame sandwiched between a glass coverslip and microscope slide. Samples were gelled by placing them at 4 °C for ~30 min. Film photomasks were placed on top of the coverslips and irradiated under the UV lamp for 10 min at 0.75 mW (power adjusted to account for the transparent film containing photomask designs). Samples were incubated at 37 °C for 6 h and then imaged with a Lecia DMi8 inverted epifluorescence microscope using a ×10 objective lens. GUVs were imaged using the brightfield, TXR and GFP filters.

### Image processing

TXR and GFP channel brightness was normalized across all images within a single experiment, and then the separate channels were saved as individual.PNG files. All images corresponding to a single sample were stored within the same directory. ‘Background’ images were created by manually selecting vesicle-free regions of microscopy images (one from each sample within the experiment) and measuring the mean pixel intensity. PNG files were input into the vesicle_analysis script (see https://zenodo.org/record/7729425 for script). Manual analysis of GUVs was performed in ImageJ by creating a region of interest corresponding with the area of an individual vesicle using the circle tool and measuring the diameter and mean pixel intensity within this ROI for both the TXR channel and GFP channel images. Values were exported into an Excel file and plotted using MATLAB.

### IV-HSL synthesis

To a stirring suspension of l-homoserine lactone hydrochloride (100 mg, 0.726 mmol) in acetonitrile (5 ml, 0.145 mmol), diisopropylethylamine was added (0.32 ml, 1.8 mmol, 2.5 eq.). Isovaleryl chloride (0.14 ml, 1.4 mmol, 1.9 eq.) was then added dropwise over 10 min, and the reaction was left for stirring for 16 h. The resulting brown solution was then concentrated in vacuo and subjected to column chromatography (1:1–0:1 PE_40–60_:EtOAc) to yield a white, crystalline solid as the desired product (78 mg, 0.422 mmol 58%). ^1^H-NMR (400 MHz, CDCl_3_) δ/ppm 5.99 (NH, br s, 1H), 4.54 (lac CH, ddd, *J* = 11.6, 8.6, 5.7 Hz, 1H), 4.47 (lac CH, td, *J* = 9.1, 1.3 Hz, 1H), 4.28 (lac CH, ddd, *J* = 11.2, 9.3, 5.9 Hz, 1H), 2.88 (lac CH, dddd, *J* = 12.5, 8.6, 5.8, 1.2 Hz, 1H), 2.21–2.02 (tail CH_2_ + tail CH + lac CH, m, 4H), 1.02–0.92 (CH_3_, m, 6H; Supplementary Fig. 17). MS (ESI^+^) found 208.2, (M + Na)^+^ requires 208.1. Data were in accordance with literature^35^. IV-HSL was dissolved in DMSO and stored at −20 °C.

### M9 minimal media

Supplemented M9 minimal media (1× M9 salts, 0.34 mg ml^−1^ thiamine hydrochloride, 0.2% casamino acids, 2 mM MgSO_4_, 100 µM CaCl_2_ and 0.4% (wt/vol) glucose)^36^ was prepared fresh for every experiment. The media was sterilized by passing it through a 0.22 µm PES syringe filter before use.

### Characterizing BjaR receiver cells

The BjaR reporter plasmids were retransformed into *E. coli* XL10-Gold cells for each experiment. A single colony was used to inoculate 5 ml LB + Amp media and incubated at 37 °C, 225 r.p.m. overnight. Overnight cultures were diluted 1/250 or 1/500 in 5 ml M9 minimal media + Amp and incubated at 37 °C, 225 r.p.m. for 3 h and 45 min (until OD_600_ = ~0.075). Cultures were diluted down to OD_600_ = 0.05 with M9 minimal media + Amp, and 200 µl was added to wells of a 96-well plate containing 1 µl IV-HSL dissolved in DMSO or 1 µl BjaI CFPS reactions (IV-HSL concentrations obtained from serial dilution from 63.2 µM to 632 pM; final DMSO concentration = 0.5%). Cells were incubated at 37 °C, 800 r.p.m., in a thermomixer with a heated lid for 4 h. Fluorescence and OD_600_ were measured in a Tecan M1000 infinite plate reader (excitation, 488 nm; emission, 510 nm; bandwidth, 5 nm; gain, 101 and *z* position, 21,311 µm). The dose–response curves were fitted to the function indicated by Eq. (1), where *a* = maximal fluorescence output, *b* = basal fluorescence output, *X* = concentration of AHSL, GFP = fluorescence at the given condition, EC_50_ = AHSL at 50% maximal fluorescence output and *h* = hill coefficient. EC_50_ and *h* were derived using the nonlinear regression model based on Eq. (1), in MATLAB. A summary of the dose–response data is available in Supplementary Table 1.

1 $${\mathrm{GFP}}=b+\frac{a-b}{1+{10}^{{\rm{log}}\left({\mathrm{EC}}50-X\right)\times h}}$$

### Directed evolution

BjaR underwent four rounds of directed evolution based on a previously published protocol^29^. Briefly, epPCR of the *bjaR* gene was performed in the presence of 100 µM MnCl_2_ using Taq DNA polymerase and 500 nM pSB1A3 for 1 and pSB1A3 Rev1 primers. Reactions were cycled using the following program: 95 °C for 30 s, 30 cycles (95 °C for 15 s, 51 °C for 30 s and 68 °C for 1 min), 68 °C for 5 min, 4 °C HOLD. PCR products were electrophoresed on a 1.2% TAE agarose gel and gel extracted using a Monarch Gel extraction kit. Gel-extracted BjaR DNA was cloned into the pSB1A3 GFP–KanR BB using Gibson’s assembly master mix^37^ and transformed into *E. coli* XL10-Gold cells. Cells were incubated in 500 µl SOC media for 1 h at 37 °C, 225 r.p.m., then 4.5 ml LB + Amp (100 µg ml^−1^ final) was added and cells were incubated overnight at 37 °C, 225 r.p.m. In total, 1 × 10^7^ cells from an overnight culture were transferred into 1 ml M9 minimal + 100 µg ml^−1^ Amp in the presence of IV-HSL (1 nM/0.8 nM/0.6 nM/0.4 nM for rounds 1, 2, 3 and 4, respectively) and incubated for 1 h (37 °C). After 1 h, kanamycin was added to a final concentration of 200 µg ml^−1^ and incubated for a further 5 h. Cells were centrifuged at 8,000*g* for 3 min to remove the selective media, then resuspended in 1 ml LB, repelleted and resuspended in 2 ml LB + Amp. Cells that survived ON-selection were incubated for 2 h, 37 °C. Cells were pelleted, and plasmids were extracted using a PUREyield mini-prep kit. Plasmids from rounds 1/2/3 were used as DNA templates for epPCR in rounds 2/3/4, respectively. After four rounds of evolution, 45 variants were picked and used to inoculate 500 µl LB + 100 µg ml^−1^ Amp in 2 ml deep well plates. Cells were incubated at 37 °C, 250 r.p.m. overnight, and then 5 µl of cells were used to seed 500 µl M9 minimal media + 100 µg ml^−1^ Amp in the remaining wells of the deep well plate. Cells were grown at 37 °C for 3/4 h and then 200 µl were transferred to a lidded, transparent Corning 96-well microplate containing either 1 µl DMSO or 1 µl 200 nM IV-HSL (0.5% DMSO in cell solution). Plates were incubated at 37 °C, 800 r.p.m. in a thermomixer with a heated lid for 4 h. Fluorescence and OD_600_ were measured in a Tecan M1000 infinite plate reader (Ex_GFP_, 488 nm; Em_GFP_, 510 nm; BW, 5 nm and *z* position, 21,311 µm).

### Bulk CFPS of BjaI (^35^S)-methionine labeled

In total, 3 µl CFPS reactions were prepared using PURExpress with 0.8 U µl^−1^ murine RNase inhibitor, 5 ng µl^−1^ linear *bjaI* DNA template and (^35^S)-methionine (0.3 µl, 1,200 Ci mmol^−1^, 15 mCi ml^−1^; MP Biomedicals). As indicated, SAM and IV-CoA were added to 300 µM + 80 µM, respectively. Reactions were incubated in a 37 °C water bath for 3 h and then run on a precast 10% mini protean TGX gel at 200 V for 30 min. The gel was dried onto filter papers using a Biorad Model583 gel dryer, exposed to Kodak Biomax MS film overnight and then developed.

### IV-HSL biosynthesis by BjaI

In total, 3 µl CFPS reactions were prepared using PURExpress with 0.8 U µl^−1^ murine RNase inhibitor, 5 ng µl^−1^ linear *bjaI* DNA template and 300 µM SAM and 80 µM IV-CoA, as indicated. Reactions were incubated for 5 h at 37 °C using a thermal cycler and then serially diluted in Milli-Q H_2_O. In total, 1 µl of the samples was added to lidded Corning 96-well plates containing 200 µl BjaI receiver cells (pSB1A3 BjaR_S107R (CTG)_ at OD_600_ = 0.05). Cells were grown at 37 °C, 800 r.p.m. in a thermomixer with a heated lid for 4 h. OD_600_ and fluorescence were measured using a Tecan infinite M1000 plate reader (Ex, 488 nm; Em, 510 nm; bandwidth, 5 nm; *z* position, 23,179 µm and gain, 101).

### BjaI expression in synthetic cells

Synthetic cells were assembled as described above, except the inner solution was prepared without TR-dextran and contained 10 ng µl^−1^ linear *bjaI* DNA, 80 µM IV-CoA and 300 µM SAM. Samples were resuspended in 25 µl outer solution and incubated in centrifuge tubes at 37 °C, 250 r.p.m. for 5 h. The BjaI synthetic cells were then serially diluted and assayed against BjaI receiver cells, as described above. In synthetic cells prepared at 0.75× and 0.5× PURExpress concentrations and 50 mM sucrose, the outer solution was adjusted accordingly (50 mM HEPES pH 7.6, 300 mM potassium glutamate, 50 mM glucose and 50 mM HEPES pH 7.6, 200 mM potassium glutamate, 50 mM glucose, respectively).

### Agarose pad induction of receiver cells

Microscope slides were first cleaned with Milli-Q H_2_O and isopropanol. In total, 2 × 25 µl gene frames were attached to a single microscope slide with the open side still covered with the transparent film. Molten 1.5% ultralow gelling point agarose was mixed with SCs or 10 nM IV-HSL as required and added inside the gene frames. A second microscope slide was carefully slid on top of the gene frames to distribute the molten agarose throughout the frame. Microscope slides sandwiching the gene frame were transferred to the fridge for 1 h to gel the agarose, and then the top slide was carefully removed by moving it off the gene frame horizontally. In total, 3 µl BjaR_S107R (B0034|CTG)_ receiver cells at OD_600_ = 0.1 were added on top of the gelled agarose pads, and the excess liquid was allowed to evaporate for ~5 min. The transparent film was removed from the gene frame and an isopropanol-cleaned, plasma-treated coverslip was placed on top of the agarose pad to seal it. Samples were irradiated with 0.75 mW UV light for 7.5 min, then incubated at 37 °C for 6 h and imaged using a Lecia DMi8 inverted epifluorescence microscope using a 10x objective lens.

### Reporting summary

Further information on research design is available in the Nature Portfolio Reporting Summary linked to this article.

## Online content

Any methods, additional references, Nature Portfolio reporting summaries, source data, extended data, supplementary information, acknowledgements, peer review information; details of author contributions and competing interests; and statements of data and code availability are available at 10.1038/s41589-023-01374-7.

## Supplementary information

Supplementary Information Supplementary Figs. 1–17, Supplementary Tables 1 and 2, Supplementary Methods and Supplementary Note–plasmid sequences.

Reporting Summary

## Data Availability

All the data generated in this study are available within the article, the supplementary information, figures and source data. Source data for the supplementary figures are available from https://zenodo.org/record/7808487. Source data are provided with this paper.

## Source data

Source Data Fig. 1 Numerical data indicating the mean GFP channel intensity of individual GUVs that were identified using a circular Hough transform.
Source Data Fig. 3 Numerical data representing the OD_600_, fluorescence intensity and calculated OD-normalized fluorescence intensity for BjaR receiver cell dose–response curves. Data for each subfigure are given on a separate sheet.
Source Data Fig. 4 Numerical data representing the OD_600_, fluorescence intensity and calculated OD-normalized fluorescence intensity for BjaR receiver cell dose–response curves using biosynthetic IV-HSL containing samples. Data for each subfigure are given on a separate sheet. An uncropped image of the autoradiograph indicating the location of the *bjaI* produced by CFPS. An uncropped image of the Coomassie-stained SDS–PAGE gel of the bjaI CFPS reactions.

## Code availability

The custom vesicle analysis script developed and used in this study is available at https://zenodo.org/record/7729425.

## References

[1] Buddingh BC, van Hest JCM (2017). Artificial cells: synthetic compartments with life-like functionality and adaptivity. Acc. Chem. Res..

[2] Noireaux V, Libchaber A (2004). A vesicle bioreactor as a step toward an artificial cell assembly. Proc. Natl Acad. Sci. USA.

[3] Gaut NJ (2022). Programmable fusion and differentiation of synthetic minimal cells. ACS Synth. Biol..

[4] Lentini R (2014). Integrating artificial with natural cells to translate chemical messages that direct *E. coli* behaviour. Nat. Commun..

[5] Lentini R (2017). Two-way chemical communication between artificial and natural cells. ACS Cent. Sci..

[6] Adamala KP, Martin-Alarcon DA, Guthrie-Honea KR, Boyden ES (2017). Engineering genetic circuit interactions within and between synthetic minimal cells. Nat. Chem..

[7] Ding Y, Contreras-Llano LE, Morris E, Mao M, Tan C (2018). Minimizing context dependency of gene networks using artificial cells. ACS Appl. Mater. Interfaces.

[8] Rampioni G (2018). Synthetic cells produce a quorum sensing chemical signal perceived by *Pseudomonas aeruginosa*. Chem. Commun..

[9] Niederholtmeyer H, Chaggan C, Devaraj NK (2018). Communication and quorum sensing in non-living mimics of eukaryotic cells. Nat. Commun..

[10] Tang TD (2018). Gene-mediated chemical communication in synthetic protocell communities. ACS Synth. Biol..

[11] Toparlak OD (2020). Artificial cells drive neural differentiation. Sci. Adv..

[12] Smith JM, Chowdhry R, Booth MJ (2021). Controlling synthetic cell-cell communication. Front. Mol. Biosci..

[13] Lentini R (2013). Fluorescent proteins and in vitro genetic organization for cell-free synthetic biology. ACS Synth. Biol..

[14] Dwidar M (2019). Programmable artificial cells using histamine-responsive synthetic riboswitch. J. Am. Chem. Soc..

[15] Lynch SA, Gallivan JP (2009). A flow cytometry-based screen for synthetic riboswitches. Nucleic Acids Res..

[16] Espah Borujeni A, Mishler DM, Wang J, Huso W, Salis HM (2016). Automated physics-based design of synthetic riboswitches from diverse RNA aptamers. Nucleic Acids Res..

[17] Elani Y, Law RV, Ces O (2014). Vesicle-based artificial cells as chemical microreactors with spatially segregated reaction pathways. Nat. Commun..

[18] Dupin A, Simmel FC (2019). Signalling and differentiation in emulsion-based multi-compartmentalized in vitro gene circuits. Nat. Chem..

[19] Schroeder A (2012). Remotely activated protein-producing nanoparticles. Nano Lett..

[20] Booth MJ, Schild VR, Graham AD, Olof SN, Bayley H (2016). Light-activated communication in synthetic tissues. Sci. Adv..

[21] Booth MJ, Restrepo Schild V, Box SJ, Bayley H (2017). Light-patterning of synthetic tissues with single droplet resolution. Sci. Rep..

[22] Lindemann A (2011). Isovaleryl-homoserine lactone, an unusual branched-chain quorum-sensing signal from the soybean symbiont *Bradyrhizobium japonicum*. Proc. Natl Acad. Sci. USA.

[23] Pautot S, Frisken BJ, Weitz DA (2003). Production of unilamellar vesicles using an inverted emulsion. Langmuir.

[24] Olins PO, Rangwala SH (1989). A novel sequence element derived from bacteriophage T7 mRNA acts as an enhancer of translation of the *lacZ* gene in *Escherichia coli*. J. Biol. Chem..

[25] Venancio-Marques A (2012). Modification-free photocontrol of β-lactam conversion with spatiotemporal resolution. ACS Synth. Biol..

[26] Gonzales DT, Yandrapalli N, Robinson T, Zechner C, Tang TD (2022). Cell-free gene expression dynamics in synthetic cell populations. ACS Synth. Biol..

[27] Sakamoto R, Noireaux V, Maeda YT (2018). Anomalous scaling of gene expression in confined cell-free reactions. Sci. Rep..

[28] Tekel SJ (2019). Engineered orthogonal quorum sensing systems for synthetic gene regulation in *Escherichia coli*. Front. Bioeng. Biotechnol..

[29] Kimura Y, Kawai-Noma S, Saito K, Umeno D (2020). Directed evolution of the stringency of the LuxR *Vibrio fischeri* quorum sensor without OFF-state selection. ACS Synth. Biol..

[30] Hecht A (2017). Measurements of translation initiation from all 64 codons in *E. coli*. Nucleic Acids Res..

[31] Weiss, R. et al. Ribosome binding sites/prokaryotic/constitutive/community collection. https://parts.igem.org/Ribosome_Binding_Sites/Prokaryotic/Constitutive/Community_Collection (Accessed 15 June 2023).

[32] Raychaudhuri A, Jerga A, Tipton PA (2005). Chemical mechanism and substrate specificity of RhlI, an acylhomoserine lactone synthase from *Pseudomonas aeruginosa*. Biochemistry.

[33] Kylilis N, Tuza ZA, Stan GB, Polizzi KM (2018). Tools for engineering coordinated system behaviour in synthetic microbial consortia. Nat. Commun..

[34] Du P (2020). De novo design of an intercellular signaling toolbox for multi-channel cell-cell communication and biological computation. Nat. Commun..

[35] Helen, B. et al. Synthetic ligands that modulate the activity of the RhlR quorum sensing receptor. https://patentscope.wipo.int/search/en/detail.jsf?docId=WO2017190116 (2017).

[36] Endy:M9 media/supplemented. https://openwetware.org/wiki/Endy:M9_media/supplemented (2007).

[37] Gibson DG (2009). Enzymatic assembly of DNA molecules up to several hundred kilobases. Nat. Methods.

