# Supplementary material for: Engineering cellular communication between light-activated synthetic cells and bacteria
Source: Nat Chem Biol. 2023 Jul 6;19(9):1138–46. doi: 10.1038/s41589-023-01374-7 (PMC10449621; doi:10.1038/s41589-023-01374-7)
Supplement: Supplementary file 1 — Supplementary Figs. 1–17, Supplementary Tables 1 and 2, Supplementary Methods and Supplementary Note–plasmid sequences. [file 41589_2023_1374_MOESM1_ESM.pdf]

nature chemical biology

Article

https://doi.org/10.1038/s41589-023-01374-7

Engineering cellular communication between light-activated synthetic cells and bacteria

In the format provided by the authors and unedited

Supplementary figures

1 Formation of GUVs by emulsion droplet transfer . . . 4
2 Improving the CFPS of mNG . . . 5
3 mNG expression inside GUVs . . . 6
4 Quantifying mNG expression inside GUVs . . . 7
5 Light-activated DNA preparation . . . 8
6 Light-activated DNA characterisation . . . 9
7 Light-activated synthetic cells vs DNA template concentration . . . 10
8 Light-activated synthetic cells vs UV light exposure time . . . 11
9 Patterned LA-SC activation with complex photomasks . . . 12
10 BjaR reporter plasmid . . . 13
11 Screening BjaR mutants generated by directed evolution . . . 14
12 BjaR reporter plasmid RBS screen . . . 15
13 Activation of BjaR receiver cells with non-cognate AHSLs . . . 16
14 Diluted PURExpress vs IV-HSL biosynthesis . . . 17
15 Synthetic cells in M9 agarose pads . . . 18
16 Imaging chambers . . . 19
17 IV-HSL NMR . . . 20

Supplementary information

0.1 Supplementary table 1 22
0.2 Plasmid sequences 23
0.2.1 pPURE-*mNG* (Linear) 23
0.2.2 pPURE-*gp10(1-9)::mNG* (Linear) 23
0.2.3 pPURE-T7g10-*gp10(1-9)::mNG* (Linear) 24
0.2.4 pPURE-*mVenus* (Linear) 24
0.2.5 pPURE-T7g10-*gp10(1-9)::mVenus* (Linear) 25
0.2.6 pPURE-*bjaI* (Linear) 25
0.2.7 pSB1A3-*bjaR-gfp* 26
0.2.8 pSB1A3-*bjaR*$_{KO}$-*gfp* 27
0.2.9 pSB1A3-*bjaR-gfp-kanR* 28
0.2.10 pSB1A3-*bjaR*$_{KO}$-*gfp-kanR* 30
0.2.11 pSB1A3-*bjaR-gfp* (B0031 | ATG) 31
0.2.12 pSB1A3-*bjaR-gfp* (B0032 | ATG) 33
0.2.13 pSB1A3-*bjaR-gfp* (B0033 | ATG) 34
0.2.14 pSB1A3-*bjaR-gfp* (B0034|CTG) 35
0.2.15 pSB1A3-*bjaR-gfp* (B0034 | ACG) 37
0.2.16 pSB1A3-*bjaR*$_{S107R}$-*gfp* (B0034 | CTG) 38
0.2.17 pSB1A3-*bjaR*$_{S107R}$-*gfp* (B0033 | ATG) 39
0.3 Supplementary methods 41

0.3.1 pPURE-*mNG* assembly 41

0.3.2 pPURE-T7g10-*gp10(1-9)::mNG* assembly 41

0.3.3 pPURE-*gp10(1-9)::mNG* assembly 42

0.3.4 pPURE-T7g10-*gp10(1-9)::mVenus* assembly 42

0.3.5 pPURE-*bjaI* assembly 43

0.3.6 pSB1A3-$bjaR_{KO}$-*gfp* assembly 43

0.3.7 pSB1A3-*bjaR-gfp-kanR* and pSB1A3-$bjaR_{KO}$-*gfp-kanR* assembly 43

0.3.8 pSB1A3-$bjaR_{\mathrm{S107R}}$-*gfp* (B0034 | CTG) assembly 44

0.3.9 pSB1A3-*bjaR-gfp* (B0034 | ACG) assembly 44

0.3.10 pSB1A3-*bjaR-gfp* (B0031/B0032/B0033 | ATG) assembly 45

0.3.11 pSB1A3-*bjaR-gfp*(B0034 | CTG) assembly 45

0.3.12 pSB1A3-$bjaR_{\mathrm{S107R}}$-*gfp* (B0033 | ATG) assembly 45

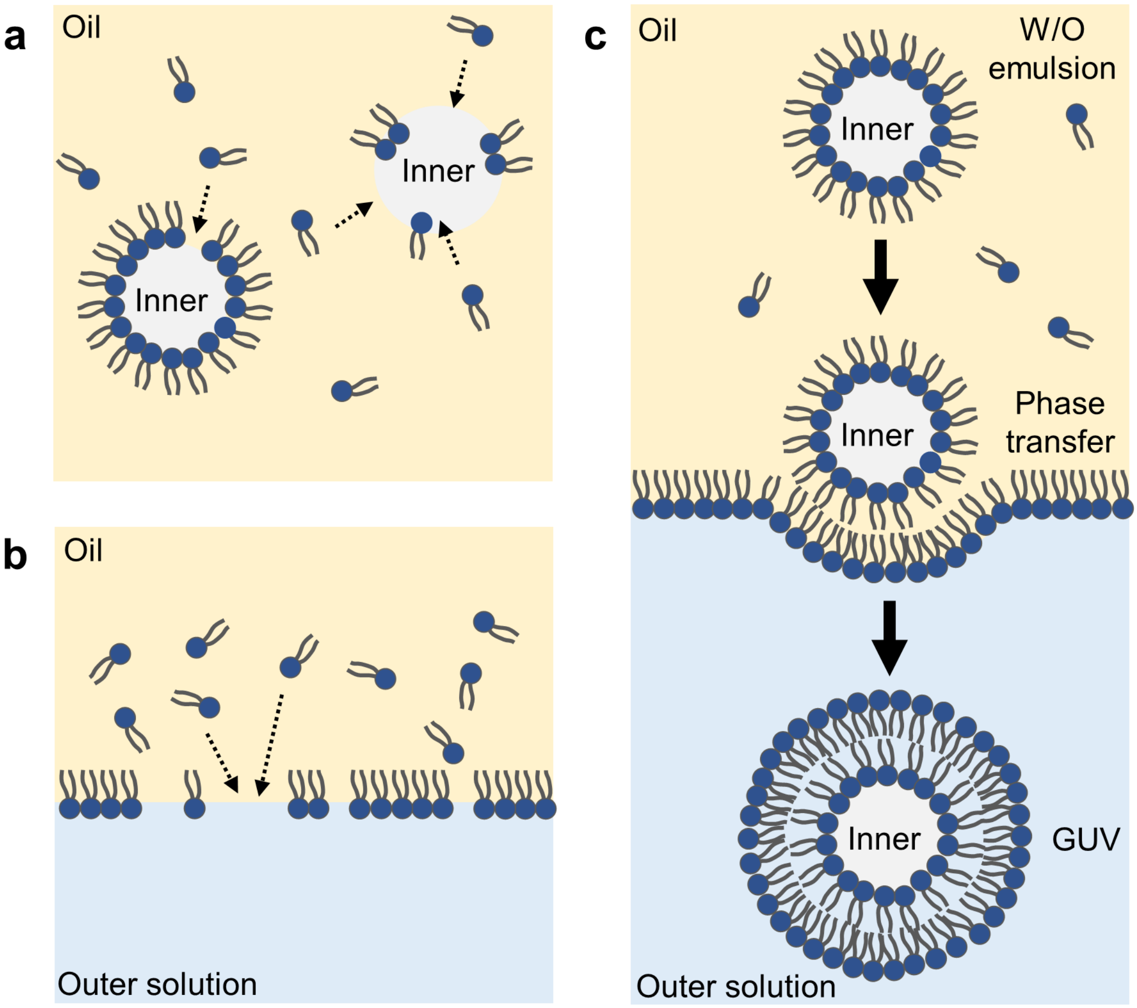

Supplementary figure 1: Formation of GUVs by emulsion droplet transfer
a) An aqueous sucrose-containing inner solution was emulsified in a lipid-containing oil to create creating droplets stabilised by a lipid monolayer. **b)** Lipid-containing oil was placed on top of an aqueous solution containing glucose to create a second lipid monolayer at the W/O interface. **c)** Emulsion droplets were pulled through the second lipid monolayer into the outer solution by centrifugation.

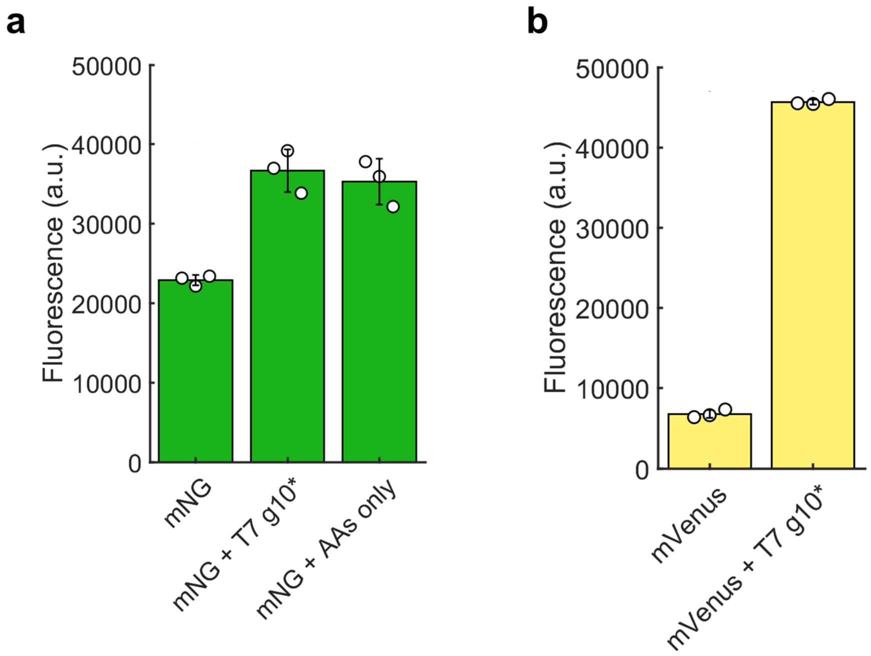

Supplementary figure 2: Improving the CFPS of mNG
a) Installing the T7 g10 leader sequence into the 5' UTRs of linear DNA templates encoding *mNG* increased the fluorescence output bulk CFPS reactions by 1.6-fold. Interestingly, the same improvements in fluorescence output were also observed when only the 9 amino acid leader (MASMTGGQQ; ATGGCTAGCATGACTGGTGGACAGCAA), was introduced at the 5' end of the *mNeonGreen* gene. **b)** The T7 g10 leader sequence also improved the CFPS of mVenus template used in our previous work[1] 7-fold, suggesting a context dependency - improvements in gene expression depended on the gene and/or amino acids encoded immediately downstream. mNG was selected over mVenus due to its superior brightness and better compatibility with the fluorescence microscope GFP filter wavelengths. Bar and error bars indicate the mean and s.d. of three independent experiments (n=3), represented by open circles.

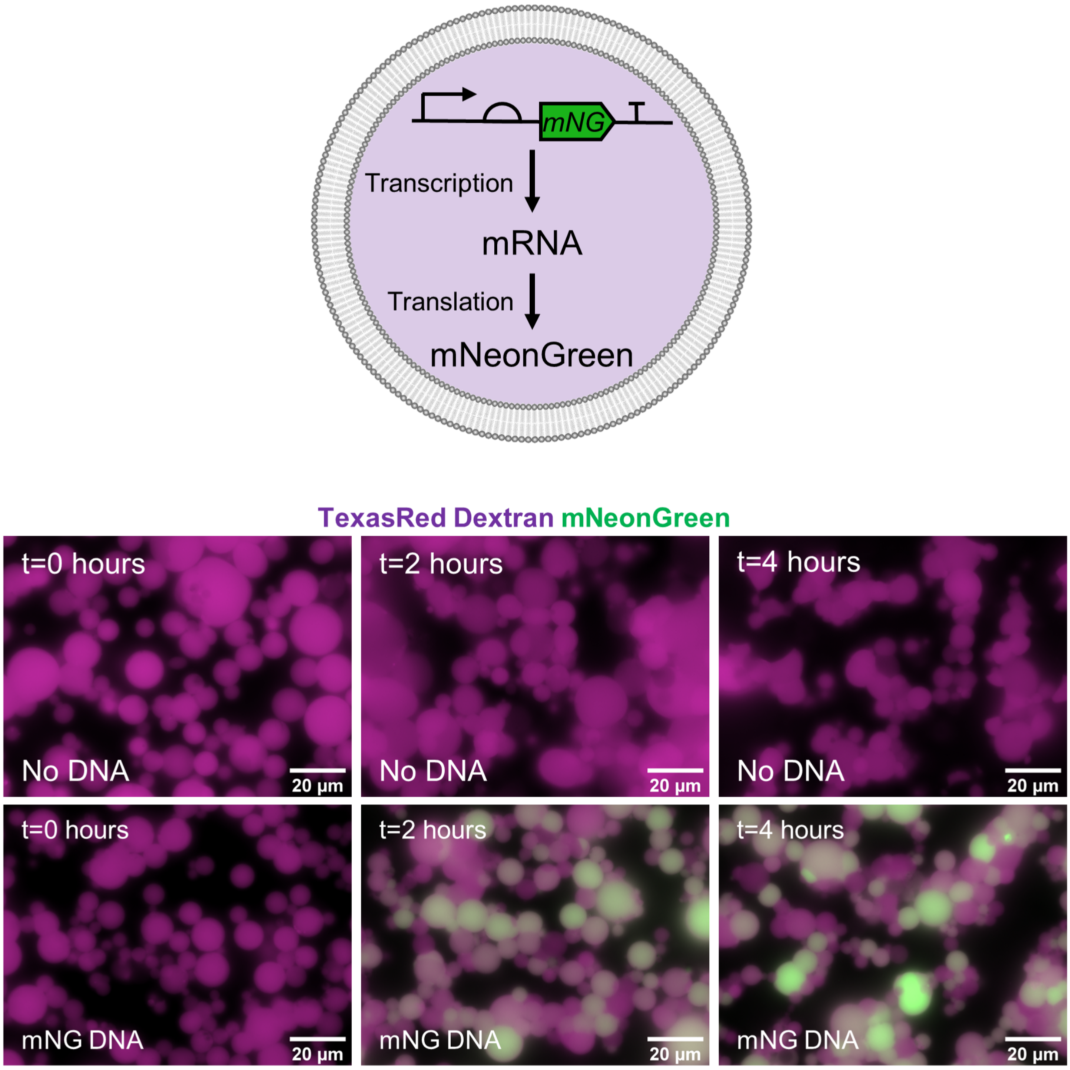

Supplementary figure 3: mNG expression inside GUVs
Fluorescence microscopy images confirmed mNG was expressed inside GUVs. mNG fluorescence intensity increased over time until components of the CFPS were depleted, and expression began to saturate. Images representative of n=3 independent experiments.

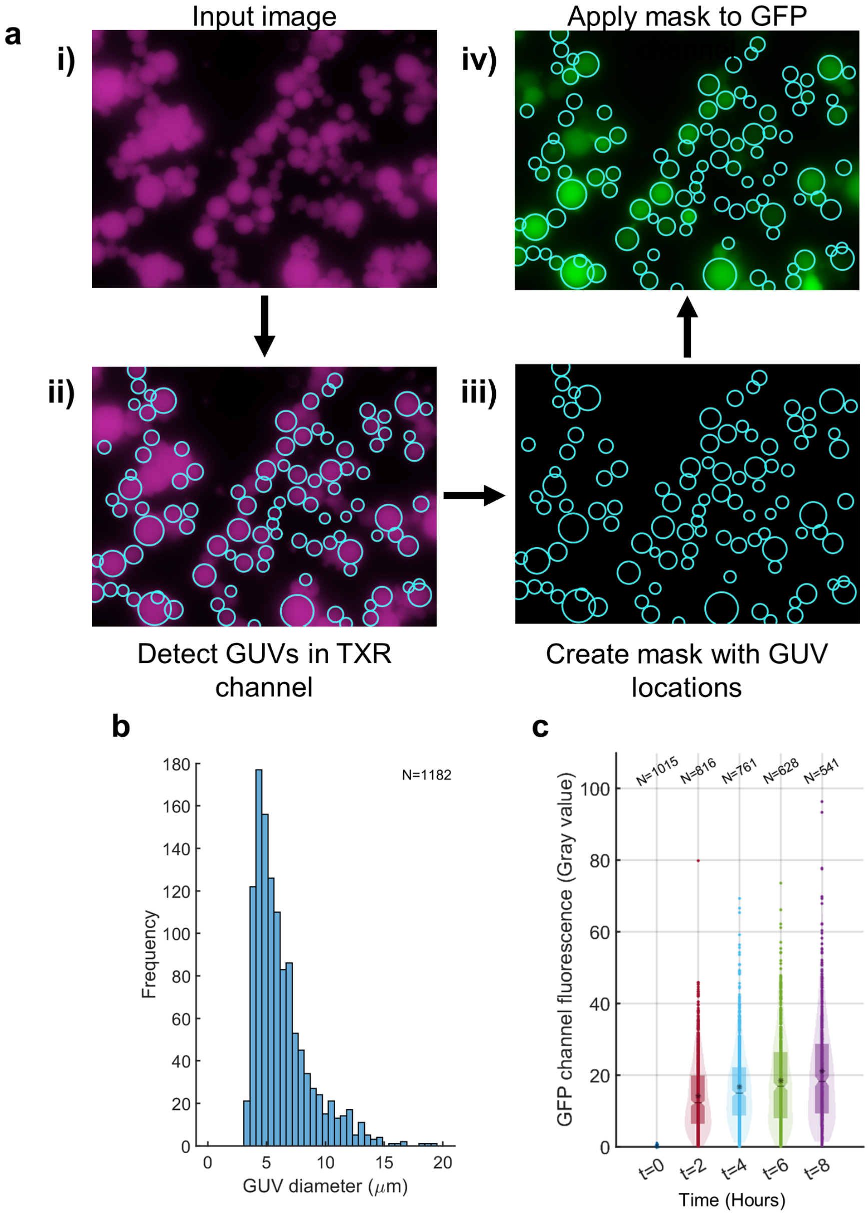

Supplementary figure 4: Quantifying mNG expression inside GUVs
a) TexasRed labelled GUVs present in TXR channel fluorescence microscopy images were used as an input for circular Hough transform. The origin and radii of each GUV were identified and used to create maps. Maps were then applied to the corresponding GFP channel image and mNG expression was determined by extracting the mean pixel intensity inside the individual GUVs. **b)** Histograms indicating the typical size distribution of GUVs prepared by emulsion droplet transfer. Data represents the total number of vesicles detected from 10 microscopy images obtained at t=0 hours, within a single experiment. **c)** Violin plots illustrating mean pixel intensity of individual vesicles vs incubation time. These plots were consistent with the fluorescence microscopy images and confirmed that almost all DNA containing vesicles had a fluorescence value above background (No DNA GUV fluorescence), but *mNG* expression was highly variable. *mNG* expression increased with incubation time, as expected. Plot represents individual vesicles at different time points within a single time series experiment. The box plot minima, maxima, notch, and asterisk indicate the lower quartile, upper quartile, mean, and median, respectively.

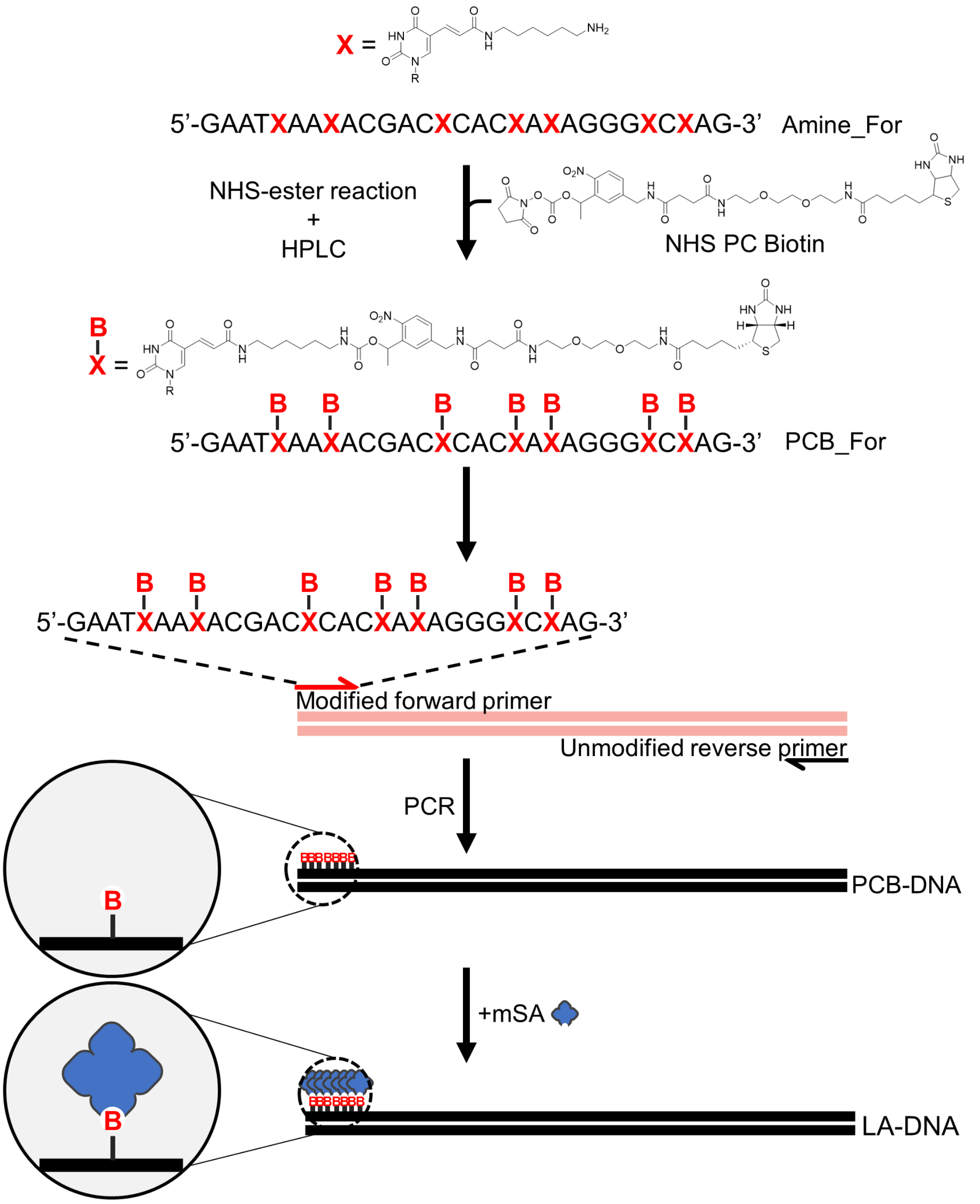

Supplementary figure 5: Light-activated DNA preparation
Sequence of the T7 For oligonucleotide with the positions of the amine-modified thymine bases (shown above) labelled as X. An NHS-PCB molecule was reacted with amine groups within the DNA and oligos with all seven amines modified were separated and purified from the partially modified oligos by HPLC. The fully modified oligo was used as a forward primer in PCR to create linear DNA templates with PCB groups positions in the T7 promoter sequence. mSA was bound to the PCB in the resulting DNA to form LA-DNA containing the photocaged T7 promoter.

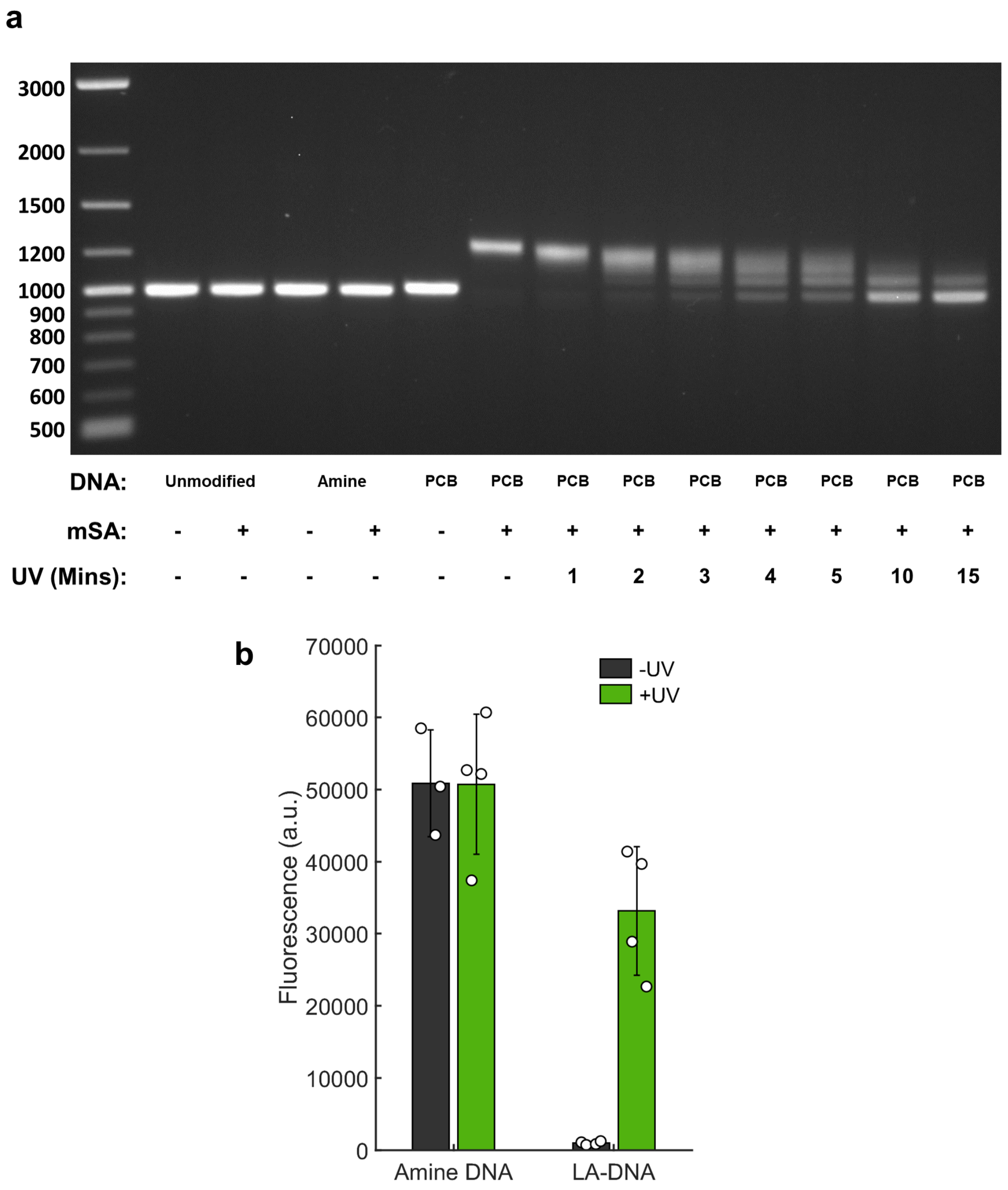

Supplementary figure 6: Light-activated DNA characterisation
a) DNA templates containing PCB groups in the T7 promoter successfully bound mSA, forming LA-DNA. LA-DNA had an altering electrophoretic mobility, which caused a band shift compared to PCB DNA. No band shift occurred for unmodified or amine modified DNA, therefore these DNA templates did not bind mSA. mSA remained stably fixed at the promoter until the 2-nitrobenzyl group within the PCB linkers underwent photolysis in a UV dose-dependent manner. DNA laddering reflects the removal of subsets of the 7 blocking groups. Most blocking groups were removed after 15 mins irradiation. Image is representative of n=3 independent experiments. **b)** Fluorescence output of bulk CFPS reactions containing either 7.5 ng/μL LA-DNA or amine DNA templates (DNA containing only the amino-C6-thymine bases in the T7 promoter to represent 100% photocleavage) encoding mNG. Minimal mNG was expressed in the absence of UV light; gene expression was repressed by >98% relative to amine DNA templated reactions. When the reactions were exposed to UV light (0.75 mW UV light for 3 minutes), light-activated mNG expression reached ~70% of that achieved by the amine DNA templated reactions. Bars and error bars represent the mean and s.d of n=4 independent experiments, represented by the open circles.

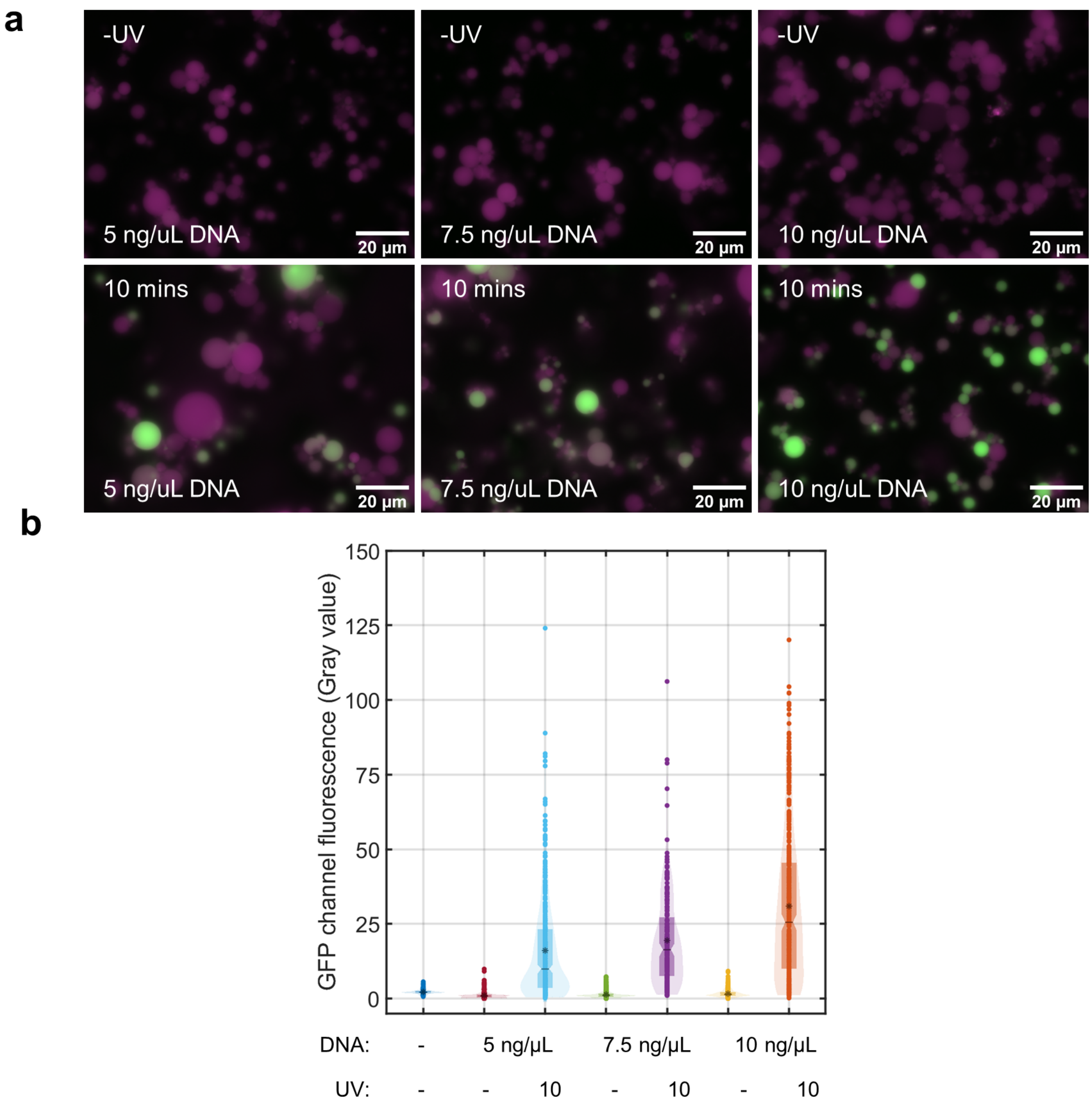

DNA conc	5 ng/µL		7.5 ng/µL		10 ng/µL	
UV (Mins)	-	10	-	10	-	10
% of vesicles with mean pixel intensity > mean(No DNA) ± 3SD	3.80	70.4	4.82	86.7	3.38	87.3
Median pixel intensity	0.792	9.87	0.968	16.3	1.30	25.5
Fold increase	12.5		16.9		20.1	

Supplementary figure 7: Light-activated synthetic cells vs DNA template concentration **a)** Epifluorescence microscopy images of synthetic cells prepared with 5 ng/μL, 7.5 ng/μL or 10 ng/μL LA-mNG DNA and treated with either no UV light or 0.75 mW UV light for 10 minutes. Images are representative of each sample across a single experiment. **b)** Quantification of single vesicle fluorescence for all conditions shown in the microscopy images. The box plot minima, maxima, notch, and asterisk indicate the lower quartile, upper quartile, mean, and median, respectively. Data represents individual samples within a single experiment.

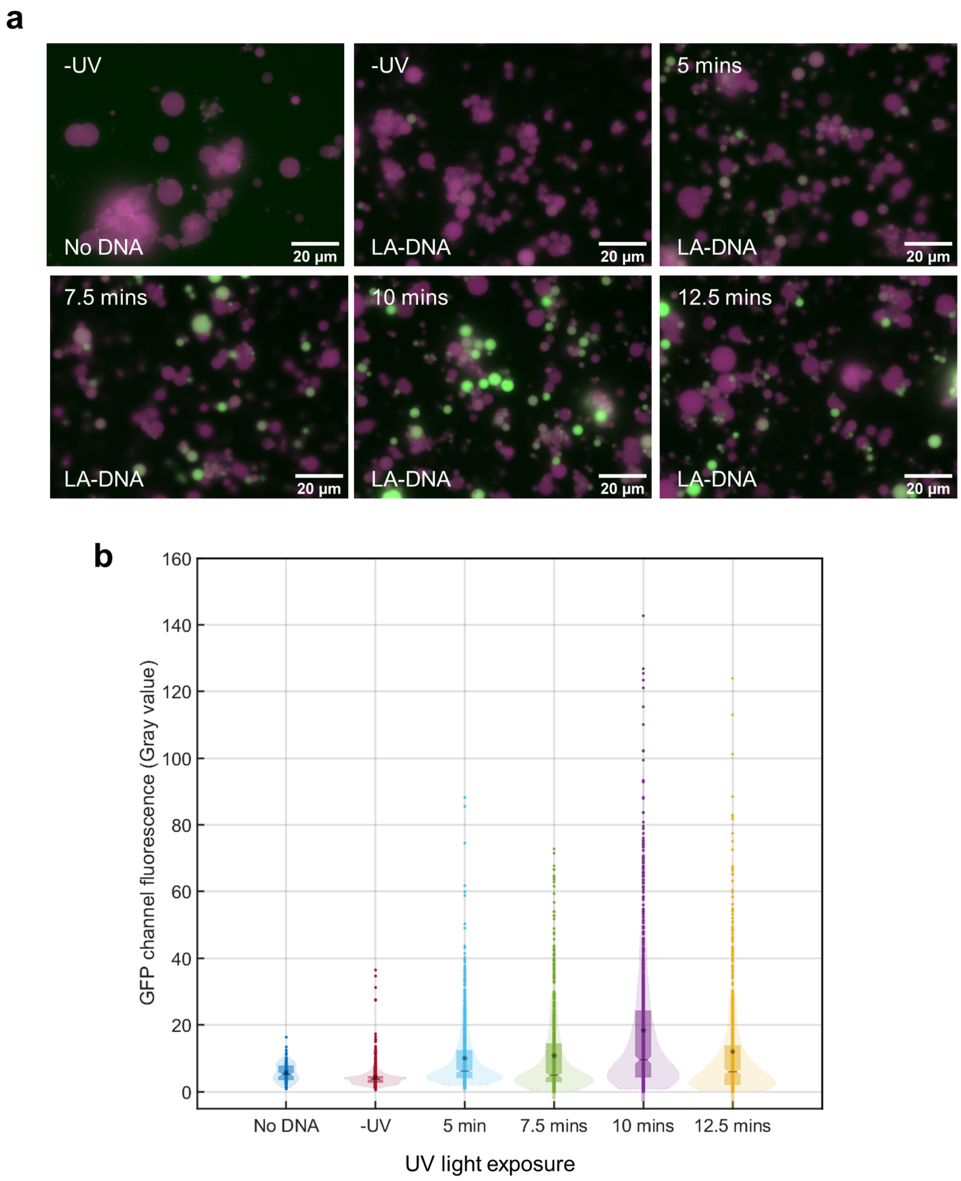

Supplementary figure 8: Light-activated synthetic cells vs UV light exposure time
a) Epifluorescence microscopy images of synthetic cells prepared with 5 ng/μL LA-mNG DNA and treated with 0.75 mW UV light of increasing duration. **b)** Quantification of single vesicle fluorescence for all conditions shown in the microscopy images, compared to the respective UV light exposure conditions. The box plot minima, maxima, notch, and asterisk indicate the lower quartile, upper quartile, mean, and median, respectively. Data represents individual samples within a single experiment.

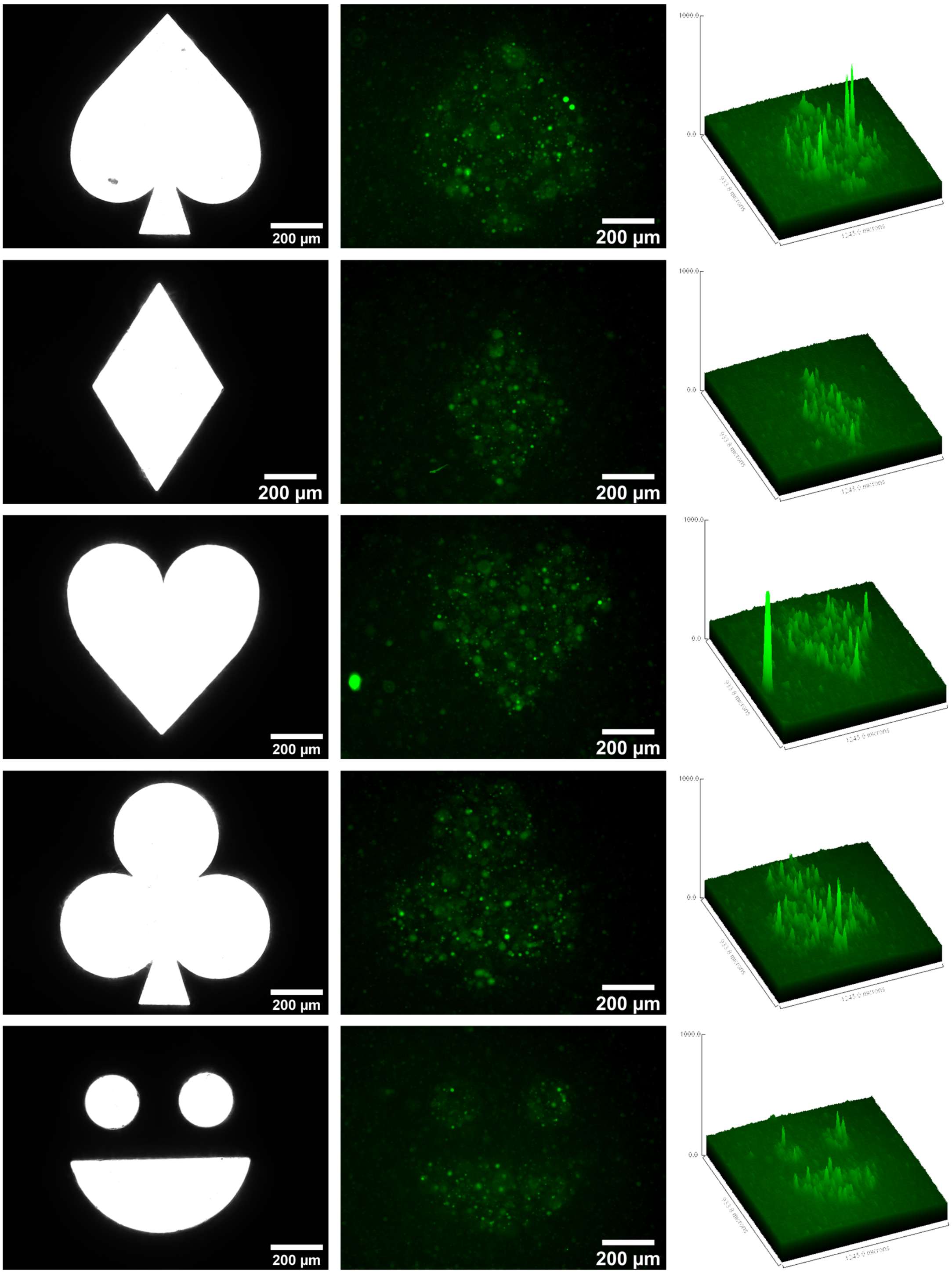

Supplementary figure 9: Patterned LA-SC activation with complex photomasks
LA-mNG synthetic cells were immobilised in 1.5% ULGP agarose and irradiated with patterned UV light according to photomask designs. mNG was expressed inside GUVs only in the UV exposed areas. Highly patterning fidelity and resolution was demonstrated using the irregular photomask designs that contained features with different dimensions. Images are representative of n=3 independent experiments.

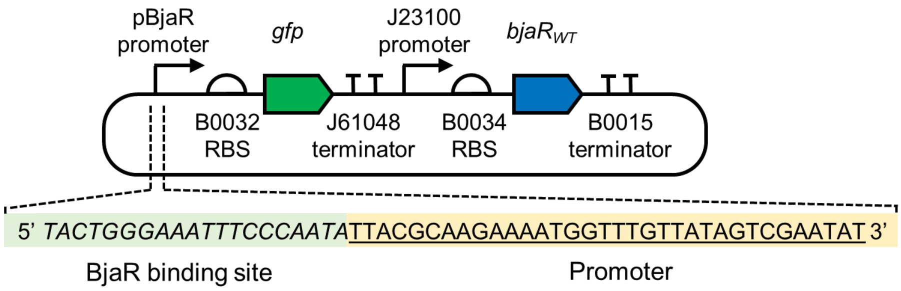

Supplementary figure 10: BjaR reporter plasmid
pSB1A3-*bjaR-gfp* plasmid. *bjaR* was expressed under the control of a constitutively active promoter (J23100). *gfp* expression was regulated via the pBjaR promoter, and activated upon addition of IV-HSL. The pBjaR promoter comprised the BjaR binding site (Green) and the core pLux promoter sequence (Amber).

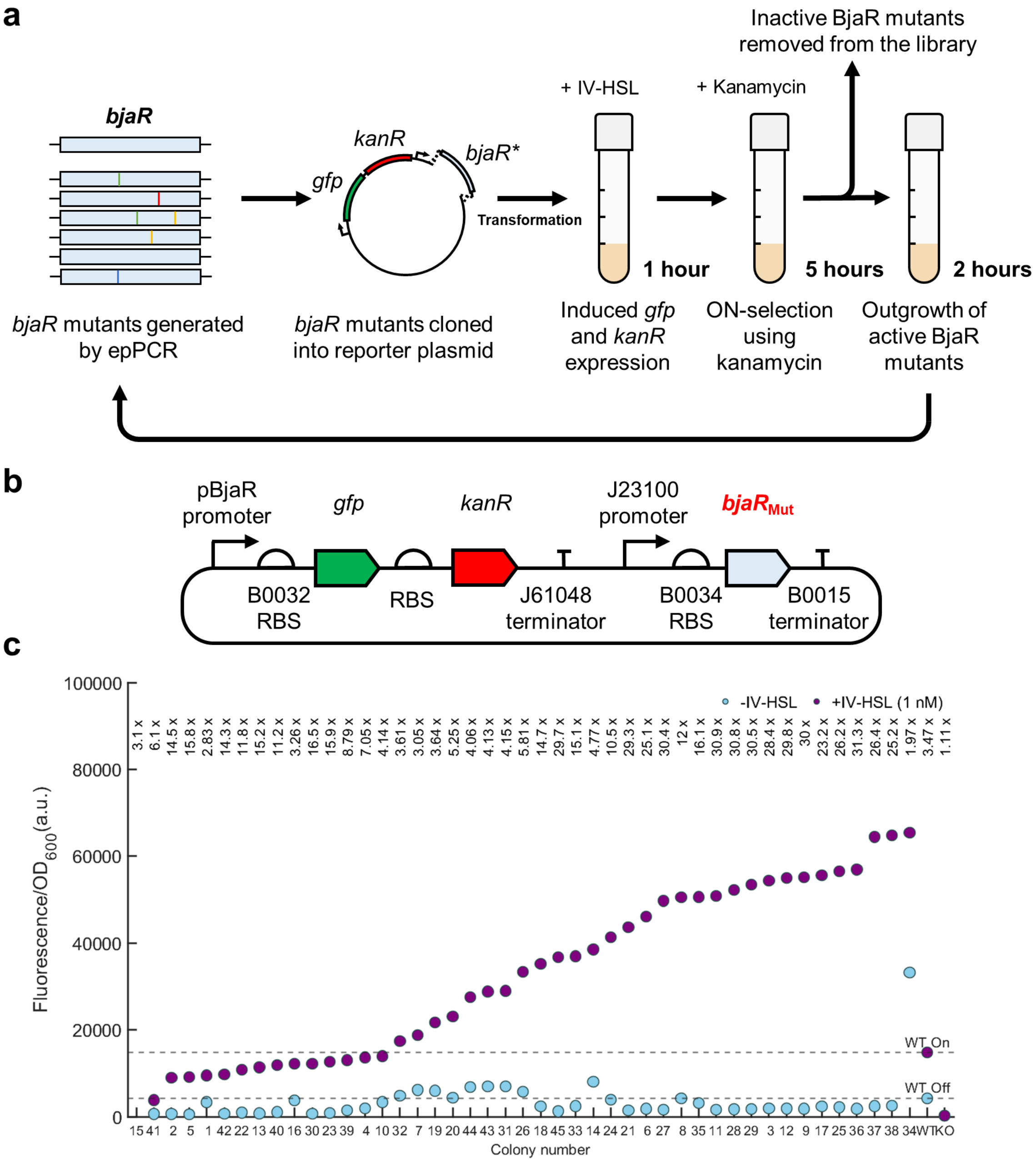

Supplementary figure 11: Screening BjaR mutants generated by directed evolution
a) The directed evolution workflow used to improve BjaR stringency.**b)** The pSB1A3-*bjaR*-*gfp*-*kanR* plasmid was constructed to enable active BjaR mutants to be recovered after ON-selection in Kanamycin. *kanR* was inserted directed downstream of the *gfp* gene to create a bicistronic mRNA. Both *gfp* and *kanR* expression were activated by the addition of IV-HSL. **c)** 45 colonies containing pSB1A3-$bjaR_{\mathrm{mut}}$-*gfp*-*kanR* plasmids were grown in M9 minimal media in the absence and presence of IV-HSL (1 nM). Receiver cell fluorescence was used as a measure of gene expression from the pBjaR promoter. pSB1A3-*bjaR*-*gfp*-*kanR* and pSB1A3$bjaR_{\mathrm{KO}}$-*gfp*-*kanR* were included to ON/OFF state enable comparison. Many colonies demonstrated a lower OFF-state and/or improved ON-state compared to pSB1A3-*bjaR*-*gfp*-*kanR* after four rounds of directed evolution. The fold change in fluorescence output is given above each respective colony.

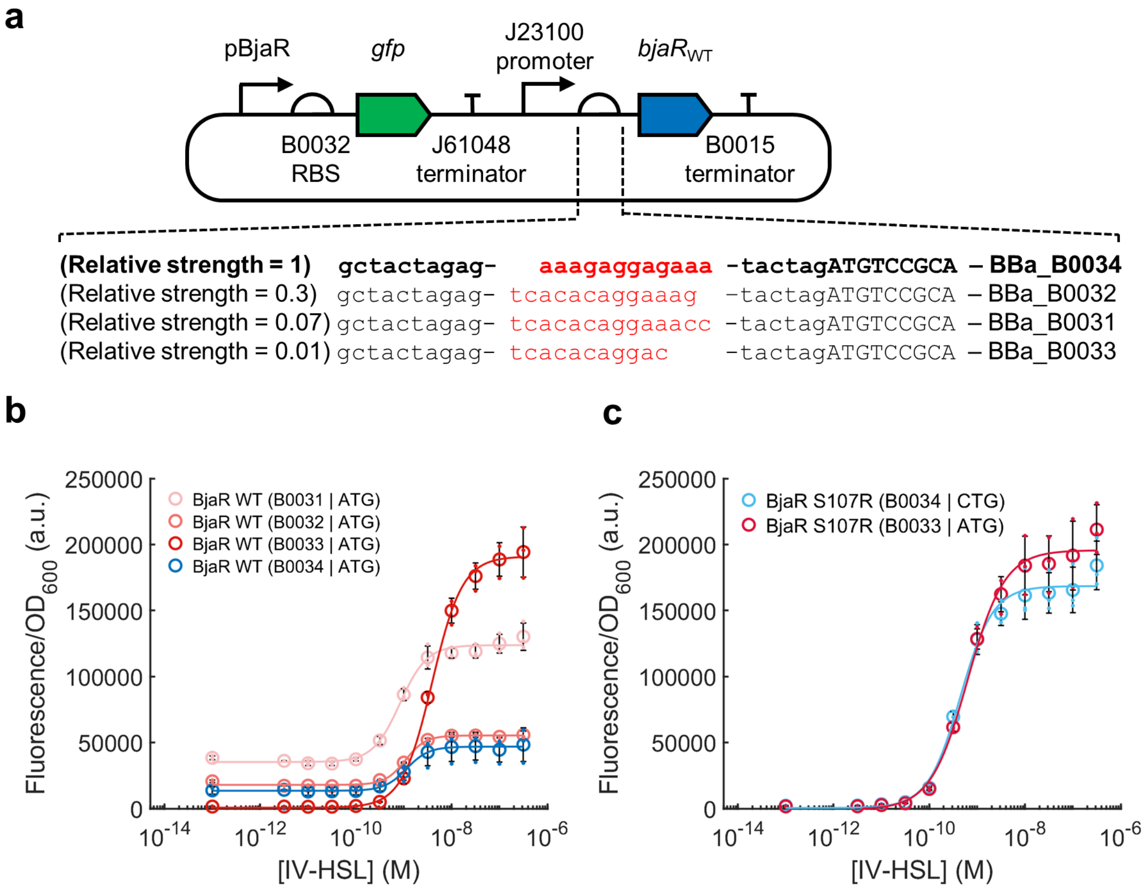

Supplementary figure 12: BjaR reporter plasmid RBS screen
a) The BBa_B0034 RBS in the pSB1A3-*bjaR-gfp* plasmid was replaced by RBSs with decreasing relative strengths to decrease the abundance of BjaR in the cell. **b)** The reporter plasmid containing the weakest RBS (BBa_B0033) showed the best dose-response behaviour and closely resembled the CTG start codon mutant. **c)** Combining the BBa_B0033 RBS and S107R mutation in the *bjaR* gene replicated $\text{BjaR}_{\text{S107R (B0034 | CTG)}}$ dose-response behaviour, except $\text{BjaR}_{\text{S107R (B0033 | ATG)}}$ achieved greater maximum fluorescence output. Open circles and error bars in b) and c) indicate the mean and s.d. of n=3 independent experiments.

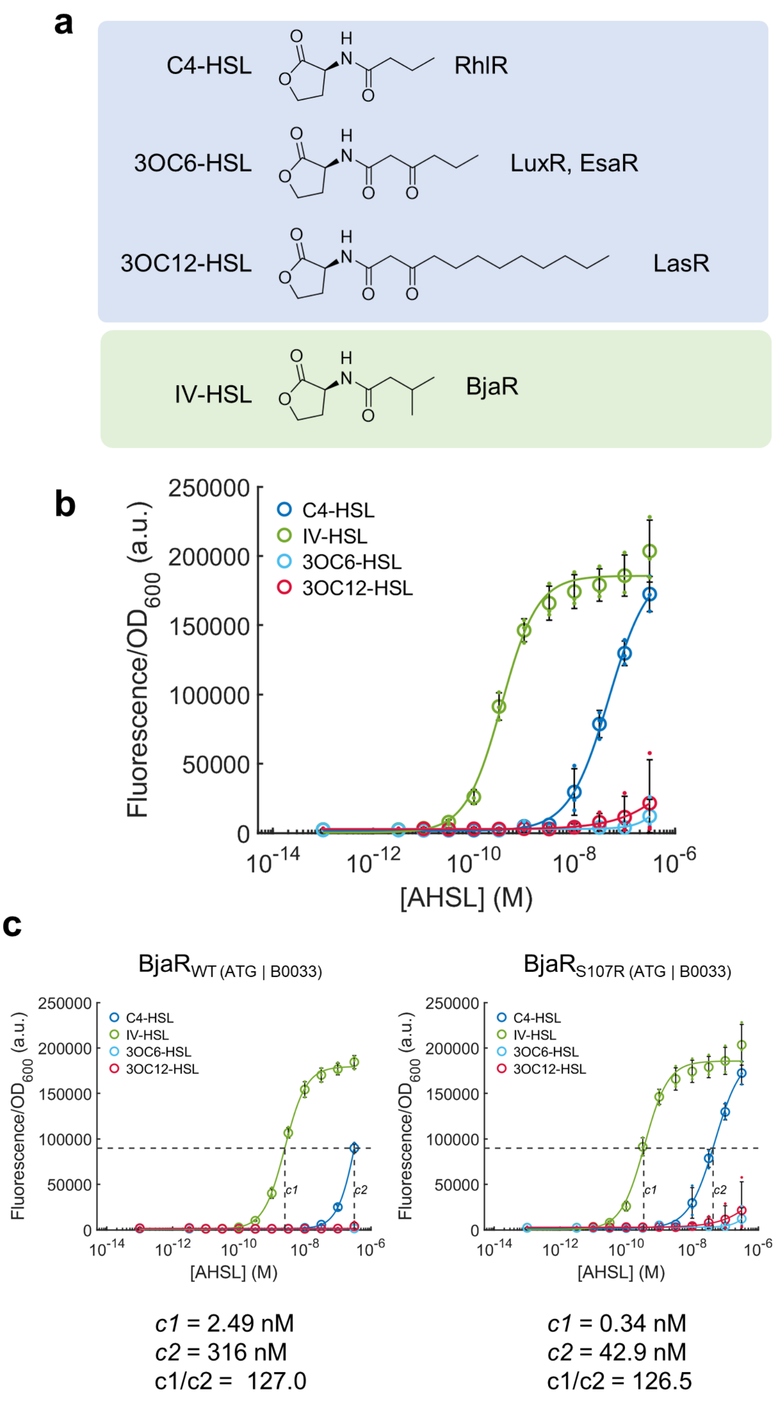

Supplementary figure 13: Activation of BjaR receiver cells with non-cognate AHSLs
a) The structures of AHSLs used by various QS systems that were tested against the BjaR receiver cells. **b)** Dose-response curves of each respective AHSL against $\text{BjaR}_{\text{S107R (B0033 | ATG)}}$ receiver cells. Each curve moved towards lower AHSLs proportionally with one another, compared to the $\text{BjaR}_{\text{WT (B0033 | ATG)}}$ receiver cells. **c)** WT and S107R receiver cells both demonstrated ~126-fold higher sensitivity for IV-HSL over C4-HSL, therefore, the S107R mutation does not appear to influence the substrate selectivity of BjaR. Open circles and error bars in b) and c) indicate the mean and s.d. of n=3 independent experiments.

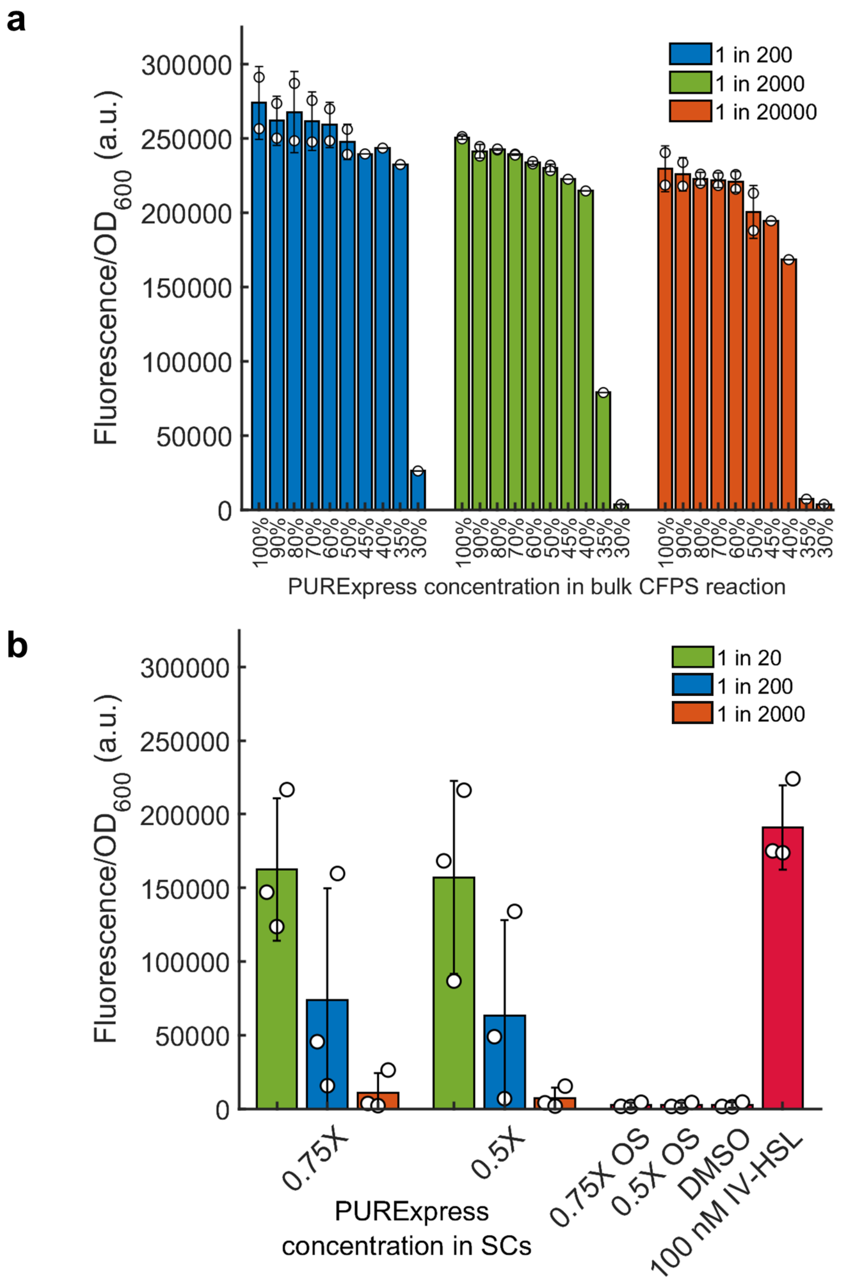

Supplementary figure 14: Diluted PURExpress vs IV-HSL biosynthesis
a) BjaI CFPS reactions that were prepared with 5 ng/μL *bjaI* DNA, 300 μM SAM, 80 μM SAM, but varying concentrations of SolA and SolB PURExpress solutions (1X refers to the volumes of SolA and SolB recommended by NEB) were used to induce *gfp* expression in BjaR$_{S107R\ (CTG\ |\ B0034)}$ receiver cells. In bulk CPFS reactions, IV-HSL synthesis after 5 hours incubation at 37 °C was only slightly perturbed when PURExpress components were provided at 0.4X the recommended concentrations. **b)** BjaI-expressing synthetic cells prepared with 0.75X and 0.5X PURExpress successfully synthesised IV-HSL *in situ* and activated *gfp* expression in BjaR$_{S107R\ (CTG\ |\ B0034)}$ receiver cells. 1 in 20, 1 in 200, 1 in 2000, and 1 in 20000 refer to the final concentrations of the bulk CFPS reaction in the cell cultures; i.e. 1 in 200 means 1 μL of CFPS reaction was added to 200 μL of cells. Bars and error bars in a) indicate the mean and s.d. of n=2 independent experiments. Bars and error bars in b) indicate the mean and s.d. of n=3 independent experiments.

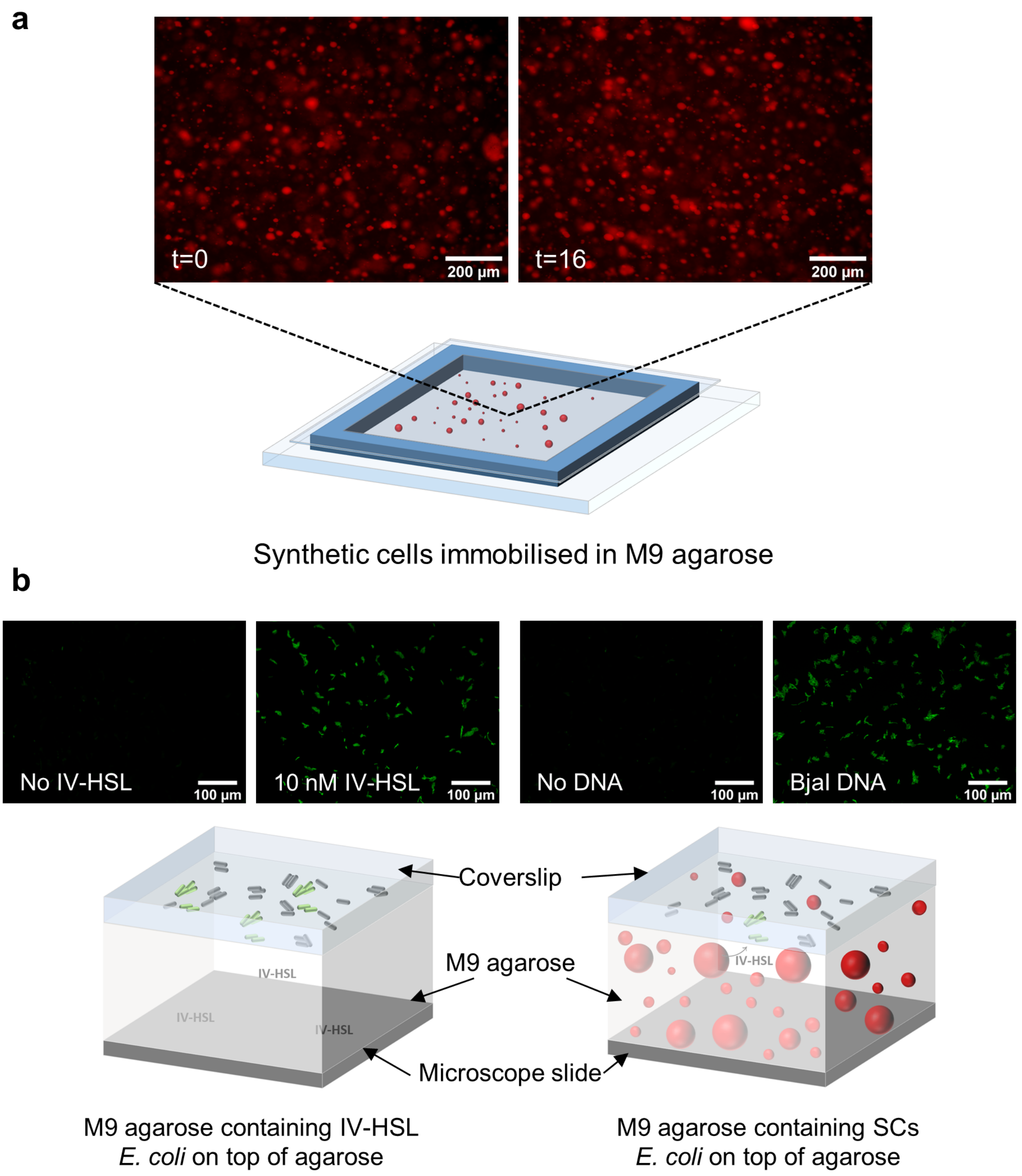

Supplementary figure 15: Synthetic cells in M9 agarose pads
a) Reduced osmolarity synthetic cells prepared with 0.5X PURExpress and 25 μM TexasRed-Dextran were resuspended in molten 1.5% agarose prepared with M9 minimal media, then set to form agarose pads using gene frames. There was no noticeable increase in background fluorescence nor decrease in the number of synthetic cells present after 16 hours incubation at 37 °C. Images are representative of n=3 experiments. **b)** BjaR receiver cells were added on top of agarose pads prepared with or without synthetic IV-HSL (10 nM), or pads containing synthetic cells with no DNA template or BjaI DNA. BjaR receiver cells expressed similar amounts of GFP on synthetic IV-HSL pads and BjaI synthetic cell pads confirming the production of IV-HSL and its diffusion across the GUV lipid bilayers and into the agarose. Images are representative of n=2 independent experiments.

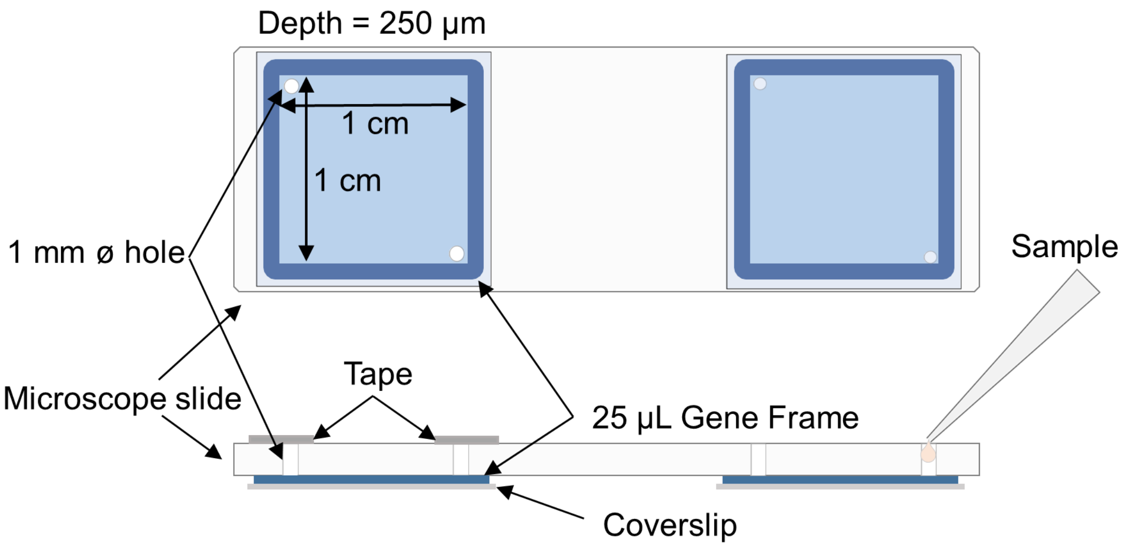

Supplementary figure 16: Imaging chambers
25 μL gene frames were placed onto O_2 plasma treated 25 mm x 25 mm coverslips, then passivated with BSA. The second side of the gene frame was then sealed onto the drilled coverslips, with opposite corners of the frame aligned with the holes. Sample was introduced through one inlet hole, then both were sealed using double sided tape.

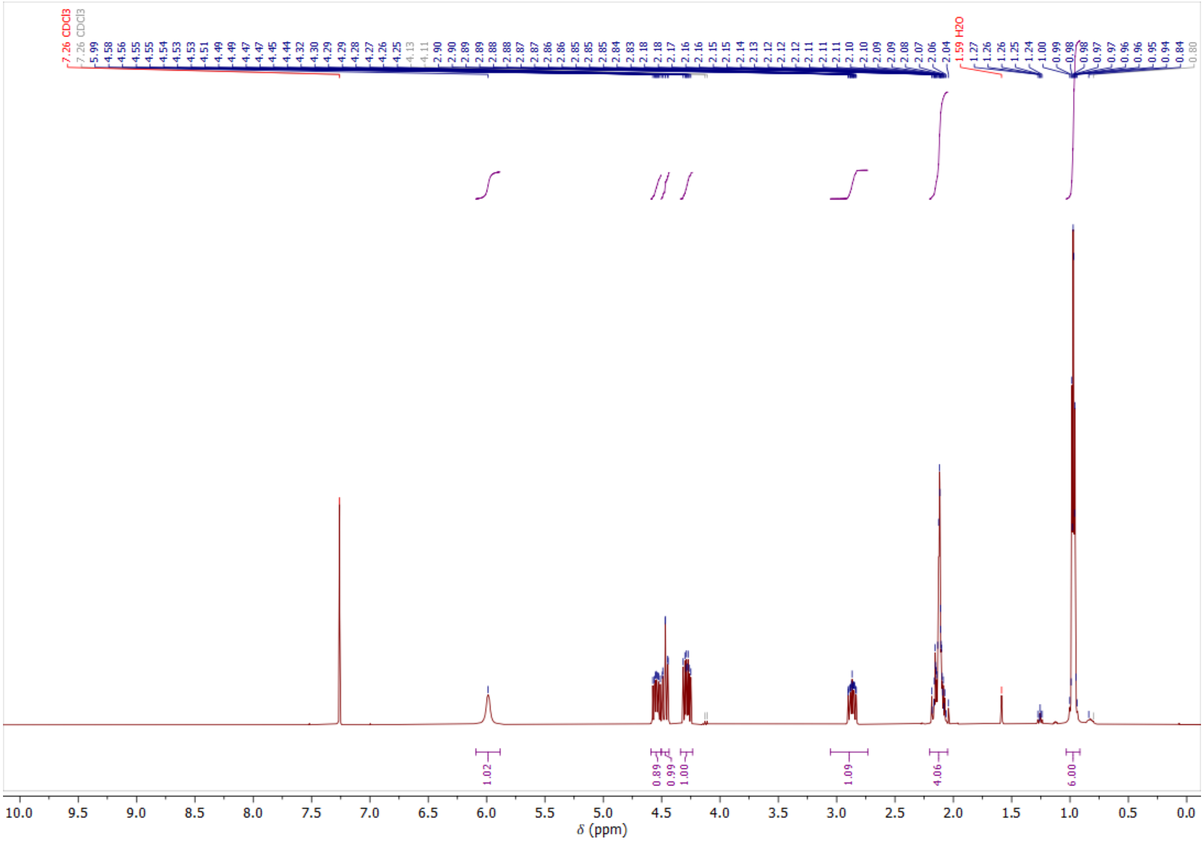

Supplementary figure 17: IV-HSL NMR
NMR of the synthetic IV-HSL used as a standard in BjaR receiver cell characterisation.

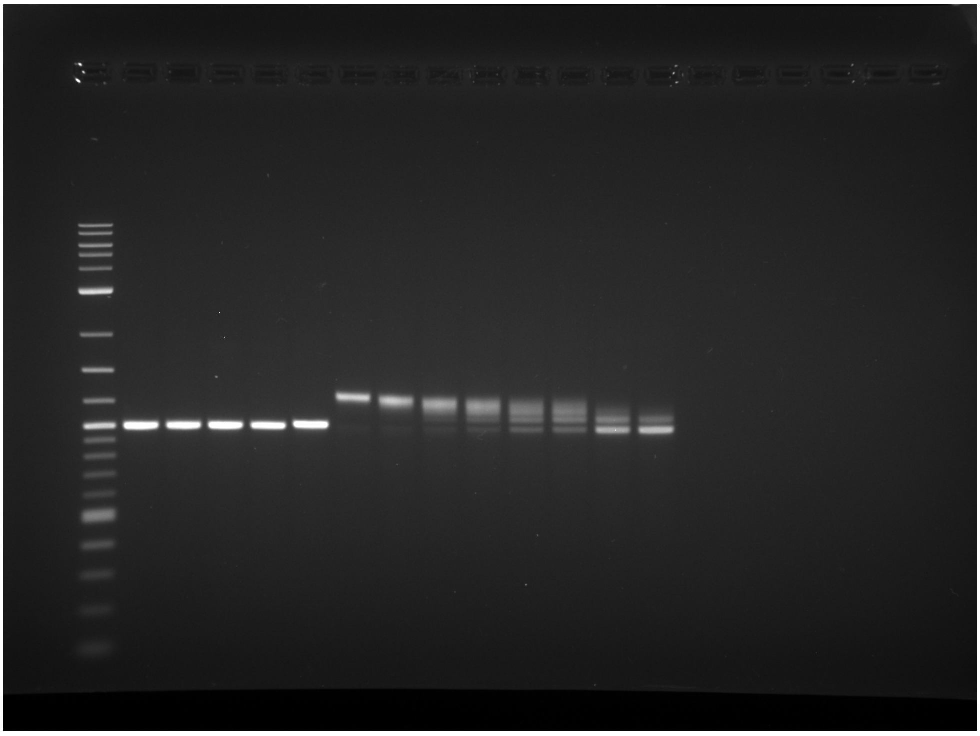

Uncropped gel from supplementary figure 6a. Ladder = 1 kb plus (NEB).

0.1 Supplementary table 1

Receiver cell type	AHSL	Basal fluorescence output	Max fluorescent output	EC50	Hill coefficient	Dynamic range
BjaR $_{\text{KO (B0034} \mid \text{ATG)}}$	IV-HSL	321 ± 29	390 ± 42	N/A	N/A	N/A
BjaR $_{\text{WT (B0034} \mid \text{ATG)}}$	IV-HSL	17993 ± 693	51540 ± 1890	22.3 ± 2.6 nM	1.74	3.41x
BjaR $_{\text{WT (B0034} \mid \text{ACG)}}$	IV-HSL	479 ± 114	147842 ± 7664	1.00 ± 0.11 nM	1.25	308.5x
BjaR $_{\text{WT (B0034} \mid \text{CTG)}}$	IV-HSL	2055 ± 198	197481 ± 4181	2.68 ± 0.48 nM	1.41	96.1x
BjaR $_{\text{WT (B0031} \mid \text{ATG)}}$	IV-HSL	33948 ± 1466	132626 ± 4734	0.83 ± 0.71 nM	1.54	3.9x
BjaR $_{\text{WT (B0032} \mid \text{ATG)}}$	IV-HSL	16895 ± 379	63195 ± 8873	1.08 ± 0.91 nM	2.06	3.7x
BjaR $_{\text{WT (B0033} \mid \text{ATG)}}$	IV-HSL	1305 ± 257	194262 ± 18964	3.93 ± 0.38 nM	1.38	148.8x
BjaR $_{\text{S107R (B0034} \mid \text{CTG)}}$	IV-HSL	2089 ± 228	184153 ± 18428	0.44 ± 0.04 nM	1.28	88.15x
BjaR $_{\text{S107R (B0033} \mid \text{ATG)}}$	IV-HSL	1570 ± 179	211248 ± 18832	0.63 ± 0.10 nM	1.13	134.5x
BjaR $_{\text{WT (B0033} \mid \text{ATG)}}$	C4-HSL	1116 ± 34	89784 ± 5037	N/A	N/A	80.4x
BjaR $_{\text{WT (B0033} \mid \text{ATG)}}$	3OC6-HSL	1013 ± 96	1379 ± 299	N/A	N/A	N/A
BjaR $_{\text{WT (B0033} \mid \text{ATG)}}$	3OC12-HSL	1028 ± 94	1339 ± 477	N/A	N/A	N/A
BjaR $_{\text{S107R (B0033} \mid \text{ATG)}}$	C4-HSL	1936 ± 193	172442 ± 12675	48.5 ± 1.98 nM	1.1	89.1x
BjaR $_{\text{S107R (B0033} \mid \text{ATG)}}$	3OC6-HSL	1948 ± 300	12099 ± 12020	N/A	N/A	N/A
BjaR $_{\text{S107R (B0033} \mid \text{ATG)}}$	3OC12-HSL	2595 ± 667	21449 ± 31327	N/A	N/A	N/A

Table 1: Dose-response metrics
This table represents EC50, hill coefficients, and dynamic ranges from the fitted dose response curves for various receiver cells and analytes.

0.2 Plasmid sequences

0.2.1 pPURE-*mNG* (Linear)

gaaattaatacgactcactatagggtctagaaataattttgtttaactttaagaaggaggtatacatATGGTGAGCAAAGG
CGAAGAGGATAATATGGCAAGCCTGCCTGCAACACATGAACTGCATATTTTTGGT
AGCATTAACGGCGTGGATTTTGATATGGTTGGTCAAGGCACCGGTAATCCGAATG
ATGGTTATGAAGAACTGAATCTGAAAAGCACCAAAGGCGATCTGCAGTTTAGCCC
GTGGATTCTGGTTCCGCATATTGGTTATGGTTTTCATCAGTATCTGCCGTATCCG
GATGGTATGAGCCCGTTTCAGGCAGCAATGGTTGATGGTAGCGGTTATCAGGTTC
ATCGTACCATGCAGTTTGAAGATGGTGCAAGCCTGACCGTTAATTATCGTTATAC
CTATGAAGGCAGCCACATTAAAGGTGAAGCACAGGTTAAAGGTACAGGTTTTCCG
GCAGATGGTCCGGTTATGACCAATAGTCTGACCGCAGCAGATTGGTGTCGTAGCA
AAAAAACCTATCCGAACGATAAAACCATCATCAGCACCTTCAAATGGTCATATAC
CACCGGCAATGGTAAACGTTATCGTAGCACCGCACGTACCACCTATACCTTTGCA
AAACCGATGGCAGCAAACTATCTGAAAAATCAGCCGATGTATGTGTTTCGCAAAA
CGGAACTGAAACATTCCAAAACCGAGCTGAACTTTAAAGAATGGCAGAAAGCATT
TACCGATGTGATGGGTATGGATGAGCTGTACAAATAATGAggatcccgggaattctcgagtaag
gttaacctgcaggaggcctttaattaaggtggtgcggccgcgctagcggtcccgggggatcgatccggctgctaacaaagcccgaaag
gaagctgagttggctgctgccaccgctgagcaataactagcataaccccttggggcctctaaacgggtcttgaggggttttttgctgaaa
ggaggaactatatc

0.2.2 pPURE-*gp10(1-9)::mNG* (Linear)

gaaattaatacgactcactatagggtctagaaataattttgtttaactttaagaaggaggtatacatATGGCTAGCATGAC
TGGTGGACAGCAACATATGGTGAGCAAAGGCGAAGAGGATAATATGGCAAGCCT
GCCTGCAACACATGAACTGCATATTTTTGGTAGCATTAACGGCGTGGATTTTGAT
ATGGTTGGTCAAGGCACCGGTAATCCGAATGATGGTTATGAAGAACTGAATCTGA
AAAGCACCAAAGGCGATCTGCAGTTTAGCCCGTGGATTCTGGTTCCGCATATTGG
TTATGGTTTTCATCAGTATCTGCCGTATCCGGATGGTATGAGCCCGTTTCAGGCA
GCAATGGTTGATGGTAGCGGTTATCAGGTTCATCGTACCATGCAGTTTGAAGATG
GTGCAAGCCTGACCGTTAATTATCGTTATACCTATGAAGGCAGCCACATTAAAGG
TGAAGCACAGGTTAAAGGTACAGGTTTTCCGGCAGATGGTCCGGTTATGACCAAT
AGTCTGACCGCAGCAGATTGGTGTCGTAGCAAAAAAACCTATCCGAACGATAAAA
CCATCATCAGCACCTTCAAATGGTCATATACCACCGGCAATGGTAAACGTTATCG
TAGCACCGCACGTACCACCTATACCTTTGCAAAACCGATGGCAGCAAACTATCTG
AAAAATCAGCCGATGTATGTGTTTCGCAAAACGGAACTGAAACATTCCAAAACCG
AGCTGAACTTTAAAGAATGGCAGAAAGCATTTACCGATGTGATGGGTATGGATGA
GCTGTACAAATAATGAggatcccgggaattctcgagtaaggttaacctgcaggaggcctttaattaaggtggtgcggcc
gcgctagcggtcccgggggatcgatccggctgctaacaaagcccgaaaggaagctgagttggctgctgccaccgctgagcaataacta
gcataaccccttggggcctctaaacgggtcttgaggggttttttgctgaaaggaggaactatatc

0.2.3 pPURE-T7g10-*gp10(1-9)::mNG* (Linear)

gaaattaatacgactcactatagggtctagggagaccacaacggtttccctctagaaataattttgtttaactttaagaaggagatatac
cATGGCTAGCATGACTGGTGGACAGCAACATATGGTGAGCAAAGGCGAAGAGGAT
AATATGGCAAGCCTGCCTGCAACACATGAACTGCATATTTTTGGTAGCATTAACG
GCGTGGATTTTGATATGGTTGGTCAAGGCACCGGTAATCCGAATGATGGTTATGA
AGAACTGAATCTGAAAAGCACCAAAGGCGATCTGCAGTTTAGCCCGTGGATTCTG
GTTCCGCATATTGGTTATGGTTTTCATCAGTATCTGCCGTATCCGGATGGTATGA
GCCCGTTTCAGGCAGCAATGGTTGATGGTAGCGGTTATCAGGTTCATCGTACCAT
GCAGTTTGAAGATGGTGCAAGCCTGACCGTTAATTATCGTTATACCTATGAAGGC
AGCCACATTAAAGGTGAAGCACAGGTTAAAGGTACAGGTTTTCCGGCAGATGGTC
CGGTTATGACCAATAGTCTGACCGCAGCAGATTGGTGTCGTAGCAAAAAAACCTA
TCCGAACGATAAAACCATCATCAGCACCTTCAAATGGTCATATACCACCGGCAAT
GGTAAACGTTATCGTAGCACCGCACGTACCACCTATACCTTTGCAAAACCGATGG
CAGCAAACTATCTGAAAAATCAGCCGATGTATGTGTTTCGCAAAACGGAACTGAA
ACATTCCAAAACCGAGCTGAACTTTAAAGAATGGCAGAAAGCATTTACCGATGTG
ATGGGTATGGATGAGCTGTACAAATAATGAggatcccgggaattctcgagtaaggttaacctgcaggagg
cctttaattaaggtggtgcggccgcgctagcggtcccgggggatcgatccggctgctaacaaagcccgaaaggaagctgagttggctgc
tgccaccgctgagcaataactagcataaccccttggggcctctaaacgggtcttgaggggttttttgctgaaaggaggaactatatc

0.2.4 pPURE-*mVenus* (Linear)

gaaattaatacgactcactatagggtctagaaataattttgtttaactttaagaaggaggtatacatATGGTGAGCAAGGG
CGAGGAGCTGTTCACCGGGGTGGTGCCCATCCTGGTCGAGCTGGACGGCGACGTA
AACGGCCACAAGTTCAGCGTGTCCGGCGAGGGCGAGGGCGATGCCACCTACGGCA
AGCTGACCCTGAAGCTCATCTGCACCACCGGCAAGCTGCCCGTGCCCTGGCCCAC
CCTCGTGACCACCCTCGGCTACGGCCTGCAGTGCTTCGCCCGCTACCCCGACCAC
ATGAAGCAGCACGACTTCTTCAAGTCCGCCATGCCCGAAGGCTACGTCCAGGAGC
GCACCATCTTCTTCAAGGACGACGGCAACTACAAGACCCGCGCCGAGGTGAAGTT
CGAGGGCGACACCCTGGTGAACCGCATCGAGCTGAAGGGCATCGACTTCAAGGAG
GACGGCAACATCCTGGGGCACAAGCTGGAGTACAACTACAACAGCCACAACGTCT
ATATCACCGCCGACAAGCAGAAGAACGGCATCAAGGCCAACTTCAAGATCCGCCA
CAACATCGAGGACGGCGGCGTGCAGCTCGCCGACCACTACCAGCAGAACACCCCC
ATCGGCGACGGCCCCGTGCTGCTGCCCGACAACCACTACCTGAGCTACCAGTCCA
AGCTGAGCAAAGACCCCAACGAGAAGCGCGATCACATGGTCCTGCTGGAGTTCGT
GACCGCCGCCGGGATCACTCTCGGCATGGACGAGCTGTACAAGTAATGAggatcccgg
gaattctcgagtaaggttaacctgcaggaggcctttaattaaggtggtgcggccgcgctagcggtcccgggggatcgatccggctgcta
acaaagcccgaaaggaagctgagttggctgctgccaccgctgagcaataactagcataaccccttggggcctctaaacgggtcttgag
gggttttttgctgaaaggaggaactatatc

0.2.5 pPURE-T7g10-*gp10(1-9)::mVenus* (Linear)

gaaattaatacgactcactatagggtctagggagaccacaacggtttccctctagaaataattttgtttaactttaagaaggagatatac
cATGGCTAGCATGACTGGTGGACAGCAACATATGGTGAGCAAGGGCGAGGAGCTG
TTCACCGGGGTGGTGCCCATCCTGGTCGAGCTGGACGGCGACGTAAACGGCCACA
AGTTCAGCGTGTCCGGCGAGGGCGAGGGCGATGCCACCTACGGCAAGCTGACCCT
GAAGCTCATCTGCACCACCGGCAAGCTGCCCGTGCCCTGGCCCACCCTCGTGACC
ACCCTCGGCTACGGCCTGCAGTGCTTCGCCCGCTACCCCGACCACATGAAGCAGC
ACGACTTCTTCAAGTCCGCCATGCCCGAAGGCTACGTCCAGGAGCGCACCATCTT
CTTCAAGGACGACGGCAACTACAAGACCCGCGCCGAGGTGAAGTTCGAGGGCGAC
ACCCTGGTGAACCGCATCGAGCTGAAGGGCATCGACTTCAAGGAGGACGGCAACA
TCCTGGGGCACAAGCTGGAGTACAACTACAACAGCCACAACGTCTATATCACCGC
CGACAAGCAGAAGAACGGCATCAAGGCCAACTTCAAGATCCGCCACAACATCGAG
GACGGCGGCGTGCAGCTCGCCGACCACTACCAGCAGAACACCCCCATCGGCGACG
GCCCCGTGCTGCTGCCCGACAACCACTACCTGAGCTACCAGTCCAAGCTGAGCAA
AGACCCCAACGAGAAGCGCGATCACATGGTCCTGCTGGAGTTCGTGACCGCCGCC
GGGATCACTCTCGGCATGGACGAGCTGTACAAGTAATGAggatcccgggaattctcgagtaaggt
taacctgcaggaggcctttaattaaggtggtgcggccgcgctagcggtcccgggggatcgatccggctgctaacaaagcccgaaagga
agctgagttggctgctgccaccgctgagcaataactagcataaccccttggggcctctaaacgggtcttgaggggttttttgctgaaagg
aggaactatatc

0.2.6 pPURE-*bjaI* (Linear)

gaaattaatacgactcactatagggtctagaaataattttgtttaactttaagaaggaggtatacatATGATTCACGCAAT
TTCCGCGGTCAATCGCCACTTATACGAGGACGTACTCGAGCAGCATTTCCGGCTG
CGTCATGACATCTTTGTCGAGGAGCGGCACTGGGAGACGCTGCGCAGGCCGGATG
GCCGCGAGGTCGATTCCTATGACGACGAGGATACCGTCTATCTGCTTGCGCTGGA
GGGACGGCGCGTCGTCGGCGGCCACCGGCTCTACCCCACGACCAAGCCCTCGATG
ATGAGCGAGGTCTTCCCGCATCTGGCGGCGGTTCGCGGCTGCCCCTCGGATCCGC
TGATCTGGGAATGGTCGCGCTACTTCGTCGTCCGCGATCGCCGCGACGGCGCGCT
CAACCTGCAACTGATGGCGGCGGTGCAGGAGTTCTGCCTCGACCAGGGAATCGCG
CAGGTCAGCGCGATCATGGAAACCTGGTGGTTGCCGCGCTTCCACGAGGCCGGCT
TCGTCGTGACGCCGCTCGGCCTGCCGGCTCTGGTCGAGAACGCCTGGACCATGGC
GGCCACCGTCGACATTCGTCGCCAGACGCTCGATGTCCTGCATGATCGCATCGGC
ATGCCTTCCATCGTGCAACAGGACGGCCCGCGTCTGGACGCCGTCGCCCGTGCCA
ACCTTTGCGGCCTCGCTGCCGCGCAACGAAAGAGCGCCTGATGAggatcccgggaattctcg
agtaaggttaacctgcaggaggcctttaattaaggtggtgcggccgcgctagcggtcccgggggatcgatccggctgctaacaaagccc
gaaaggaagctgagttggctgctgccaccgctgagcaataactagcataaccccttggggcctctaaacgggtcttgaggggttttttg
ctgaaaggaggaactatatc

0.2.7 pSB1A3-*bjaR-gfp*

gcagaatttcagataaaaaaaatccttagctttcgctaaggatgatttctggaattcgcggccgcttctagagtactgggaaatttccca
atattacgcaagaaaatggtttgttatagtcgaatattactagagtcacacaggaaagtactagATGCGTAAAGGAGAA
GAACTTTTCACTGGAGTTGTCCCAATTCTTGTTGAATTAGATGGTGATGTTAATG
GGCACAAATTTTCTGTCAGTGGAGAGGGTGAAGGTGATGCAACATACGGAAAACT
TACCCTTAAATTTATTTGCACTACTGGAAAACTACCTGTTCCATGGCCAACACTTG
TCACTACTTTCGGTTATGGTGTTCAATGCTTTGCGAGATACCCAGATCATATGAA
ACAGCATGACTTTTTCAAGAGTGCCATGCCCGAAGGTTATGTACAGGAAAGAACT
ATATTTTTCAAAGATGACGGGAACTACAAGACACGTGCTGAAGTCAAGTTTGAAG
GTGATACCCTTGTTAATAGAATCGAGTTAAAAGGTATTGATTTTAAAGAAGATGG
AAACATTCTTGGACACAAATTGGAATACAACTATAACTCACACAATGTATACATC
ATGGCAGACAAACAAAAGAATGGAATCAAAGTTAACTTCAAAATTAGACACAACA
TTGAAGATGGAAGCGTTCAACTAGCAGACCATTATCAACAAAATACTCCAATTGG
CGATGGCCCTGTCCTTTTACCAGACAACCATTACCTGTCCACACAATCTGCCCTTT
CGAAAGATCCCAACGAAAAGAGAGACCACATGGTCCTTCTTGAGTTTGTAACAGC
TGCTGGGATTACACATGGCATGGATGAACTATACAAATAATAAtactagagccggcttatcgg
tcagtttcacctgatttacgtaaaaacccgcttcggcgggtttttgcttttggaggggcagaaagatgaatgactgtccacgacgctata
cccaaaagaaatactagagttgacggctagctcagtcctaggtacagtgctagctactagagaaagaggagaaatactagATGTC
CGCAGTAGATTATGGGCGTGAAGCCCTGGACTTTATCGAGGGTTTGGGCGTATAT
CGTAAAGTCCCTGATGCGATGAACGCTCTCGAAGCGGCATTCGGTCGCTTTGGCT
TTGAGACAATCATCGTGACTGGGCTGCCCAACCCGGATCAGCGTTTTGCACAAAT
GGTACTCGCTAAACGTTGGCCGGCGGGTTGGTTTAATCTGTATACGCAAAACAAT
TATGACCGTTTTGATCCCGTCGTCCGCTTGTGCCGTCAGAGCGTGAATCCGTTCG
AGTGGAGTGAAGCACCGTATGACGCTGAATTAGAACCAAGCGCCGCCGAAGTCAT
GAATCGTGCAGGCGACTTCCGTATGTCCCGTGGTTTTATCGTGCCGATCCATGGA
CTGACAGGATATGAAGCGGCTGTTAGTCTGGGAGGCGTTCATTTAGATCTGAACC
CGCGTTCCAAACCGGCTCTGCACCTGATGGCAATGTACGGTTTCGACCACATTCG
TCGCCTGCTGGAACCGACCCCATATCCTTCGACGCGCCTTACTCCGCGCGAACGC
GAAGTTATTTCCTGGGCTAGCCAGGGAAAATCTGCGTGGGAAATTGGTGAAATCC
TGCATATCACCCAGCGCACCGCCGAGGAGCACTTAGCGACCGCGGCACGTAAACT
TGGTGCGGTCAATCGTACCCATGCCGTCGCGCTGGCCATCCGTCACAAAATTATC
AACCCCTAAtactagagccaggcatcaaataaaacgaaaggctcagtcgaaagactgggcctttcgttttatctgttgtttgtcg
gtgaacgctctctactagagtcacactggctcaccttcgggtgggcctttctgcgtttatatactagtagcggccgctgcagtccggcaa
aaaagggcaaggtgtcaccaccctgccctttttctttaaaaccgaaaagattacttcgcgttatgcaggcttcctcgctcactgactcgct
gcgctcggtcgttcggctgcggcgagcggtatcagctcactcaaaggcggtaatacggttatccacagaatcaggggataacgcagga
aagaacatgtgagcaaaaggccagcaaaaggccaggaaccgtaaaaaggccgcgttgctggcgtttttccacaggctccgcccccctg
acgagcatcacaaaaatcgacgctcaagtcagaggtggcgaaacccgacaggactataaagataccaggcgtttccccctggaagctc
cctcgtgcgctctcctgttccgaccctgccgcttaccggatacctgtccgcctttctcccttcgggaagcgtggcgctttctcatagctcac
gctgtaggtatctcagttcggtgtaggtcgttcgctccaagctgggctgtgtgcacgaaccccccgttcagcccgaccgctgcgccttatc
cggtaactatcgtcttgagtccaacccggtaagacacgacttatcgccactggcagcagccactggtaacaggattagcagagcgaggt
atgtaggcggtgctacagagttcttgaagtggtggcctaactacggctacactagaagaacagtatttggtatctgcgctctgctgaagc
cagttaccttcggaaaaagagttggtagctcttgatccggcaaacaaaccaccgctggtagcggtggtttttttgtttgcaagcagcaga

ttacgcgcagaaaaaaaggatctcaagaagatcctttgatcttttctacggggtctgacgctcagtggaacgaaaactcacgttaaggg
attttggtcatgagattatcaaaaaggatcttcacctagatccttttaaattaaaaatgaagttttaaatcaatctaaagtatatatgagt
aaacttggtctgacagttaccaatgcttaatcagtgaggcacctatctcagcgatctgtctatttcgttcatccatagttgcctgactcccc
gtcgtgtagataactacgatacgggagggcttaccatctggccccagtgctgcaatgataccgcgagacccacgctcaccggctccaga
tttatcagcaataaaccagccagccggaagggccgagcgcagaagtggtcctgcaactttatccgcctccatccagtctattaattgttg
ccgggaagctagagtaagtagttcgccagttaatagtttgcgcaacgttgttgccattgctacaggcatcgtggtgtcacgctcgtcgtt
tggtatggcttcattcagctccggttcccaacgatcaaggcgagttacatgatcccccatgttgtgcaaaaaagcggttagctccttcgg
tcctccgatcgttgtcagaagtaagttggccgcagtgttatcactcatggttatggcagcactgcataattctcttactgtcatgccatcc
gtaagatgcttttctgtgactggtgagtactcaaccaagtcattctgagaatagtgtatgcggcgaccgagttgctcttgcccggcgtca
atacgggataataccgcgccacatagcagaactttaaaagtgctcatcattggaaaacgttcttcggggcgaaaactctcaaggatctt
accgctgttgagatccagttcgatataacccactcgtgcacccaactgatcttcagcatcttttactttcaccagcgtttctgggtgagca
aaaacaggaaggcaaaatgccgcaaaaaagggaataagggcgacacggaaatgttgaatactcatactcttcctttttcaatattattg
aagcatttatcagggttattgtctcatgagcggatacatatttgaatgtatttagaaaaataaacaaataggggttccgcgcacatttcc
ccgaaaagtgccacctgacgtctaagaaaccattattatcatgacattaacctataaaaataggcgtatcacgag

0.2.8 pSB1A3-$bjaR_{KO}$-gfp

gcagaatttcagataaaaaaaatccttagctttcgctaaggatgatttctggaattcgcggccgcttctagagtactgggaaatttccca
atattacgcaagaaaatggtttgttatagtcgaatattactagagtcacacaggaaagtactagATGCGTAAAGGAGAA
GAACTTTTCACTGGAGTTGTCCCAATTCTTGTTGAATTAGATGGTGATGTTAATG
GGCACAAATTTTCTGTCAGTGGAGAGGGTGAAGGTGATGCAACATACGGAAAACT
TACCCTTAAATTTATTTGCACTACTGGAAAACTACCTGTTCCATGGCCAACACTTG
TCACTACTTTCGGTTATGGTGTTCAATGCTTTGCGAGATACCCAGATCATATGAA
ACAGCATGACTTTTTCAAGAGTGCCATGCCCGAAGGTTATGTACAGGAAAGAACT
ATATTTTTCAAAGATGACGGGAACTACAAGACACGTGCTGAAGTCAAGTTTGAAG
GTGATACCCTTGTTAATAGAATCGAGTTAAAAGGTATTGATTTTAAAGAAGATGG
AAACATTCTTGGACACAAATTGGAATACAACTATAACTCACACAATGTATACATC
ATGGCAGACAAACAAAAGAATGGAATCAAAGTTAACTTCAAAATTAGACACAACA
TTGAAGATGGAAGCGTTCAACTAGCAGACCATTATCAACAAAATACTCCAATTGG
CGATGGCCCTGTCCTTTTACCAGACAACCATTACCTGTCCACACAATCTGCCCTTT
CGAAAGATCCCAACGAAAAGAGAGACCACATGGTCCTTCTTGAGTTTGTAACAGC
TGCTGGGATTAcacatggcatggatgaactatacaaataataatactagagccggcttatcggtcagtttcacctgatttacg
taaaaacccgcttcggcgggtttttgcttttggaggggcagaaagatgaatgactgtccacgacgctatacccaaaagaaatactagag
ttgacggctagctcagtcctaggtacagtgctagctactagagaaagaggagaaatactagATGTCCGCAGTAGATTA
TGGGCGTGAAGCCCTGTGACATTCTCTCAAAGTATTATGCAGGGCCATCCGTCAC
AAAATTATCAACCCCTAAtactagagccaggcatcaaataaaacgaaaggctcagtcgaaagactgggcctttcgttt
tatctgttgtttgtcggtgaacgctctctactagagtcacactggctcaccttcgggtgggcctttctgcgtttatatactagtagcggccg
ctgcagtccggcaaaaaagggcaaggtgtcaccaccctgccctttttctttaaaaccgaaaagattacttcgcgttatgcaggcttcctc
gctcactgactcgctgcgctcggtcgttcggctgcggcgagcggtatcagctcactcaaaggcggtaatacggttatccacagaatcag
gggataacgcaggaaagaacatgtgagcaaaaggccagcaaaaggccaggaaccgtaaaaaggccgcgttgctggcgtttttccaca
ggctccgcccccctgacgagcatcacaaaaatcgacgctcaagtcagaggtggcgaaacccgacaggactataaagataccaggcgtt
tccccctggaagctccctcgtgcgctctcctgttccgaccctgccgcttaccggatacctgtccgcctttctcccttcgggaagcgtggcgc

tttctcatagctcacgctgtaggtatctcagttcggtgtaggtcgttcgctccaagctgggctgtgtgcacgaaccccccgttcagcccga
ccgctgcgccttatccggtaactatcgtcttgagtccaacccggtaagacacgacttatcgccactggcagcagccactggtaacaggat
tagcagagcgaggtatgtaggcggtgctacagagttcttgaagtggtggcctaactacggctacactagaagaacagtatttggtatct
gcgctctgctgaagccagttaccttcggaaaaagagttggtagctcttgatccggcaaacaaaccaccgctggtagcggtggtttttttg
tttgcaagcagcagattacgcgcagaaaaaaaggatctcaagaagatcctttgatcttttctacggggtctgacgctcagtggaacgaa
aactcacgttaagggattttggtcatgagattatcaaaaaggatcttcacctagatccttttaaattaaaaatgaagttttaaatcaatct
aaagtatatatgagtaaacttggtctgacagttaccaatgcttaatcagtgaggcacctatctcagcgatctgtctatttcgttcatccat
agttgcctgactccccgtcgtgtagataactacgatacgggagggcttaccatctggccccagtgctgcaatgataccgcgagacccac
gctcaccggctccagatttatcagcaataaaccagccagccggaagggccgagcgcagaagtggtcctgcaactttatccgcctccatc
cagtctattaattgttgccgggaagctagagtaagtagttcgccagttaatagtttgcgcaacgttgttgccattgctacaggcatcgtg
gtgtcacgctcgtcgtttggtatggcttcattcagctccggttcccaacgatcaaggcgagttacatgatcccccatgttgtgcaaaaaa
gcggttagctccttcggtcctccgatcgttgtcagaagtaagttggccgcagtgttatcactcatggttatggcagcactgcataattctc
ttactgtcatgccatccgtaagatgcttttctgtgactggtgagtactcaaccaagtcattctgagaatagtgtatgcggcgaccgagttg
ctcttgcccggcgtcaatacgggataataccgcgccacatagcagaactttaaaagtgctcatcattggaaaacgttcttcggggcgaa
aactctcaaggatcttaccgctgttgagatccagttcgatataacccactcgtgcacccaactgatcttcagcatcttttactttcaccag
cgtttctgggtgagcaaaaacaggaaggcaaaatgccgcaaaaaagggaataagggcgacacggaaatgttgaatactcatactcttc
ctttttcaatattattgaagcatttatcagggttattgtctcatgagcggatacatatttgaatgtatttagaaaaataaacaaataggggttccgcgcacatttccccgaaaagtgccacctgacgtctaagaaaccattattatcatgacattaacctataaaaataggcgtatcacga
g

0.2.9 pSB1A3-*bjaR*-*gfp*-*kanR*

gcagaatttcagataaaaaaaaatccttagctttcgctaaggatgatttctggaattcgcggccgcttctagagtactgggaaatttccca
atattacgcaagaaaatggtttgttatagtcgaatattactagagtcacacaggaaagtactagATGCGTAAAGGAGAA
GAACTTTTCACTGGAGTTGTCCCAATTCTTGTTGAATTAGATGGTGATGTTAATG
GGCACAAATTTTCTGTCAGTGGAGAGGGTGAAGGTGATGCAACATACGGAAAACT
TACCCTTAAATTTATTTGCACTACTGGAAAACTACCTGTTCCATGGCCAACACTTG
TCACTACTTTCGGTTATGGTGTTCAATGCTTTGCGAGATACCCAGATCATATGAA
ACAGCATGACTTTTTCAAGAGTGCCATGCCCGAAGGTTATGTACAGGAAAGAACT
ATATTTTTCAAAGATGACGGGAACTACAAGACACGTGCTGAAGTCAAGTTTGAAG
GTGATACCCTTGTTAATAGAATCGAGTTAAAAGGTATTGATTTTAAAGAAGATGG
AAACATTCTTGGACACAAATTGGAATACAACTATAACTCACACAATGTATACATC
ATGGCAGACAAACAAAAGAATGGAATCAAAGTTAACTTCAAAATTAGACACAACA
TTGAAGATGGAAGCGTTCAACTAGCAGACCATTATCAACAAAATACTCCAATTGG
CGATGGCCCTGTCCTTTTACCAGACAACCATTACCTGTCCACACAATCTGCCCTTT
CGAAAGATCCCAACGAAAAGAGAGACCACATGGTCCTTCTTGAGTTTGTAACAGC
TGCTGGGATTACACATGGCATGGATGAACTATACAAATAATAAagtaaatctaagcaggtcc
gcATGAGCCATATTCAACGGGAAACGTCTTGCTCTAGGCCGCGATTAAATTCCAAC
ATGGATGCTGATTTATATGGGTATAAATGGGCTCGCGATAATGTCGGGCAATCAG
GTGCGACAATCTATCGATTGTATGGGAAGCCCGATGCGCCAGAGTTGTTTCTGAA
ACATGGCAAAGGTAGCGTTGCCAATGATGTTACAGATGAGATGGTCAGACTAAAC
TGGCTGACGGAATTTATGCCTCTTCCGACCATCAAGCATTTTATCCGTACTCCTG

ATGATGCATGGTTACTCACCACTGCGATCCCCGGGAAAACAGCATTCCAGGTATT
AGAAGAATATCCTGATTCAGGTGAAAATATTGTTGATGCGCTGGCAGTGTTCCTG
CGCCGGTTGCATTCGATTCCTGTTTGTAATTGTCCTTTTAACAGCGATCGCGTAT
TTCGTCTCGCTCAGGCGCAATCACGAATGAATAACGGTTTGGTTGATGCGAGTGA
TTTTGATGACGAGCGTAATGGCTGGCCTGTTGAACAAGTCTGGAAAGAAATGCAT
AAACTTTTGCCATTCTCACCGGATTCAGTCGTCACTCATGGTGATTTCTCACTTGA
TAACCTTATTTTTGACGAGGGGAAATTAATAGGTTGTATTGATGTTGGACGAGTC
GGAATCGCAGACCGATACCAGGATCTTGCCATCCTATGGAACTGCCTCGGTGAGT
TTTCTCCTTCATTACAGAAACGGCTTTTTCAAAAATATGGTATTGATAATCCTGAT
ATGAATAAATTGCAGTTTCATTTGATGCTCGATGAGTTTTTCTAATAAtactagagccgg
cttatcggtcagtttcacctgatttacgtaaaaacccgcttcggcgggtttttgcttttggaggggcagaaagatgaatgactgtccacg
acgctatacccaaaagaaatactagagttgacggctagctcagtcctaggtacagtgctagctactagagaaagaggagaaatactag
ATGTCCGCAGTAGATTATGGGCGTGAAGCCCTGGACTTTATCGAGGGTTTGGGCG
TATATCGTAAAGTCCCTGATGCGATGAACGCTCTCGAAGCGGCATTCGGTCGCTT
TGGCTTTGAGACAATCATCGTGACTGGGCTGCCCAACCCGGATCAGCGTTTTGCA
CAAATGGTACTCGCTAAACGTTGGCCGGCGGGTTGGTTTAATCTGTATACGCAAA
ACAATTATGACCGTTTTGATCCCGTCGTCCGCTTGTGCCGTCAGAGCGTGAATCC
GTTCGAGTGGAGTGAAGCACCGTATGACGCTGAATTAGAACCAAGCGCCGCCGAA
GTCATGAATCGTGCAGGCGACTTCCGTATGTCCCGTGGTTTTATCGTGCCGATCC
ATGGACTGACAGGATATGAAGCGGCTGTTAGTCTGGGAGGCGTTCATTTAGATCT
GAACCCGCGTTCCAAACCGGCTCTGCACCTGATGGCAATGTACGGTTTCGACCAC
ATTCGTCGCCTGCTGGAACCGACCCCATATCCTTCGACGCGCCTTACTCCGCGCG
AACGCGAAGTTATTTCCTGGGCTAGCCAGGGAAAATCTGCGTGGGAAATTGGTGA
AATCCTGCATATCACCCAGCGCACCGCCGAGGAGCACTTAGCGACCGCGGCACGT
AAACTTGGTGCGGTCAATCGTACCCATGCCGTCGCGCTGGCCATCCGTCACAAAA
TTATCAACCCCTAAtactagagccaggcatcaaataaaacgaaaggctcagtcgaaagactgggcctttcgttttatctgt
tgtttgtcggtgaacgctctctactagagtcacactggctcaccttcgggtgggcctttctgcgtttatatactagtagcggccgctgcag
tccggcaaaaaagggcaaggtgtcaccaccctgccctttttctttaaaaccgaaaagattacttcgcgttatgcaggcttcctcgctcac
tgactcgctgcgctcggtcgttcggctgcggcgagcggtatcagctcactcaaaggcggtaatacggttatccacagaatcaggggata
acgcaggaaagaacatgtgagcaaaaggccagcaaaaggccaggaaccgtaaaaaggccgcgttgctggcgtttttccacaggctcc
gcccccctgacgagcatcacaaaaatcgacgctcaagtcagaggtggcgaaacccgacaggactataaagataccaggcgtttcccc
tggaagctccctcgtgcgctctcctgttccgaccctgccgcttaccggatacctgtccgcctttctcccttcgggaagcgtggcgctttctc
atagctcacgctgtaggtatctcagttcggtgtaggtcgttcgctccaagctgggctgtgtgcacgaaccccccgttcagcccgaccgct
gcgccttatccggtaactatcgtcttgagtccaacccggtaagacacgacttatcgccactggcagcagccactggtaacaggattagc
agagcgaggtatgtaggcggtgctacagagttcttgaagtggtggcctaactacggctacactagaagaacagtatttggtatctgcgc
tctgctgaagccagttaccttcggaaaaagagttggtagctcttgatccggcaaacaaaccaccgctggtagcggtggtttttttgtttg
caagcagcagattacgcgcagaaaaaaaggatctcaagaagatcctttgatcttttctacggggtctgacgctcagtggaacgaaaac
tcacgttaagggattttggtcatgagattatcaaaaaggatcttcacctagatccttttaaattaaaaatgaagttttaaatcaatctaa
agtatatatgagtaaacttggtctgacagttaccaatgcttaatcagtgaggcacctatctcagcgatctgtctatttcgttcatccatag
ttgcctgactccccgtcgtgtagataactacgatacgggagggcttaccatctggccccagtgctgcaatgataccgcgagacccacgct
caccggctccagatttatcagcaataaaccagccagccggaagggccgagcgcagaagtggtcctgcaactttatccgcctccatccag
tctattaattgttgccgggaagctagagtaagtagttcgccagttaatagtttgcgcaacgttgttgccattgctacaggcatcgtggtgt
cacgctcgtcgtttggtatggcttcattcagctccggttcccaacgatcaaggcgagttacatgatcccccatgttgtgcaaaaaagcgg

ttagctccttcggtcctccgatcgttgtcagaagtaagttggccgcagtgttatcactcatggttatggcagcactgcataattctcttact
gtcatgccatccgtaagatgcttttctgtgactggtgagtactcaaccaagtcattctgagaatagtgtatgcggcgaccgagttgctctt
gcccggcgtcaatacgggataataccgcgccacatagcagaactttaaaagtgctcatcattggaaaacgttcttcggggcgaaaactc
tcaaggatcttaccgctgttgagatccagttcgatataacccactcgtgcacccaactgatcttcagcatcttttactttcaccagcgtttc
tgggtgagcaaaaacaggaaggcaaaatgccgcaaaaaagggaataagggcgacacggaaatgttgaatactcatactcttcctttt t
caatattattgaagcatttatcagggttattgtctcatgagcggatacatatttgaatgtatttagaaaaataaacaaataggggttccg
cgcacatttccccgaaaagtgccacctgacgtctaagaaaccattattatcatgacattaacctataaaaataggcgtatcacgag

0.2.10 pSB1A3-*bjaR*$_{KO}$-*gfp*-*kanR*

gcagaatttcagataaaaaaaatccttagctttcgctaaggatgatttctggaattcgcggccgcttctagagtactgggaaatttccca
atattacgcaagaaaatggtttgttatagtcgaatattactagagtcacacaggaaagtactagATGCGTAAAGGAGAA
GAACTTTTCACTGGAGTTGTCCCAATTCTTGTTGAATTAGATGGTGATGTTAATG
GGCACAAATTTTCTGTCAGTGGAGAGGGTGAAGGTGATGCAACATACGGAAAACT
TACCCTTAAATTTATTTGCACTACTGGAAAACTACCTGTTCCATGGCCAACACTTG
TCACTACTTTCGGTTATGGTGTTCAATGCTTTGCGAGATACCCAGATCATATGAA
ACAGCATGACTTTTTCAAGAGTGCCATGCCCGAAGGTTATGTACAGGAAAGAACT
ATATTTTTCAAAGATGACGGGAACTACAAGACACGTGCTGAAGTCAAGTTTGAAG
GTGATACCCTTGTTAATAGAATCGAGTTAAAAGGTATTGATTTTAAAGAAGATGG
AAACATTCTTGGACACAAATTGGAATACAACTATAACTCACACAATGTATACATC
ATGGCAGACAAACAAAAGAATGGAATCAAAGTTAACTTCAAAATTAGACACAACA
TTGAAGATGGAAGCGTTCAACTAGCAGACCATTATCAACAAAATACTCCAATTGG
CGATGGCCCTGTCCTTTTACCAGACAACCATTACCTGTCCACACAATCTGCCCTTT
CGAAAGATCCCAACGAAAAGAGAGACCACATGGTCCTTCTTGAGTTTGTAACAGC
TGCTGGGATTACACATGGCATGGATGAACTATACAAATAATAAagtaaatctaagcaggtcc
gcATGAGCCATATTCAACGGGAAACGTCTTGCTCTAGGCCGCGATTAAATTCCAAC
ATGGATGCTGATTTATATGGGTATAAATGGGCTCGCGATAATGTCGGGCAATCAG
GTGCGACAATCTATCGATTGTATGGGAAGCCCGATGCGCCAGAGTTGTTTCTGAA
ACATGGCAAAGGTAGCGTTGCCAATGATGTTACAGATGAGATGGTCAGACTAAAC
TGGCTGACGGAATTTATGCCTCTTCCGACCATCAAGCATTTTATCCGTACTCCTG
ATGATGCATGGTTACTCACCACTGCGATCCCCGGGAAAACAGCATTCCAGGTATT
AGAAGAATATCCTGATTCAGGTGAAAATATTGTTGATGCGCTGGCAGTGTTCCTG
CGCCGGTTGCATTCGATTCCTGTTTGTAATTGTCCTTTTAACAGCGATCGCGTAT
TTCGTCTCGCTCAGGCGCAATCACGAATGAATAACGGTTTGGTTGATGCGAGTGA
TTTTGATGACGAGCGTAATGGCTGGCCTGTTGAACAAGTCTGGAAAGAAATGCAT
AAACTTTTGCCATTCTCACCGGATTCAGTCGTCACTCATGGTGATTTCTCACTTGA
TAACCTTATTTTTGACGAGGGGAAATTAATAGGTTGTATTGATGTTGGACGAGTC
GGAATCGCAGACCGATACCAGGATCTTGCCATCCTATGGAACTGCCTCGGTGAGT
TTTCTCCTTCATTACAGAAACGGCTTTTTCAAAAATATGGTATTGATAATCCTGAT
ATGAATAAATTGCAGTTTCATTTGATGCTCGATGAGTTTTTCTAATAAtactagagccgg
cttatcggtcagtttcacctgatttacgtaaaaacccgcttcggcgggtttttgcttttggaggggcagaaagatgaatgactgtccacg
acgctatacccaaaagaaatactagagttgacggctagctcagtcctaggtacagtgctagctactagagaaagaggagaaatactag

ATGTCCGCAGTAGATTATGGGCGTGAAGCCCTGTGACATTCTCTCAAAGTATTAT
GCAGGGCCATCCGTCACAAAATTATCAACCCCTAAtactagagccaggcatcaaataaaacgaaagg
ctcagtcgaaagactgggcctttcgttttatctgttgtttgtcggtgaacgctctctactagagtcacactggctcaccttcgggtgggcct
ttctgcgtttatatactagtagcggccgctgcagtccggcaaaaaagggcaaggtgtcaccaccctgcccttttctttaaaaccgaaaa
gattacttcgcgttatgcaggcttcctcgctcactgactcgctgcgctcggtcgttcggctgcggcgagcggtatcagctcactcaaaggc
ggtaatacggttatccacagaatcaggggataacgcaggaaagaacatgtgagcaaaaggccagcaaaaggccaggaaccgtaaaa
aggccgcgttgctggcgtttttccacaggctccgcccccctgacgagcatcacaaaaatcgacgctcaagtcagaggtggcgaaacccg
acaggactataaagataccaggcgtttccccctggaagctccctcgtgcgctctcctgttccgaccctgccgcttaccggatacctgtccg
cctttctcccttcgggaagcgtggcgctttctcatagctcacgctgtaggtatctcagttcggtgtaggtcgttcgctccaagctgggctgt
gtgcacgaaccccccgttcagcccgaccgctgcgccttatccggtaactatcgtcttgagtccaacccggtaagacacgacttatcgcca
ctggcagcagccactggtaacaggattagcagagcgaggtatgtaggcggtgctacagagttcttgaagtggtggcctaactacggct
acactagaagaacagtatttggtatctgcgctctgctgaagccagttaccttcggaaaaagagttggtagctcttgatccggcaaacaa
accaccgctggtagcggtggtttttttgtttgcaagcagcagattacgcgcagaaaaaaaggatctcaagaagatcctttgatcttttct
acggggtctgacgctcagtggaacgaaaactcacgttaagggattttggtcatgagattatcaaaaaggatcttcacctagatcctttta
aattaaaaatgaagttttaaatcaatctaaagtatatatgagtaaacttggtctgacagttaccaatgcttaatcagtgaggcacctatc
tcagcgatctgtctatttcgttcatccatagttgcctgactccccgtcgtgtagataactacgatacgggagggcttaccatctggcccca
gtgctgcaatgataccgcgagacccacgctcaccggctccagatttatcagcaataaaccagccagccggaagggccgagcgcagaag
tggtcctgcaactttatccgcctccatccagtctattaattgttgccgggaagctagagtaagtagttcgccagttaatagtttgcgcaac
gttgttgccattgctacaggcatcgtggtgtcacgctcgtcgtttggtatggcttcattcagctccggttcccaacgatcaaggcgagtta
catgatcccccatgttgtgcaaaaaagcggttagctccttcggtcctccgatcgttgtcagaagtaagttggccgcagtgttatcactcat
ggttatggcagcactgcataattctcttactgtcatgccatccgtaagatgcttttctgtgactggtgagtactcaaccaagtcattctga
gaatagtgtatgcggcgaccgagttgctcttgcccggcgtcaatacgggataataccgcgccacatagcagaactttaaaagtgctcat
cattggaaaacgttcttcggggcgaaaactctcaaggatcttaccgctgttgagatccagttcgatataacccactcgtgcacccaactg
atcttcagcatcttttactttcaccagcgtttctgggtgagcaaaaacaggaaggcaaaatgccgcaaaaaagggaataagggcgaca
cggaaatgttgaatactcatactcttcctttttcaatattattgaagcatttatcagggttattgtctcatgagcggatacatatttgaatg
tatttagaaaaataaacaaataggggttccgcgcacatttccccgaaaagtgccacctgacgtctaagaaaccattattatcatgacatt
aacctataaaaataggcgtatcacgag

0.2.11 pSB1A3-*bjaR-gfp* (B0031 | ATG)

gcagaatttcagataaaaaaaaatccttagctttcgctaaggatgatttctggaattcgcggccgcttctagagtactgggaaatttccca
atattacgcaagaaaatggtttgttatagtcgaatattactagagtcacacaggaaagtactagATGCGTAAAGGAGAA
GAACTTTTCACTGGAGTTGTCCCAATTCTTGTTGAATTAGATGGTGATGTTAATG
GGCACAAATTTTCTGTCAGTGGAGAGGGTGAAGGTGATGCAACATACGGAAAACT
TACCCTTAAATTTATTTGCACTACTGGAAAACTACCTGTTCCATGGCCAACACTTG
TCACTACTTTCGGTTATGGTGTTCAATGCTTTGCGAGATACCCAGATCATATGAA
ACAGCATGACTTTTTCAAGAGTGCCATGCCCGAAGGTTATGTACAGGAAAGAACT
ATATTTTTCAAAGATGACGGGAACTACAAGACACGTGCTGAAGTCAAGTTTGAAG
GTGATACCCTTGTTAATAGAATCGAGTTAAAAGGTATTGATTTTAAAGAAGATGG
AAACATTCTTGGACACAAATTGGAATACAACTATAACTCACACAATGTATACATC
ATGGCAGACAAACAAAAGAATGGAATCAAAGTTAACTTCAAAATTAGACACAACA
TTGAAGATGGAAGCGTTCAACTAGCAGACCATTATCAACAAAATACTCCAATTGG

CGATGGCCCTGTCCTTTTACCAGACAACCATTACCTGTCCACACAATCTGCCCTTT
CGAAAGATCCCAACGAAAAGAGAGACCACATGGTCCTTCTTGAGTTTGTAACAGC
TGCTGGGATTACACATGGCATGGATGAACTATACAAATAATAAtactagagccggcttatcgg
tcagtttcacctgatttacgtaaaaacccgcttcggcgggtttttgcttttggaggggcagaaagatgaatgactgtccacgacgctata
cccaaaagaaatactagagttgacggctagctcagtcctaggtacagtgctagctactagagtcacacaggaaacctactagATGT
CCGCAGTAGATTATGGGCGTGAAGCCCTGGACTTTATCGAGGGTTTGGGCGTATA
TCGTAAAGTCCCTGATGCGATGAACGCTCTCGAAGCGGCATTCGGTCGCTTTGGC
TTTGAGACAATCATCGTGACTGGGCTGCCCAACCCGGATCAGCGTTTTGCACAAA
TGGTACTCGCTAAACGTTGGCCGGCGGGTTGGTTTAATCTGTATACGCAAAACAA
TTATGACCGTTTTGATCCCGTCGTCCGCTTGTGCCGTCAGAGCGTGAATCCGTTC
GAGTGGAGTGAAGCACCGTATGACGCTGAATTAGAACCAAGCGCCGCCGAAGTCA
TGAATCGTGCAGGCGACTTCCGTATGTCCCGTGGTTTTATCGTGCCGATCCATGG
ACTGACAGGATATGAAGCGGCTGTTAGTCTGGGAGGCGTTCATTTAGATCTGAAC
CCGCGTTCCAAACCGGCTCTGCACCTGATGGCAATGTACGGTTTCGACCACATTC
GTCGCCTGCTGGAACCGACCCCATATCCTTCGACGCGCCTTACTCCGCGCGAACG
CGAAGTTATTTCCTGGGCTAGCCAGGGAAAATCTGCGTGGGAAATTGGTGAAATC
CTGCATATCACCCAGCGCACCGCCGAGGAGCACTTAGCGACCGCGGCACGTAAAC
TTGGTGCGGTCAATCGTACCCATGCCGTCGCGCTGGCCATCCGTCACAAAATTAT
CAACCCCTAAtactagagccaggcatcaaataaaacgaaaggctcagtcgaaagactgggcctttcgttttatctgttgtttgt
cggtgaacgctctctactagagtcacactggctcaccttcgggtgggcctttctgcgtttatatactagtagcggccgctgcagtccggca
aaaaagggcaaggtgtcaccaccctgccctttttctttaaaaccgaaaagattacttcgcgttatgcaggcttcctcgctcactgactcgc
tgcgctcggtcgttcggctgcggcgagcggtatcagctcactcaaaggcggtaatacggttatccacagaatcaggggataacgcagg
aaagaacatgtgagcaaaaggccagcaaaaggccaggaaccgtaaaaaggccgcgttgctggcgtttttccacaggctccgcccccct
gacgagcatcacaaaaatcgacgctcaagtcagaggtggcgaaacccgacaggactataaagataccaggcgtttccccctggaagct
ccctcgtgcgctctcctgttccgaccctgccgcttaccggatacctgtccgcctttctcccttcgggaagcgtggcgctttctcatagctca
cgctgtaggtatctcagttcggtgtaggtcgttcgctccaagctgggctgtgtgcacgaaccccccgttcagcccgaccgctgcgccttat
ccggtaactatcgtcttgagtccaacccggtaagacacgacttatcgccactggcagcagccactggtaacaggattagcagagcgagg
tatgtaggcggtgctacagagttcttgaagtggtggcctaactacggctacactagaagaacagtatttggtatctgcgctctgctgaag
ccagttaccttcggaaaaagagttggtagctcttgatccggcaaacaaaccaccgctggtagcggtggtttttttgtttgcaagcagcag
attacgcgcagaaaaaaaggatctcaagaagatcctttgatcttttctacggggtctgacgctcagtggaacgaaaactcacgttaagg
gattttggtcatgagattatcaaaaaggatcttcacctagatccttttaaattaaaaatgaagttttaaatcaatctaaagtatatatga
gtaaacttggtctgacagttaccaatgcttaatcagtgaggcacctatctcagcgatctgtctatttcgttcatccatagttgcctgactcc
ccgtcgtgtagataactacgatacgggagggcttaccatctggccccagtgctgcaatgataccgcgagacccacgctcaccggctcca
gatttatcagcaataaaccagccagccggaagggccgagcgcagaagtggtcctgcaactttatccgcctccatccagtctattaattgt
tgccgggaagctagagtaagtagttcgccagttaatagtttgcgcaacgttgttgccattgctacaggcatcgtggtgtcacgctcgtcg
tttggtatggcttcattcagctccggttcccaacgatcaaggcgagttacatgatcccccatgttgtgcaaaaaagcggttagctccttcg
gtcctccgatcgttgtcagaagtaagttggccgcagtgttatcactcatggttatggcagcactgcataattctcttactgtcatgccatc
cgtaagatgcttttctgtgactggtgagtactcaaccaagtcattctgagaatagtgtatgcggcgaccgagttgctcttgcccggcgtc
aatacgggataataccgcgccacatagcagaactttaaaagtgctcatcattggaaaacgttcttcggggcgaaaactctcaaggatct
taccgctgttgagatccagttcgatataacccactcgtgcacccaactgatcttcagcatcttttactttcaccagcgtttctgggtgagc
aaaaacaggaaggcaaaatgccgcaaaaaagggaataagggcgacacggaaatgttgaatactcatactcttcctttttcaatattatt
gaagcatttatcagggttattgtctcatgagcggatacatatttgaatgtatttagaaaaataaacaaataggggttccgcgcacatttc
cccgaaaagtgccacctgacgtctaagaaaccattattatcatgacattaacctataaaaataggcgtatcacgag

0.2.12 pSB1A3-*bjaR-gfp* (B0032 | ATG)

gcagaatttcagataaaaaaaatccttagctttcgctaaggatgatttctggaattcgcggccgcttctagagtactgggaaatttccca
atattacgcaagaaaatggtttgttatagtcgaatattactagagtcacacaggaaagtactagATGCGTAAAGGAGAA
GAACTTTTCACTGGAGTTGTCCCAATTCTTGTTGAATTAGATGGTGATGTTAATG
GGCACAAATTTTCTGTCAGTGGAGAGGGTGAAGGTGATGCAACATACGGAAAACT
TACCCTTAAATTTATTTGCACTACTGGAAAACTACCTGTTCCATGGCCAACACTTG
TCACTACTTTCGGTTATGGTGTTCAATGCTTTGCGAGATACCCAGATCATATGAA
ACAGCATGACTTTTTCAAGAGTGCCATGCCCGAAGGTTATGTACAGGAAAGAACT
ATATTTTTCAAAGATGACGGGAACTACAAGACACGTGCTGAAGTCAAGTTTGAAG
GTGATACCCTTGTTAATAGAATCGAGTTAAAAGGTATTGATTTTAAAGAAGATGG
AAACATTCTTGGACACAAATTGGAATACAACTATAACTCACACAATGTATACATC
ATGGCAGACAAACAAAAGAATGGAATCAAAGTTAACTTCAAAATTAGACACAACA
TTGAAGATGGAAGCGTTCAACTAGCAGACCATTATCAACAAAATACTCCAATTGG
CGATGGCCCTGTCCTTTTACCAGACAACCATTACCTGTCCACACAATCTGCCCTTT
CGAAAGATCCCAACGAAAAGAGAGACCACATGGTCCTTCTTGAGTTTGTAACAGC
TGCTGGGATTACACATGGCATGGATGAACTATACAAATAATAAtactagagccggcttatcgg
tcagtttcacctgatttacgtaaaaacccgcttcggcgggtttttgcttttggaggggcagaaagatgaatgactgtccacgacgctata
cccaaaagaaatactagagttgacggctagctcagtcctaggtacagtgctagctactagagtcacacaggaaagtactagATGT
CCGCAGTAGATTATGGGCGTGAAGCCCTGGACTTTATCGAGGGTTTGGGCGTATA
TCGTAAAGTCCCTGATGCGATGAACGCTCTCGAAGCGGCATTCGGTCGCTTTGGC
TTTGAGACAATCATCGTGACTGGGCTGCCCAACCCGGATCAGCGTTTTGCACAAA
TGGTACTCGCTAAACGTTGGCCGGCGGGTTGGTTTAATCTGTATACGCAAAACAA
TTATGACCGTTTTGATCCCGTCGTCCGCTTGTGCCGTCAGAGCGTGAATCCGTTC
GAGTGGAGTGAAGCACCGTATGACGCTGAATTAGAACCAAGCGCCGCCGAAGTCA
TGAATCGTGCAGGCGACTTCCGTATGTCCCGTGGTTTATCGTGCCGATCCATGG
ACTGACAGGATATGAAGCGGCTGTTAGTCTGGGAGGCGTTCATTTAGATCTGAAC
CCGCGTTCCAAACCGGCTCTGCACCTGATGGCAATGTACGGTTTCGACCACATTC
GTCGCCTGCTGGAACCGACCCCATATCCTTCGACGCGCCTTACTCCGCGCGAACG
CGAAGTTATTTCCTGGGCTAGCCAGGGAAAATCTGCGTGGGAAATTGGTGAAATC
CTGCATATCACCCAGCGCACCGCCGAGGAGCACTTAGCGACCGCGGCACGTAAAC
TTGGTGCGGTCAATCGTACCCATGCCGTCGCGCTGGCCATCCGTCACAAAATTAT
CAACCCCTAAtactagagccaggcatcaaataaaacgaaaggctcagtcgaaagactgggcctttcgttttatctgttgtttgt
cggtgaacgctctctactagagtcacactggctcaccttcgggtgggcctttctgcgtttatatactagtagcggccgctgcagtccggca
aaaaagggcaaggtgtcaccaccctgcccttttttctttaaaaccgaaaagattacttcgcgttatgcaggcttcctcgctcactgactcgc
tgcgctcggtcgttcggctgcggcgagcggtatcagctcactcaaaggcggtaatacggttatccacagaatcaggggataacgcagg
aaagaacatgtgagcaaaaggccagcaaaaggccaggaaccgtaaaaaggccgcgttgctggcgtttttccacaggctccgcccccct
gacgagcatcacaaaaatcgacgctcaagtcagaggtggcgaaacccgacaggactataaagataccaggcgtttccccctggaagct
ccctcgtgcgctctcctgttccgaccctgccgcttaccggatacctgtccgcctttctcccttcgggaagcgtggcgctttctcatagctca
cgctgtaggtatctcagttcggtgtaggtcgttcgctccaagctgggctgtgtgcacgaaccccccgttcagcccgaccgctgcgccttat
ccggtaactatcgtcttgagtccaacccggtaagacacgacttatcgccactggcagcagccactggtaacaggattagcagagcgagg
tatgtaggcggtgctacagagttcttgaagtggtggcctaactacggctacactagaagaacagtatttggtatctgcgctctgctgaag
ccagttaccttcggaaaaagagttggtagctcttgatccggcaaacaaaccaccgctggtagcggtggtttttttgtttgcaagcagcag

attacgcgcagaaaaaaaggatctcaagaagatcctttgatcttttctacggggtctgacgctcagtggaacgaaaactcacgttaagg
gattttggtcatgagattatcaaaaaggatcttcacctagatccttttaaattaaaaatgaagttttaaatcaatctaaagtatatatga
gtaaacttggtctgacagttaccaatgcttaatcagtgaggcacctatctcagcgatctgtctatttcgttcatccatagttgcctgactcc
ccgtcgtgtagataactacgatacgggagggcttaccatctggccccagtgctgcaatgataccgcgagacccacgctcaccggctcca
gatttatcagcaataaaccagccagccggaagggccgagcgcagaagtggtcctgcaactttatccgcctccatccagtctattaattgt
tgccgggaagctagagtaagtagttcgccagttaatagtttgcgcaacgttgttgccattgctacaggcatcgtggtgtcacgctcgtcg
tttggtatggcttcattcagctccggttcccaacgatcaaggcgagttacatgatcccccatgttgtgcaaaaaagcggttagctccttcg
gtcctccgatcgttgtcagaagtaagttggccgcagtgttatcactcatggttatggcagcactgcataattctcttactgtcatgccatc
cgtaagatgcttttctgtgactggtgagtactcaaccaagtcattctgagaatagtgtatgcggcgaccgagttgctcttgcccggcgtc
aatacgggataataccgcgccacatagcagaactttaaaagtgctcatcattggaaaacgttcttcggggcgaaaactctcaaggatct
taccgctgttgagatccagttcgatataacccactcgtgcacccaactgatcttcagcatcttttactttcaccagcgtttctgggtgagc
aaaaacaggaaggcaaaatgccgcaaaaaagggaataagggcgacacggaaatgttgaatactcatactcttcctttttcaatattatt
gaagcatttatcagggttattgtctcatgagcggatacatatttgaatgtatttagaaaaataaacaaataggggttccgcgcacatttc
cccgaaaagtgccacctgacgtctaagaaaccattattatcatgacattaacctataaaaataggcgtatcacgag

0.2.13 pSB1A3-*bjaR-gfp* (B0033 | ATG)

gcagaatttcagataaaaaaaaatccttagctttcgctaaggatgatttctggaattcgcggccgcttctagagtactgggaaatttccca
atattacgcaagaaaatggtttgttatagtcgaatattactagagtcacacaggaaagtactagATGCGTAAAGGAGAA
GAACTTTTCACTGGAGTTGTCCCAATTCTTGTTGAATTAGATGGTGATGTTAATG
GGCACAAATTTTCTGTCAGTGGAGAGGGTGAAGGTGATGCAACATACGGAAAACT
TACCCTTAAATTTATTTGCACTACTGGAAAACTACCTGTTCCATGGCCAACACTTG
TCACTACTTTCGGTTATGGTGTTCAATGCTTTGCGAGATACCCAGATCATATGAA
ACAGCATGACTTTTTCAAGAGTGCCATGCCCGAAGGTTATGTACAGGAAAGAACT
ATATTTTTCAAAGATGACGGGAACTACAAGACACGTGCTGAAGTCAAGTTTGAAG
GTGATACCCTTGTTAATAGAATCGAGTTAAAAGGTATTGATTTTAAAGAAGATGG
AAACATTCTTGGACACAAATTGGAATACAACTATAACTCACACAATGTATACATC
ATGGCAGACAAACAAAAGAATGGAATCAAAGTTAACTTCAAAATTAGACACAACA
TTGAAGATGGAAGCGTTCAACTAGCAGACCATTATCAACAAAATACTCCAATTGG
CGATGGCCCTGTCCTTTTACCAGACAACCATTACCTGTCCACACAATCTGCCCTTT
CGAAAGATCCCAACGAAAAGAGAGACCACATGGTCCTTCTTGAGTTTGTAACAGC
TGCTGGGATTACACATGGCATGGATGAACTATACAAATAATAAtactagagccggcttatcgg
tcagtttcacctgatttacgtaaaaacccgcttcggcgggtttttgcttttggaggggcagaaagatgaatgactgtccacgacgctata
cccaaaagaaatactagagttgacggctagctcagtcctaggtacagtgctagctactagagtcacacaggactactagATGTCC
GCAGTAGATTATGGGCGTGAAGCCCTGGACTTTATCGAGGGTTTGGGCGTATATC
GTAAAGTCCCTGATGCGATGAACGCTCTCGAAGCGGCATTCGGTCGCTTTGGCTT
TGAGACAATCATCGTGACTGGGCTGCCCAACCCGGATCAGCGTTTTGCACAAATG
GTACTCGCTAAACGTTGGCCGGCGGGTTGGTTTAATCTGTATACGCAAAACAATT
ATGACCGTTTTGATCCCGTCGTCCGCTTGTGCCGTCAGAGCGTGAATCCGTTCGA
GTGGAGTGAAGCACCGTATGACGCTGAATTAGAACCAAGCGCCGCCGAAGTCATG
AATCGTGCAGGCGACTTCCGTATGTCCCGTGGTTTTATCGTGCCGATCCATGGAC
TGACAGGATATGAAGCGGCTGTTAGTCTGGGAGGCGTTCATTTAGATCTGAACCC

GCGTTCCAAACCGGCTCTGCACCTGATGGCAATGTACGGTTTCGACCACATTCGT
CGCCTGCTGGAACCGACCCCATATCCTTCGACGCGCCTTACTCCGCGCGAACGCG
AAGTTATTTCCTGGGCTAGCCAGGGAAAATCTGCGTGGGAAATTGGTGAAATCCT
GCATATCACCCAGCGCACCGCCGAGGAGCACTTAGCGACCGCGGCACGTAAACTT
GGTGCGGTCAATCGTACCCATGCCGTCGCGCTGGCCATCCGTCACAAAATTATCA
ACCCCTAAtactagagccaggcatcaaataaaacgaaaggctcagtcgaaagactgggcctttcgttttatctgttgtttgtcgg
tgaacgctctctactagagtcacactggctcaccttcgggtgggcctttctgcgtttatatactagtagcggccgctgcagtccggcaaa
aaagggcaaggtgtcaccaccctgccctttttctttaaaaccgaaaagattacttcgcgttatgcaggcttcctcgctcactgactcgctg
cgctcggtcgttcggctgcggcgagcggtatcagctcactcaaaggcggtaatacggttatccacagaatcaggggataacgcaggaa
agaacatgtgagcaaaaggccagcaaaaggccaggaaccgtaaaaaggccgcgttgctggcgtttttccacaggctccgcccccctga
cgagcatcacaaaaatcgacgctcaagtcagaggtggcgaaacccgacaggactataaagataccaggcgtttccccctggaagctcc
ctcgtgcgctctcctgttccgaccctgccgcttaccggatacctgtccgcctttctcccttcgggaagcgtggcgctttctcatagctcacg
ctgtaggtatctcagttcggtgtaggtcgttcgctccaagctgggctgtgtgcacgaaccccccgttcagcccgaccgctgcgccttatcc
ggtaactatcgtcttgagtccaacccggtaagacacgacttatcgccactggcagcagccactggtaacaggattagcagagcgaggta
tgtaggcggtgctacagagttcttgaagtggtggcctaactacggctacactagaagaacagtatttggtatctgcgctctgctgaagcc
agttaccttcggaaaaagagttggtagctcttgatccggcaaacaaaccaccgctggtagcggtggtttttttgtttgcaagcagcagat
tacgcgcagaaaaaaaggatctcaagaagatcctttgatcttttctacggggtctgacgctcagtggaacgaaaactcacgttaaggga
ttttggtcatgagattatcaaaaaggatcttcacctagatccttttaaattaaaaatgaagttttaaatcaatctaaagtatatatgagta
aacttggtctgacagttaccaatgcttaatcagtgaggcacctatctcagcgatctgtctatttcgttcatccatagttgcctgactccccg
tcgtgtagataactacgatacgggagggcttaccatctggccccagtgctgcaatgataccgcgagacccacgctcaccggctccagat
ttatcagcaataaaccagccagccggaagggccgagcgcagaagtggtcctgcaactttatccgcctccatccagtctattaattgttgc
cgggaagctagagtaagtagttcgccagttaatagtttgcgcaacgttgttgccattgctacaggcatcgtggtgtcacgctcgtcgttt
ggtatggcttcattcagctccggttcccaacgatcaaggcgagttacatgatcccccatgttgtgcaaaaaagcggttagctccttcggt
cctccgatcgttgtcagaagtaagttggccgcagtgttatcactcatggttatggcagcactgcataattctcttactgtcatgccatccg
taagatgcttttctgtgactggtgagtactcaaccaagtcattctgagaatagtgtatgcggcgaccgagttgctcttgcccggcgtcaa
tacgggataataccgcgccacatagcagaactttaaaagtgctcatcattggaaaacgttcttcggggcgaaaactctcaaggatctta
ccgctgttgagatccagttcgatataacccactcgtgcacccaactgatcttcagcatcttttactttcaccagcgtttctgggtgagcaa
aaacaggaaggcaaaatgccgcaaaaaagggaataagggcgacacggaaatgttgaatactcatactcttcctttttcaatattattga
agcatttatcagggttattgtctcatgagcggatacatatttgaatgtatttagaaaaataaacaaataggggttccgcgcacatttccc
cgaaaagtgccacctgacgtctaagaaaccattattatcatgacattaacctataaaaataggcgtatcacgag

0.2.14 pSB1A3-*bjaR-gfp* (B0034|CTG)

gcagaatttcagataaaaaaaatccttagctttcgctaaggatgatttctggaattcgcggccgcttctagagtactgggaaatttccca
atattacgcaagaaaatggtttgttatagtcgaatattactagagtcacacaggaaagtactagATGCGTAAAGGAGAA
GAACTTTTCACTGGAGTTGTCCCAATTCTTGTTGAATTAGATGGTGATGTTAATG
GGCACAAATTTTCTGTCAGTGGAGAGGGTGAAGGTGATGCAACATACGGAAAACT
TACCCTTAAATTTATTTGCACTACTGGAAAACTACCTGTTCCATGGCCAACACTTG
TCACTACTTTCGGTTATGGTGTTCAATGCTTTGCGAGATACCCAGATCATATGAA
ACAGCATGACTTTTTCAAGAGTGCCATGCCCGAAGGTTATGTACAGGAAAGAACT
ATATTTTTCAAAGATGACGGGAACTACAAGACACGTGCTGAAGTCAAGTTTGAAG
GTGATACCCTTGTTAATAGAATCGAGTTAAAAGGTATTGATTTTAAAGAAGATGG

AAACATTCTTGGACACAAATTGGAATACAACTATAACTCACACAATGTATACATC
ATGGCAGACAAACAAAAGAATGGAATCAAAGTTAACTTCAAAATTAGACACAACA
TTGAAGATGGAAGCGTTCAACTAGCAGACCATTATCAACAAAATACTCCAATTGG
CGATGGCCCTGTCCTTTTACCAGACAACCATTACCTGTCCACACAATCTGCCCTTT
CGAAAGATCCCAACGAAAAGAGAGACCACATGGTCCTTCTTGAGTTTGTAACAGC
TGCTGGGATTACACATGGCATGGATGAACTATACAAATAATAAtactagagccggcttatcgg
tcagtttcacctgatttacgtaaaaacccgcttcggcgggtttttgcttttggaggggcagaaagatgaatgactgtccacgacgctata
cccaaaagaaatactagagttgacggctagctcagtcctaggtacagtgctagctactagagaaagaggagaaatactagCTGTC
CGCAGTAGATTATGGGCGTGAAGCCCTGGACTTTATCGAGGGTTTGGGCGTATAT
CGTAAAGTCCCTGATGCGATGAACGCTCTCGAAGCGGCATTCGGTCGCTTTGGCT
TTGAGACAATCATCGTGACTGGGCTGCCCAACCCGGATCAGCGTTTTGCACAAAT
GGTACTCGCTAAACGTTGGCCGGCGGGTTGGTTTAATCTGTATACGCAAAACAAT
TATGACCGTTTTGATCCCGTCGTCCGCTTGTGCCGTCAGAGCGTGAATCCGTTCG
AGTGGAGTGAAGCACCGTATGACGCTGAATTAGAACCAAGCGCCGCCGAAGTCAT
GAATCGTGCAGGCGACTTCCGTATGTCCCGTGGTTTTATCGTGCCGATCCATGGA
CTGACAGGATATGAAGCGGCTGTTAGTCTGGGAGGCGTTCATTTAGATCTGAACC
CGCGTTCCAAACCGGCTCTGCACCTGATGGCAATGTACGGTTTCGACCACATTCG
TCGCCTGCTGGAACCGACCCCATATCCTTCGACGCGCCTTACTCCGCGCGAACGC
GAAGTTATTTCCTGGGCTAGCCAGGGAAAATCTGCGTGGGAAATTGGTGAAATCC
TGCATATCACCCAGCGCACCGCCGAGGAGCACTTAGCGACCGCGGCACGTAAACT
TGGTGCGGTCAATCGTACCCATGCCGTCGCGCTGGCCATCCGTCACAAAATTATC
AACCCCTAAtactagagccaggcatcaaataaaacgaaaggctcagtcgaaagactgggcctttcgttttatctgttgtttgtcg
gtgaacgctctctactagagtcacactggctcaccttcgggtgggcctttctgcgtttatatactagtagcggccgctgcagtccggcaa
aaaagggcaaggtgtcaccaccctgccctttttctttaaaaccgaaaagattacttcgcgttatgcaggcttcctcgctcactgactcgct
gcgctcggtcgttcggctgcggcgagcggtatcagctcactcaaaggcggtaatacggttatccacagaatcaggggataacgcagga
aagaacatgtgagcaaaaggccagcaaaaggccaggaaccgtaaaaaggccgcgttgctggcgtttttccacaggctccgcccccctg
acgagcatcacaaaaatcgacgctcaagtcagaggtggcgaaacccgacaggactataaagataccaggcgtttccccctggaagctc
cctcgtgcgctctcctgttccgaccctgccgcttaccggatacctgtccgcctttctcccttcgggaagcgtggcgctttctcatagctcac
gctgtaggtatctcagttcggtgtaggtcgttcgctccaagctgggctgtgtgcacgaaccccccgttcagcccgaccgctgcgccttatc
cggtaactatcgtcttgagtccaacccggtaagacacgacttatcgccactggcagcagccactggtaacaggattagcagagcgaggt
atgtaggcggtgctacagagttcttgaagtggtggcctaactacggctacactagaagaacagtatttggtatctgcgctctgctgaagc
cagttaccttcggaaaaagagttggtagctcttgatccggcaaacaaaccaccgctggtagcggtggtttttttgtttgcaagcagcaga
ttacgcgcagaaaaaaaggatctcaagaagatcctttgatcttttctacggggtctgacgctcagtggaacgaaaactcacgttaaggg
attttggtcatgagattatcaaaaaggatcttcacctagatccttttaaattaaaaatgaagttttaaatcaatctaaagtatatatgagt
aaacttggtctgacagttaccaatgcttaatcagtgaggcacctatctcagcgatctgtctatttcgttcatccatagttgcctgactcccc
gtcgtgtagataactacgatacgggagggcttaccatctggccccagtgctgcaatgataccgcgagacccacgctcaccggctccaga
tttatcagcaataaaccagccagccggaagggccgagcgcagaagtggtcctgcaactttatccgcctccatccagtctattaattgttg
ccgggaagctagagtaagtagttcgccagttaatagtttgcgcaacgttgttgccattgctacaggcatcgtggtgtcacgctcgtcgtt
tggtatggcttcattcagctccggttcccaacgatcaaggcgagttacatgatcccccatgttgtgcaaaaaagcggttagctccttcgg
tcctccgatcgttgtcagaagtaagttggccgcagtgttatcactcatggttatggcagcactgcataattctcttactgtcatgccatcc
gtaagatgcttttctgtgactggtgagtactcaaccaagtcattctgagaatagtgtatgcggcgaccgagttgctcttgcccggcgtca
atacgggataataccgcgccacatagcagaactttaaaagtgctcatcattggaaaacgttcttcggggcgaaaactctcaaggatctt
accgctgttgagatccagttcgatataacccactcgtgcacccaactgatcttcagcatcttttactttcaccagcgtttctgggtgagca

aaaacaggaaggcaaaatgccgcaaaaaagggaataagggcgacacggaaatgttgaatactcatactcttcctttttcaatattattg
aagcatttatcagggttattgtctcatgagcggatacatatttgaatgtatttagaaaaataaacaaataggggttccgcgcacatttcc
ccgaaaagtgccacctgacgtctaagaaaccattattatcatgacattaacctataaaaataggcgtatcacgag

0.2.15 pSB1A3-*bjaR-gfp* (B0034 | ACG)

gcagaatttcagataaaaaaaatccttagctttcgctaaggatgatttctggaattcgcggccgcttctagagtactgggaaatttccca
atattacgcaagaaaatggtttgttatagtcgaatattactagagtcacacaggaaagtactagATGCGTAAAGGAGAA
GAACTTTTCACTGGAGTTGTCCCAATTCTTGTTGAATTAGATGGTGATGTTAATG
GGCACAAATTTTCTGTCAGTGGAGAGGGTGAAGGTGATGCAACATACGGAAAACT
TACCCTTAAATTTATTTGCACTACTGGAAAACTACCTGTTCCATGGCCAACACTTG
TCACTACTTTCGGTTATGGTGTTCAATGCTTTGCGAGATACCCAGATCATATGAA
ACAGCATGACTTTTTCAAGAGTGCCATGCCCGAAGGTTATGTACAGGAAAGAACT
ATATTTTTCAAAGATGACGGGAACTACAAGACACGTGCTGAAGTCAAGTTTGAAG
GTGATACCCTTGTTAATAGAATCGAGTTAAAAGGTATTGATTTTAAAGAAGATGG
AAACATTCTTGGACACAAATTGGAATACAACTATAACTCACACAATGTATACATC
ATGGCAGACAAACAAAAGAATGGAATCAAAGTTAACTTCAAAATTAGACACAACA
TTGAAGATGGAAGCGTTCAACTAGCAGACCATTATCAACAAAATACTCCAATTGG
CGATGGCCCTGTCCTTTTACCAGACAACCATTACCTGTCCACACAATCTGCCCTTT
CGAAAGATCCCAACGAAAAGAGAGACCACATGGTCCTTCTTGAGTTTGTAACAGC
TGCTGGGATTACACATGGCATGGATGAACTATACAAATAATAAtactagagccggcttatcgg
tcagtttcacctgatttacgtaaaaacccgcttcggcgggtttttgcttttggaggggcagaaagatgaatgactgtccacgacgctata
cccaaaagaaatactagagttgacggctagctcagtcctaggtacagtgctagctactagagaaagaggagaaatactagACGTC
CGCAGTAGATTATGGGCGTGAAGCCCTGGACTTTATCGAGGGTTTGGGCGTATAT
CGTAAAGTCCCTGATGCGATGAACGCTCTCGAAGCGGCATTCGGTCGCTTTGGCT
TTGAGACAATCATCGTGACTGGGCTGCCCAACCCGGATCAGCGTTTTGCACAAAT
GGTACTCGCTAAACGTTGGCCGGCGGGTTGGTTTAATCTGTATACGCAAAACAAT
TATGACCGTTTTGATCCCGTCGTCCGCTTGTGCCGTCAGAGCGTGAATCCGTTCG
AGTGGAGTGAAGCACCGTATGACGCTGAATTAGAACCAAGCGCCGCCGAAGTCAT
GAATCGTGCAGGCGACTTCCGTATGTCCCGTGGTTTTATCGTGCCGATCCATGGA
CTGACAGGATATGAAGCGGCTGTTAGTCTGGGAGGCGTTCATTTAGATCTGAACC
CGCGTTCCAAACCGGCTCTGCACCTGATGGCAATGTACGGTTTCGACCACATTCG
TCGCCTGCTGGAACCGACCCCATATCCTTCGACGCGCCTTACTCCGCGCGAACGC
GAAGTTATTTCCTGGGCTAGCCAGGGAAAATCTGCGTGGGAAATTGGTGAAATCC
TGCATATCACCCAGCGCACCGCCGAGGAGCACTTAGCGACCGCGGCACGTAAACT
TGGTGCGGTCAATCGTACCCATGCCGTCGCGCTGGCCATCCGTCACAAAATTATC
AACCCCTAAtactagagccaggcatcaaataaaacgaaaggctcagtcgaaagactgggcctttcgttttatctgttgtttgtcg
gtgaacgctctctactagagtcacactggctcaccttcgggtgggcctttctgcgtttatatactagtagcggccgctgcagtccggcaa
aaaagggcaaggtgtcaccaccctgccctttttctttaaaaccgaaaagattacttcgcgttatgcaggcttcctcgctcactgactcgct
gcgctcggtcgttcggctgcggcgagcggtatcagctcactcaaaggcggtaatacggttatccacagaatcaggggataacgcagga
aagaacatgtgagcaaaaggccagcaaaaggccaggaaccgtaaaaaggccgcgttgctggcgtttttccacaggctccgcccccctg
acgagcatcacaaaaatcgacgctcaagtcagaggtggcgaaacccgacaggactataaagataccaggcgtttccccctggaagctc

cctcgtgcgctctcctgttccgaccctgccgcttaccggatacctgtccgcctttctcccttcgggaagcgtggcgctttctcatagctcac
gctgtaggtatctcagttcggtgtaggtcgttcgctccaagctgggctgtgtgcacgaaccccccgttcagcccgaccgctgcgccttatc
cggtaactatcgtcttgagtccaacccggtaagacacgacttatcgccactggcagcagccactggtaacaggattagcagagcgaggt
atgtaggcggtgctacagagttcttgaagtggtggcctaactacggctacactagaagaacagtatttggtatctgcgctctgctgaagc
cagttaccttcggaaaaagagttggtagctcttgatccggcaaacaaaccaccgctggtagcggtggtttttttgtttgcaagcagcaga
ttacgcgcagaaaaaaaggatctcaagaagatcctttgatcttttctacggggtctgacgctcagtggaacgaaaactcacgttaaggg
attttggtcatgagattatcaaaaaggatcttcacctagatccttttaaattaaaaatgaagttttaaatcaatctaaagtatatatgagt
aaacttggtctgacagttaccaatgcttaatcagtgaggcacctatctcagcgatctgtctatttcgttcatccatagttgcctgactcccc
gtcgtgtagataactacgatacgggagggcttaccatctggccccagtgctgcaatgataccgcgagacccacgctcaccggctccaga
tttatcagcaataaaccagccagccggaagggccgagcgcagaagtggtcctgcaactttatccgcctccatccagtctattaattgttg
ccgggaagctagagtaagtagttcgccagttaatagtttgcgcaacgttgttgccattgctacaggcatcgtggtgtcacgctcgtcgtt
tggtatggcttcattcagctccggttcccaacgatcaaggcgagttacatgatcccccatgttgtgcaaaaaagcggttagctccttcgg
tcctccgatcgttgtcagaagtaagttggccgcagtgttatcactcatggttatggcagcactgcataattctcttactgtcatgccatcc
gtaagatgcttttctgtgactggtgagtactcaaccaagtcattctgagaatagtgtatgcggcgaccgagttgctcttgcccggcgtca
atacgggataataccgcgccacatagcagaactttaaaagtgctcatcattggaaaacgttcttcggggcgaaaactctcaaggatctt
accgctgttgagatccagttcgatataacccactcgtgcacccaactgatcttcagcatcttttactttcaccagcgtttctgggtgagca
aaaacaggaaggcaaaatgccgcaaaaaagggaataagggcgacacggaaatgttgaatactcatactcttcctttttcaatattattg
aagcatttatcagggttattgtctcatgagcggatacatatttgaatgtatttagaaaaataaacaaataggggttccgcgcacatttcc
ccgaaaagtgccacctgacgtctaagaaaccattattatcatgacattaacctataaaaataggcgtatcacgag

0.2.16 pSB1A3-*bjaR*$_{S107R}$-*gfp* (B0034 | CTG)

gcagaatttcagataaaaaaaatccttagctttcgctaaggatgatttctggaattcgcggccgcttctagagtactgggaaatttccca
atattacgcaagaaaatggtttgttatagtcgaatattactagagtcacacaggaaagtactagATGCGTAAAGGAGAA
GAACTTTTCACTGGAGTTGTCCCAATTCTTGTTGAATTAGATGGTGATGTTAATG
GGCACAAATTTTCTGTCAGTGGAGAGGGTGAAGGTGATGCAACATACGGAAAACT
TACCCTTAAATTTATTTGCACTACTGGAAAACTACCTGTTCCATGGCCAACACTTG
TCACTACTTTCGGTTATGGTGTTCAATGCTTTGCGAGATACCCAGATCATATGAA
ACAGCATGACTTTTTCAAGAGTGCCATGCCCGAAGGTTATGTACAGGAAAGAACT
ATATTTTTCAAAGATGACGGGAACTACAAGACACGTGCTGAAGTCAAGTTTGAAG
GTGATACCCTTGTTAATAGAATCGAGTTAAAAGGTATTGATTTTAAAGAAGATGG
AAACATTCTTGGACACAAATTGGAATACAACTATAACTCACACAATGTATACATC
ATGGCAGACAAACAAAAGAATGGAATCAAAGTTAACTTCAAAATTAGACACAACA
TTGAAGATGGAAGCGTTCAACTAGCAGACCATTATCAACAAAATACTCCAATTGG
CGATGGCCCTGTCCTTTTACCAGACAACCATTACCTGTCCACACAATCTGCCCTTT
CGAAAGATCCCAACGAAAAGAGAGACCACATGGTCCTTCTTGAGTTTGTAACAGC
TGCTGGGATTACACATGGCATGGATGAACTATACAAATAATAAtactagagccggcttatcgg
tcagtttcacctgatttacgtaaaaacccgcttcggcgggtttttgcttttggaggggcagaaagatgaatgactgtccacgacgctata
cccaaaagaaatactagagttgacggctagctcagtcctaggtacagtgctagctactagagaaagaggagaaatactagCTGTC
CGCAGTAGATTATGGGCGTGAAGCCCTGGACTTTATCGAGGGTTTGGGCGTATAT
CGTAAAGTCCCTGATGCGATGAACGCTCTCGAAGCGGCATTCGGTCGCTTTGGCT
TTGAGACAATCATCGTGACTGGGCTGCCCAACCCGGATCAGCGTTTTGCACAAAT

GGTACTCGCTAAACGTTGGCCGGCGGGTTGGTTTAATCTGTATACGCAAAACAAT
TATGACCGTTTTGATCCCGTCGTCCGCTTGTGCCGTCAGAGCGTGAATCCGTTCG
AGTGGAGTGAAGCACCGTATGACGCTGAATTAGAACCACGCGCCGCCGAAGTCAT
GAATCGTGCAGGCGACTTCCGTATGTCCCGTGGTTTTATCGTGCCGATCCATGGA
CTGACAGGATATGAAGCGGCTGTTAGTCTGGGAGGCGTTCATTTAGATCTGAACC
CGCGTTCCAAACCGGCTCTGCACCTGATGGCAATGTACGGTTTCGACCACATTCG
TCGCCTGCTGGAACCGACCCCATATCCTTCGACGCGCCTTACTCCGCGCGAACGC
GAAGTTATTTCCTGGGCTAGCCAGGGAAAATCTGCGTGGGAAATTGGTGAAATCC
TGCATATCACCCAGCGCACCGCCGAGGAGCACTTAGCGACCGCGGCACGTAAACT
TGGTGCGGTCAATCGTACCCATGCCGTCGCGCTGGCCATCCGTCACAAAATTATC
AACCCCTAAtactagagccaggcatcaaataaaacgaaaggctcagtcgaaagactgggcctttcgttttatctgttgtttgtcg
gtgaacgctctctactagagtcacactggctcaccttcgggtgggcctttctgcgtttatatactagtagcggccgctgcagtccggcaa
aaaagggcaaggtgtcaccaccctgccctttttctttaaaaccgaaaagattacttcgcgttatgcaggcttcctcgctcactgactcgct
gcgctcggtcgttcggctgcggcgagcggtatcagctcactcaaaggcggtaatacggttatccacagaatcaggggataacgcagga
aagaacatgtgagcaaaaggccagcaaaaggccaggaaccgtaaaaaggccgcgttgctggcgtttttccacaggctccgcccccctg
acgagcatcacaaaaatcgacgctcaagtcagaggtggcgaaacccgacaggactataaagataccaggcgtttccccctggaagctc
cctcgtgcgctctcctgttccgaccctgccgcttaccggatacctgtccgcctttctcccttcgggaagcgtggcgctttctcatagctcac
gctgtaggtatctcagttcggtgtaggtcgttcgctccaagctgggctgtgtgcacgaaccccccgttcagcccgaccgctgcgccttatc
cggtaactatcgtcttgagtccaacccggtaagacacgacttatcgccactggcagcagccactggtaacaggattagcagagcgaggt
atgtaggcggtgctacagagttcttgaagtggtggcctaactacggctacactagaagaacagtatttggtatctgcgctctgctgaagc
cagttaccttcggaaaaagagttggtagctcttgatccggcaaacaaaccaccgctggtagcggtggtttttttgtttgcaagcagcaga
ttacgcgcagaaaaaaaggatctcaagaagatcctttgatcttttctacggggtctgacgctcagtggaacgaaaactcacgttaaggg
attttggtcatgagattatcaaaaaggatcttcacctagatccttttaaattaaaaatgaagttttaaatcaatctaaagtatatatgagt
aaacttggtctgacagttaccaatgcttaatcagtgaggcacctatctcagcgatctgtctatttcgttcatccatagttgcctgactcccc
gtcgtgtagataactacgatacgggagggcttaccatctggccccagtgctgcaatgataccgcgagacccacgctcaccggctccaga
tttatcagcaataaaccagccagccggaagggccgagcgcagaagtggtcctgcaactttatccgcctccatccagtctattaattgttg
ccgggaagctagagtaagtagttcgccagttaatagtttgcgcaacgttgttgccattgctacaggcatcgtggtgtcacgctcgtcgtt
tggtatggcttcattcagctccggttcccaacgatcaaggcgagttacatgatcccccatgttgtgcaaaaaagcggttagctccttcgg
tcctccgatcgttgtcagaagtaagttggccgcagtgttatcactcatggttatggcagcactgcataattctcttactgtcatgccatcc
gtaagatgcttttctgtgactggtgagtactcaaccaagtcattctgagaatagtgtatgcggcgaccgagttgctcttgcccggcgtca
atacgggataataccgcgccacatagcagaactttaaaagtgctcatcattggaaaacgttcttcggggcgaaaactctcaaggatctt
accgctgttgagatccagttcgatataacccactcgtgcacccaactgatcttcagcatcttttactttcaccagcgtttctgggtgagca
aaaacaggaaggcaaaatgccgcaaaaaagggaataagggcgacacggaaatgttgaatactcatactcttcctttttcaatattattg
aagcatttatcagggttattgtctcatgagcggatacatatttgaatgtatttagaaaaataaacaaataggggttccgcgcacatttcc
ccgaaaagtgccacctgacgtctaagaaaccattattatcatgacattaacctataaaaataggcgtatcacgag

0.2.17 pSB1A3-$bjaR_{S107R}$-*gfp* (B0033 | ATG)

gcagaatttcagataaaaaaaatccttagctttcgctaaggatgatttctggaattcgcggccgcttctagagtactgggaaatttccca
atattacgcaagaaaatggtttgttatagtcgaatattactagagtcacacaggaaagtactagATGCGTAAAGGAGAA
GAACTTTTCACTGGAGTTGTCCCAATTCTTGTTGAATTAGATGGTGATGTTAATG
GGCACAAATTTTCTGTCAGTGGAGAGGGTGAAGGTGATGCAACATACGGAAAACT

TACCCTTAAATTTATTTGCACTACTGGAAAACTACCTGTTCCATGGCCAACACTTG
TCACTACTTTCGGTTATGGTGTTCAATGCTTTGCGAGATACCCAGATCATATGAA
ACAGCATGACTTTTTCAAGAGTGCCATGCCCGAAGGTTATGTACAGGAAAGAACT
ATATTTTTCAAAGATGACGGGAACTACAAGACACGTGCTGAAGTCAAGTTTGAAG
GTGATACCCTTGTTAATAGAATCGAGTTAAAAGGTATTGATTTTAAAGAAGATGG
AAACATTCTTGGACACAAATTGGAATACAACTATAACTCACACAATGTATACATC
ATGGCAGACAAACAAAAGAATGGAATCAAAGTTAACTTCAAAATTAGACACAACA
TTGAAGATGGAAGCGTTCAACTAGCAGACCATTATCAACAAAATACTCCAATTGG
CGATGGCCCTGTCCTTTTACCAGACAACCATTACCTGTCCACACAATCTGCCCTTT
CGAAAGATCCCAACGAAAAGAGAGACCACATGGTCCTTCTTGAGTTTGTAACAGC
TGCTGGGATTACACATGGCATGGATGAACTATACAAATAATAAtactagagccggcttatcgg
tcagtttcacctgatttacgtaaaaacccgcttcggcgggtttttgcttttggaggggcagaaagatgaatgactgtccacgacgctata
cccaaaagaaatactagagttgacggctagctcagtcctaggtacagtgctagctactagagtcacacaggactactagATGTCC
GCAGTAGATTATGGGCGTGAAGCCCTGGACTTTATCGAGGGTTTGGGCGTATATC
GTAAAGTCCCTGATGCGATGAACGCTCTCGAAGCGGCATTCGGTCGCTTTGGCTT
TGAGACAATCATCGTGACTGGGCTGCCCAACCCGGATCAGCGTTTTGCACAAATG
GTACTCGCTAAACGTTGGCCGGCGGGTTGGTTTAATCTGTATACGCAAAACAATT
ATGACCGTTTTGATCCCGTCGTCCGCTTGTGCCGTCAGAGCGTGAATCCGTTCGA
GTGGAGTGAAGCACCGTATGACGCTGAATTAGAACCACGCGCCGCCGAAGTCATG
AATCGTGCAGGCGACTTCCGTATGTCCCGTGGTTTTATCGTGCCGATCCATGGAC
TGACAGGATATGAAGCGGCTGTTAGTCTGGGAGGCGTTCATTTAGATCTGAACCC
GCGTTCCAAACCGGCTCTGCACCTGATGGCAATGTACGGTTTCGACCACATTCGT
CGCCTGCTGGAACCGACCCCATATCCTTCGACGCGCCTTACTCCGCGCGAACGCG
AAGTTATTTCCTGGGCTAGCCAGGGAAAATCTGCGTGGGAAATTGGTGAAATCCT
GCATATCACCCAGCGCACCGCCGAGGAGCACTTAGCGACCGCGGCACGTAAACTT
GGTGCGGTCAATCGTACCCATGCCGTCGCGCTGGCCATCCGTCACAAAATTATCA
ACCCCTAAtactagagccaggcatcaaataaaacgaaaggctcagtcgaaagactgggcctttcgttttatctgttgtttgtcgg
tgaacgctctctactagagtcacactggctcaccttcgggtgggcctttctgcgtttatatactagtagcggccgctgcagtccggcaaa
aaagggcaaggtgtcaccaccctgccctttttctttaaaaccgaaaagattacttcgcgttatgcaggcttcctcgctcactgactcgctg
cgctcggtcgttcggctgcggcgagcggtatcagctcactcaaaggcggtaatacggttatccacagaatcaggggataacgcaggaa
agaacatgtgagcaaaaggccagcaaaaggccaggaaccgtaaaaaggccgcgttgctggcgtttttccacaggctccgcccccctga
cgagcatcacaaaaatcgacgctcaagtcagaggtggcgaaacccgacaggactataaagataccaggcgtttccccctggaagctcc
ctcgtgcgctctcctgttccgaccctgccgcttaccggatacctgtccgcctttctcccttcgggaagcgtggcgctttctcatagctcacg
ctgtaggtatctcagttcggtgtaggtcgttcgctccaagctgggctgtgtgcacgaaccccccgttcagcccgaccgctgcgccttatcc
ggtaactatcgtcttgagtccaacccggtaagacacgacttatcgccactggcagcagccactggtaacaggattagcagagcgaggta
tgtaggcggtgctacagagttcttgaagtggtggcctaactacggctacactagaagaacagtatttggtatctgcgctctgctgaagcc
agttaccttcggaaaaagagttggtagctcttgatccggcaaacaaaccaccgctggtagcggtggtttttttgtttgcaagcagcagat
tacgcgcagaaaaaaaggatctcaagaagatcctttgatcttttctacggggtctgacgctcagtggaacgaaaactcacgttaaggga
ttttggtcatgagattatcaaaaaggatcttcacctagatccttttaaattaaaaatgaagttttaaatcaatctaaagtatatatgagta
aacttggtctgacagttaccaatgcttaatcagtgaggcacctatctcagcgatctgtctatttcgttcatccatagttgcctgactccccg
tcgtgtagataactacgatacgggagggcttaccatctggccccagtgctgcaatgataccgcgagacccacgctcaccggctccagat
ttatcagcaataaaccagccagccggaagggccgagcgcagaagtggtcctgcaactttatccgcctccatccagtctattaattgttgc
cgggaagctagagtaagtagttcgccagttaatagtttgcgcaacgttgttgccattgctacaggcatcgtggtgtcacgctcgtcgttt

ggtatggcttcattcagctccggttcccaacgatcaaggcgagttacatgatcccccatgttgtgcaaaaaagcggttagctccttcggt
cctccgatcgttgtcagaagtaagttggccgcagtgttatcactcatggttatggcagcactgcataattctcttactgtcatgccatccg
taagatgcttttctgtgactggtgagtactcaaccaagtcattctgagaatagtgtatgcggcgaccgagttgctcttgcccggcgtcaa
tacgggataataccgcgccacatagcagaactttaaaagtgctcatcattggaaaacgttcttcggggcgaaaactctcaaggatctta
ccgctgttgagatccagttcgatataacccactcgtgcacccaactgatcttcagcatcttttactttcaccagcgtttctgggtgagcaa
aaacaggaaggcaaaatgccgcaaaaaagggaataagggcgacacggaaatgttgaatactcatactcttcctttttcaatattattga
agcatttatcagggttattgtctcatgagcggatacatatttgaatgtatttagaaaaataaacaaataggggttccgcgcacatttccc
cgaaaagtgccacctgacgtctaagaaaccattattatcatgacattaacctataaaaataggcgtatcacgag

0.3 Supplementary methods

0.3.1 pPURE-*mNG* assembly

The *mNG* gene was amplified by PCR using Phusion DNA polymerase mastermix containing 1 ng pMAT mNeongreen plasmid and 500 nM T7 for/PURE rev 1 primers with the following thermal cycler programme: 98°C 30 s, 35 cycles of [98 °C for 10 s, 59 °C for 20 s, 72 °C for 45 s], 72 °C for 10 minutes, 4 °C HOLD. 1 μg of PCR product and PURExpress control template plasmid were digested with XbaI/BamHI-HF for 1 hour at 37 °C and then ran on a 1.2% TAE agarose gel prestained with 3% GelRed at 100 V for 1 hour. Bands corresponding with the double digested DNA were cut from the gel with a scalpel and purified from the gel using QIAquick gel extraction purification kit. The *mNG* gene insert was ligated with 50 ng plasmid backbone using T4 DNA ligase (3:1 insert:vector ratio) and 2 μL of ligation was transformed into 10 μL *E. coli* XL10 Gold cells by heat shock. Cells were incubated in 500 μL SOC media for 45 minutes, then 100 μL was plated onto LB agar containing 100 μg/mL ampicillin. Plates were incubated at 37°C for ~14 hours. Colonies were picked with pipette tips and grown as 5 mL liquid cultures in LB + ampicillin (100 ug/mL) at 37 °C, 225 rpm, overnight. Plasmids were harvested from 3 mL overnight culture using a QIAprep spin mini-prep kit and verified by Sanger sequencing.

0.3.2 pPURE-T7g10-*gp10(1-9)::mNG* assembly

The T7g10 leader sequence was introduced into the pPURE-*mNG* plasmid via the recombination of a single DNA fragment with homologous ends, denoted mNG T7g10 BB[2]. *mNG* T7g10 BB was prepared by nested PCR. First, *mNG* T7g10 fragment 1 was amplified with Phusion DNA polymerase mastermix (2X) using 500 nM *mNG*_T7g10_for1 and *mNG*_T7g10_rev1 primers and 1 ng pPURE-*mNG* template with the following thermal cycler programme: 98 °C for 30s, 35 cycles of [98 °C for 10 s, 63 °C for 20 s, 72 °C for 1 min 30 s], 72 °C for 10 mins, 4 °C HOLD. *mNG* T7g10 BB was then amplified from 1 ng *mNG* T7g10 fragment 1 using Phusion DNA polymerase mastermix (2X) and 500 nM *mNG*_T7g10_for2 and *mNG*_T7g10_rev2 primers with the following thermal cycler programme: 98 °C for 30s, 35 cycles of [98 °C for 10 s, 63 °C for 20

s, 72 °C for 1 min 30 s], 72 °C for 10 mins, 4 °C. After amplification, 10 U of DpnI was added to the reaction mix and incubated at 37 °C for 1 hour, followed by 20 mins at 80 °C. The PCR product was purified from the reaction mixture using a QIAquick spin PCR purification kit. 100 ng of *mNG* T7g10 BB was transformed into 10 μL *E. coli* XL10-Gold cells by heat shock. Cells were incubated in 500 μL SOC media for 45 minutes, then 100 μL was plated onto LB agar containing 100 μg/mL ampicillin. Plates were incubated at 37 °C for ∼14 hours. Colonies were picked with pipette tips and grown as 5 mL liquid cultures in LB + ampicillin (100 μg/mL) at 37 °C, 225 rpm, overnight. Plasmids were harvested from 3 mL overnight culture using a QIAprep spin mini-prep kit and verified by Sanger sequencing.

0.3.3 pPURE-*gp10(1-9)::mNG* assembly

The 10 AA leader sequence (9 AAs from the T7 bacteriophage major capsid protein + 1 additional AA (Histidine) present in[3]) was introduced into the pPURE-*mNG* plasmid via the recombination of 2 DNA strands with homologous ends, denoted *mNG* g10 insert and *mNG* g10 BB. mNG g10 insert was amplified with Phusion DNA polymerase mastermix (2X) using 500 nM *mNG*_g10_for and PURE_rev2 primers and 1 ng pPURE-*mNG* T7g10 template with the following thermal cycler programme: 98 °C for 30 s, 35 cycles of [98 °C for 10 s, 66 °C for 20 s and 72 for °C for 30 s], 72 °C for 5 mins, 4 °C HOLD. *mNG* g10 BB was amplified with Phusion DNA polymerase mastermix (2X) using 500 nM PURE_for1 and *mNG*_g10_rev primers and 1 ng pPURE-*mNG* template with the following thermal cycler programme: 98 °C 30 s, 35 cycles of [98 °C for 10 s, °C for 20 s and 72 °C for 1 min 15 s], 72 °C for 5 mins, 4 °C HOLD. After amplification, 10 U of DpnI was added to the reactions and incubated at 37 °C for 1 hour, followed by 20 mins at 80 °C. The PCR products were purified from the reaction mixture using a QIAquick spin PCR purification kit. Insert and BB were combined at a 3:1 mol ratio (100 ng of *mNG* g10 BB) and transformed into 10 μL *E. coli* XL10-Gold cells by heat shock. Cells were incubated in 500 μL SOC media for 45 minutes, then 100 μL was plated onto LB agar containing 100 μg/mL ampicillin. Plates were incubated at 37 °C for ∼14 hours. Colonies were picked with pipette tips and grown as 5 mL liquid cultures in LB + ampicillin (100 μg/mL) at 37 °C, 225 rpm, overnight. Plasmids were harvested from 3 mL overnight culture using a QIAprep spin mini-prep kit and verified by Sanger sequencing.

0.3.4 pPURE-T7g10-*gp10(1-9)::mVenus* assembly

The T7g10 leader sequence was introduced into the pPURE-*mVenus* plasmid via the recombination of 2 DNA strands with homologous ends, denoted *mVenus*_T7g10_insert and *mVenus*_T7g10_BB. *mVenus*_T7g10_insert was amplified with Phusion DNA polymerase mastermix (2X) using 500 nM mV_T7g10_for and PURE_rev2 primers and 1 ng pPURE-*mVenus* template with the following thermal cycler programme: 98 °C for 30 s, 35 cycles of [98 °C for 10 s, 66 °C for 20 s and 72 for °C for 30 s], 72 °C for 10 mins, 4 °C HOLD. *mVenus* T7g10 BB was amplified with Phusion DNA polymerase mastermix (2X) using 500 nM PURE_for1 and mV_T7g10_rev

primers and 1 ng pPURE-T7g10-*mNG* template with the following thermal cycler programme: 98 °C 30 s, 35 cycles of [98 °C for 10 s, 66 °C for 20 s and 72 °C for 1 min 15 s], 72 °C for 5 mins, 4 °C HOLD. Two fragment homologous recombination was performed as described for pPURE-*mNG*.

0.3.5 pPURE-*bjaI* assembly

The *bjaI* gene sequence was obtained from the European Nucleotide Archive database (Sequence BA000040.2)[4]. Short ∼60 nt oligonucleotides (*bjaI*_for1-9/Bjai_rev1-8) with ∼20 nt terminal overhangs were designed to collectively encode the full *bjaI* gene sequence. Oligos *bjaI*_for1-9 and *bjaI*_rev1-8 were pooled to a final concentration of 5 μM, then annealed and elongated via polymerase chain assembly[5] (100 nM oligo mix, 1X Phusion DNA polymerase mastermix, 3% DMSO) with the following thermal cycler programme (98 °C 2 mins, 30 cycles of [98 °C for 10 s, 60 °C for 20 s and 72 °C for 15 s], 72°C for 5 mins, 4 °C HOLD). The complete *bjaI* gene was amplified from the pool of assembled oligos via a second PCR step with amplification primers *bjaI*_amp_for and *bjaI*_amp_rev. *bjaI* PCR product and pPURE-*mNG* plasmid were double digested with XbaI/BamHI-HF and NdeI/EcoRI-HF and ran on a 1.2% TAE agarose gel. Digested DNA was cut out from the agarose gel and purified using the Monarch gel extraction purification kit. The *bjaI* gene was ligated into digested pPURE plasmid using the same protocol as described above.

0.3.6 pSB1A3-$bjaR_{KO}$-*gfp* assembly

pSB1A3-$bjaR_{KO}$ *gfp* BB was prepared by the homologous recombination of a single DNA fragment with homologous ends, denoted $bjaR_{\mathrm{KO}}$ BB. To knockout *bjaR* activity, the *bjaR* gene was truncated by introducing two stop codons followed by a randomised DNA sequence after position 12. Nucleotides for AA1-12 were left to assist in cloning the plasmid. $bjaR_{\mathrm{KO}}$ BB was amplified with Phusion DNA polymerase mastermix (2X) using 500 nM *bjaR*_KO_for and *bjaR*_KO_rev primers and 1 ng pSB1A3 $bjaR_{WT}$ *gfp* template with the following thermal cycler programme: 98 °C for 30s, 35 cycles of [98 °C for 10 s, 65 °C for 20 s, 72 °C for 1 min], 72 °C for 5 mins, 4 °C HOLD. Single fragment homologous recombination was performed as described above.

0.3.7 pSB1A3-*bjaR-gfp-kanR* and pSB1A3-$bjaR_{KO}$-*gfp-kanR* assembly

pSB1A3 $bjaR_{KO/WT}$-*gfp-kanR* were prepared via the recombination of two DNA strands with homologous ends, denoted $bjaR_{KO/WT}$ *kanR* BB and *kanR* insert. The $bjaR_{KO/WT}$ *kanR* BB fragments were amplified with Phusion DNA polymerase mastermix (2X) using 500 nM pSB1A3_for3 and pSB1A3_rev3 primers and 1 ng pSB1A3 $bjaR_{KO/WT}$ *gfp* templates with the following thermal cycler programme: 98 °C for 30 s, 35 cycles of [98 °C for 10 s, 65 °C/63 °C for 20 s and 72 °C for 1 min/2 mins], 72 °C for 10 mins, 4 °C HOLD. The *kanR* insert

fragment was created by nested PCR. *kanR* was first amplified with Phusion DNA polymerase mastermix (2X) using 500 nM *kanR*_for1 and *kanR*_rev primers and 1 ng pET24a template with the following thermal cycler programme: 98 °C for 30 s, 35 cycles of [98 °C for 10 s, 66 °C for 20 s and 72 °C for 30 s], 72 °C for 5 mins, 4 °C HOLD. This PCR product was purified using a QIAquick PCR purification kit then 1 ng was used as the DNA template in a second PCR with 500 nM *kanR*_for2 and *kanR*_rev primers according to the following protocol: 98 °C for 30 s, 35 cycles of [98 °C for 10 s, 65 °C for 20 s and 72 °C for 30 s], 72 °C for 5 mins, 4 °C HOLD. Two fragment homologous recombination was performed as described above.

0.3.8 pSB1A3-*bjaR*$_{\mathbf{S107R}}$-*gfp* (B0034 | CTG) assembly

pSB1A3-*BjaR*$_{S107R}$-*gfp* (B0034 | CTG) was prepared by transferring the *bjaR*$_{\mathrm{S107R}}$ gene with a CTG start codon from the *kanR*-containing reporter plasmid backbone used in directed evolution workflow back into the *kanR*-free backbone. pSB1A3-*bjaR*$_{S107R}$-*gfp* (B0034 | CTG) was prepared via the recombination of two DNA strands with homologous ends, denoted pSB1A3-*gfp*-BB and *bjaR*$_{\mathrm{S107R}}$ (CTG) insert. The pSB1A3-*gfp*-BB fragment was amplified with Phusion DNA polymerase mastermix (2X) using 500 nM pSB1A3_for2 and pSB1A3_rev2 primers and 1 ng pSB1A3-*bjaR-gfp* template with the following thermal cycler programme: 98 °C for 30 s, 35 cycles of [98 °C for 10 s, 63 °C for 20 s and 72 °C for 2 mins], 72 °C for 10 mins, 4 °C HOLD. The *bjaR*$_{\mathrm{S107R}}$ (CTG) insert fragment was amplified with Phusion DNA polymerase mastermix (2X) using 500 nM pSB1A3_for1 and pSB1A3_rev1 primers and 1 ng pSB1A3-*bjaR*$_{S107R}$-*gfp-kanR*(CTG) template with the following thermal cycler programme: 98 °C for 30 s, 35 cycles of [98 °C for 10 s, 63 °C for 20 s and 72 °C for 30 s], 72 °C for 5 mins, 4 °C HOLD. Two fragment homologous recombination was performed as described above.

0.3.9 pSB1A3-*bjaR-gfp* (B0034 | ACG) assembly

pSB1A3-*bjaR-gfp* (B0034 | ACG) was prepared by transferring the *bjaR* gene containing an ACG start codon from the *kanR*-containing reporter plasmid backbone used in directed evolution workflow back into the *kanR*-free backbone. pSB1A3-*bjaR-gfp* (B0034 | ACG) were formed via the recombination of two DNA strands with homologous ends, denoted pSB1A3-*gfp*-BB and *bjaR* (ACG) insert. The pSB1A3-*gfp*-BB fragment was amplified with Phusion DNA polymerase mastermix (2X) using 500 nM pSB1A3_for2 and pSB1A3_rev2 primers and 1 ng pSB1A3-*bjaR-gfp* template with the following thermal cycler programme: 98 °C for 30 s, 35 cycles of [98 °C for 10 s, 63 °C for 20 s and 72 °C for 2 mins], 72 °C for 10 mins, 4 °C HOLD. The textsubscriptS107R (ACG) insert fragment was amplified with Phusion DNA polymerase mastermix (2X) using 500 nM pSB1A3_for1 and pSB1A3_rev1 primers and 1 ng pSB1A3-*bjaR-gfp-kanR*(B0034 | ACG) template with the following thermal cycler programme: 98 °C for 30 s, 35 cycles of [98 °C for 10 s, 63 °C for 20 s and 72 °C for 30 s], 72 °C for 5 mins, 4 °C HOLD. Two fragment homologous recombination was performed as described above.

0.3.10 pSB1A3-*bjaR-gfp* (B0031/B0032/B0033 | ATG) assembly

The BBa_B0034 RBS from pSB1A3-*bjaR-gfp* was replaced by weaker BBa_B0031/32/33 RBS' via the recombination of 2 DNA strands with homologous ends, denoted pSB1A3-*bjaR-gfp* B0031/B0032/B0033 BB and B0031/B0032/B0033 insert. The pSB1A3-*bjaR-gfp* B0031/B0032/B0033 BB fragments were amplified with Phusion DNA polymerase mastermix (2X) using 500 nM pSB1A3_for1 and B0031-33_rev primers and 1 ng pSB1A3-*bjaR-gfp* template with the following thermal cycler programme: 98 °C for 30 s, 35 cycles of [98 °C for 10 s, 66 °C for 20 s and 72 °C for 1 min 15 s], 72 °C for 10 mins, 4 °C HOLD. The B0031/B0032/B0033 insert fragments were amplified with Phusion DNA polymerase mastermix (2X) using 500 nM B0031/B0032/B0033_for and pSB1A3_rev1 primers and 1 ng pSB1A3-*bjaR-gfp* template with the following thermal cycler programme: 98 °C for 30 s, 35 cycles of [98 °C for 10 s, 66 °C for 20 s and 72 °C for 30 s], 72 °C for 5 mins, 4 °C HOLD. 2 fragment homologous recombination was performed as described previously.

0.3.11 pSB1A3-*bjaR-gfp*(B0034 | CTG) assembly

The CTG start codon mutation was introduced into pSB1A3-$bjaR_{WT}$-*gfp* via the recombination of 2 DNA strands with homologous ends, denoted pSB1A3-*bjaR*(CTG)-*gfp* BB and *bjaR*(CTG) insert. pSB1A3-*bjaR*(CTG)-*gfp* BB was amplified with Phusion DNA polymerase mastermix (2X) using 500 nM pSB1A3_for2 and pSB1A3_rev2_CTG primers and 1 ng pSB1A3-textitbjaR-gfp template with the following thermal cycler programme: 98 °C for 30 s, 35 cycles of [98 °C for 10 s, 66 °C for 20 s and 72 °C for 1 min 30 s], 72 °C for 10 mins, 4 °C HOLD. *bjaR*(CTG) insert was amplified with Phusion DNA polymerase mastermix (2X) using 500 nM pSB1A3_for1_CTG and pSB1A3_rev1 and 1 ng pSB1A3-*bjaR-gfp* template with the following thermal cycler programme: 98 °C for 30 s, 35 cycles of [98 °C for 10 s, 66 °C for 20 s and 72 °C for 15 s], 72 °C for 5 mins, 4 °C HOLD. Two fragment homologous recombination was performed as described previously.

0.3.12 pSB1A3-$bjaR_{\mathrm{S107R}}$-*gfp* (B0033 | ATG) assembly

pSB1A3-$bjaR_{\mathrm{S107R}}$-*gfp* (B0033 | ATG) was prepared by introducing the S107R mutation into the *bjaR* gene within the pSB1A3-*bjaR-gfp* (B0033 | ATG) backbone. pSB1A3-$bjaR_{\mathrm{S107R}}$-*gfp* (B0033 | ATG) was formed via the recombination of two DNA strands with homologous ends, denoted $bjaR_{\mathrm{S107R}}$ (B0033) BB and $bjaR_{\mathrm{S107R}}$ insert. $bjaR_{\mathrm{S107R}}$ (B0033) BB was amplified with Phusion DNA polymerase mastermix (2X) using 500 nM pSB1A3_for2 and S107R_rev primers and 1 ng pSB1A3-*bjaR-gfp* (B0033 | ATG) template with the following thermal cycler programme: 98 °C for 30 s, 35 cycles of [98 °C for 10 s, 63 °C for 20 s and 72 °C for 1 min 30 s], 72 °C for 10 mins, 4 °C HOLD. $bjaR_{\mathrm{S107R}}$ insert was amplified with Phusion DNA polymerase mastermix (2X) using 500 nM S107R_for and pSB1A3_rev1 primers and 1 ng pSB1A3 *bjaR-gfp* (B0033 | ATG) templates with the following thermal cycler programme: 98 °C for 30 s, 35

cycles of [98 °C for 10 s, 63 °C for 20 s and 72 °C for 15 s], 72 °C for 5 mins, 4 °C HOLD. Two fragment homologous recombination was performed as described above.

**Name**	**Sequence (5'-3')**
T7_For	GAAATTAATACGACTCACTATAGGGTCTAG
T7_For_amine	GAAAT**T**AA**T**ACGAC**T**CAC**T**A**T**AGGG**T**C**T**AG
T7_For_PCB	GAAAT**T**AA**T**ACGAC**T**CAC**T**A**T**AGGG**T**C**T**AG
PURE_rev1	GATATAGTTCCTCCTTTCAG
PURE_rev2	GCCTCCTGCAGGTTAACCTTAC
PURE_for1	CTCGAGTAAGGTTAACCTGCAGGAG
*mNG*_T7g10_for1	AGATATACCATGGCTAGCATGACTGGTGGACAGCAACA TATGGTGAGCAAAGGCGAAGAG
*mNG*_T7g10_rev1	TATTTCTAGAGGGAAACCGTTGTGGTCTCCCTAGACCC TATAGTGAGTCGTATTAATTTC
*mNG*_T7g10_for2	CTCTAGAAATAATTTTGTTTAACTTTAAGAAGGAGATA TACCATGGCTAGCATGACTG
*mNG*_T7g10_rev2	GCCATGGTATATCTCCTTCTTAAAGTTAAACAAAATTA TTTCTAGAGGGAAACCGTTGTG
*mNG*_g10_for	ATGGCTAGCATGACTGGTGGAC
*mNG*_g10_rev	GTCCACCAGTCATGCTAGCCATATGTATACCTCCTTCT TAAAGTTAAAC
*mV*_T7g10_for	CATGACTGGTGGACAGCAACATATGGTGAGCAAGGGC GAGGAGC
*mV*_T7g10_rev	CATATGTTGCTGTCCACCAGTCATGC
*bjaI*_for1	GAAGGAGGTATACATATGATTCACGCAATTTCCGCGGT CAATCGCCACTTATACGAGGAC
*bjaI*_for2	GCGTCATGACATCTTTGTCGAGGAGCGGCACTGGGAG ACGCTGCGCAGGCCGGATGGCCG
*bjaI*_for3	GAGGATACCGTCTATCTGCTTGCGCTGGAGGGACGGC GCGTCGTCGGCGGCCACCGGCTC
*bjaI*_for4	CTCGATGATGAGCGAGGTCTTCCCGCATCTGGCGGCG GTTCGCGGCTGCCCCTCGGATC
*bjaI*_for5	CTACTTCGTCGTCCGCGATCGCCGCGACGGCGCGCTC AACCTGCAACTGATGGCGGCG
*bjaI*_for6	GAATCGCGCAGGTCAGCGCGATCATGGAAACCTGGTG GTTGCCGCGCTTCCACGAGGCCG
*bjaI*_for7	CTGCCGGCTCTGGTCGAGAACGCCTGGACCATGGCGG CCACCGTCGACATTCGTCGCCAG
*bjaI*_for8	GATCGCATCGGCATGCCTTCCATCGTGCAACAGGACG GCAGGACGGCCCGCGTCTGGACGCCGTCGC

*bjaI*_for9	GCCGCGCAACGAAAGAGCGCCTGATGAAATGGATCCC GGGAATTCTCGA
*bjaI*_rev1	GCTCTTTCGTTGCGCGGCAGCGAGGCCGCAGCACGGG CGACGGCGTCCAGACG
*bjaI*_rev2	GAAGGCATGCCGATGCGATCATGCAGGACATCGAGCG TCTGGCGACGAATGTCGACG
*bjaI*_rev3	CTCGACCAGAGCCGGCAGGCCGAGCGGCGTCACGACG AAGCCGGCCTCGTGGAAGCGCG
*bjaI*_rev4	GCTGACCTGCGCGATTCCCTGGTCGAGGCAGAACTCC TGCACCGCCGCCATCAGTTGCAG
*bjaI*_rev5	GATCGCGGACGACGAAGTAGCGCGACCATTCCCAGAT CAGCGGATCCGAGGGGCAGCCGC
*bjaI*_rev6	GACCTCGCTCATCATCGAGGGCTTGGTCGTGGGGTAG AGCCGGTGGCCGCCGAC
*bjaI*_rev7	GCAGATAGACGGTATCCTCGTCGTCATAGGAATCGACC TCGCGGCCATCCGGCCTGCGC
*bjaI*_rev8	GACAAAGATGTCATGACGCAGCCGGAAATGCTGCTCG AGTACGTCCTCGTATAAGTGGCG
*bjaI*_for_amp	GAAGGAGGTATACATATGATTCACGC
*bjaI*_rev_amp	CGAGAATTCCCGGGATCCATTTC
*bjaR*_KO_For	TGACATTCTCTCAAAGTATTATGCAGGGCCATCCGTC ACAAAATTATCAAC
*bjaR*_KO_Rev	CCTGCATAATACTTTGAGAGAATGTCACAGGGCTTCA CGCCCATAATCTAC
*kanR*_for1	AGTAAATCTAAGCAGGTCCGCATGAGCCATATTCAACG GGAAAC
*kanR*_for2	CATGGCATGGATGAACTATACAAATAATAAAGTAAATC TAAGCAGGTCCGC
*kanR*_rev	CTGACCGATAAGCCGGCTCTAGTATTATTAG AAAAACTCATCGAGCATC
pSB1A3_for1	GCTACTAGAGAAAGAGGAGAAATACTAG
pSB1A3_for2	TACTAGAGCCAGGCATCAAATAAAACG
pSB1A3_for3	TACTAGAGCCGGCTTATCGGTCAG
pSB1A3_rev1	CGTTTTATTTGATGCCTGGCTCTAGTA
pSB1A3_rev2	CTAGTATTTCTCCTCTTTCTCTAGTAGC
pSB1A3_rev3	TTATTATTTGTATAGTTCATCCATGCCATG
pSB1A3_for1_CTG	GCTACTAGAGAAAGAGGAGAAATACTAGCTG
pSB1A3_rev2_CTG	CAGCTAGTATTTCTCCTCTTTCTCTAGTAGC
B0030_for	CTAGAGATTAAAGAGGAGAAATACTAGATGTCCGCAGT AGATTATGGGC

B0030_rev	GTATTTCTCCTCTTTAATCTCTAGTAGCTAGCACTGTA CCTAGGAC
B0031_for	CTAGAGTCACACAGGAAACCTACTAGATGTCCGCAGTA GATTATGGGC
B0031_rev	GTAGGTTTCCTGTGTGACTCTAGTAGCTAGCACTGTAC CTAGGAC
B0032_for	CTAGAGTCACACAGGAAAGTACTAGATGTCCGCAGTAG ATTATGGGC
B0032_rev	CTAGTACTTTCCTGTGTGACTCTAGTAGCTAGCACTGT ACCTAGGAC
B0033_for	CTACTAGAGTCACACAGGACTACTAGATGTCCGCAGTA GATTATGGGC
B0033_rev	GTAGTCCTGTGTGACTCTAGTAGCTAGCACTGTACCTA GGAC
S107R_for	GAATTAGAACCACGCGCCGC
S107R_rev	GCGGCGCGTGGTTCTAATTC

Table 2: Primer sequences
Bold bases indicate an amino-C6-thymine modified base. Bold and underlined bases indicate a PCB-modification from the amino-C6-thymine base.

Bibliography

1. Booth, M. J., Schild, V. R., Graham, A. D., Olof, S. N. & Bayley, H. Light-activated communication in synthetic tissues. *Sci Adv* **2,** e1600056. ISSN: 2375-2548 (Electronic) 2375-2548 (Linking). https://www.ncbi.nlm.nih.gov/pubmed/27051884 (2016).
2. Jacobus, A. P. & Gross, J. Optimal cloning of PCR fragments by homologous recombination in Escherichia coli. *PLoS One* **10,** e0119221. ISSN: 1932-6203 (Electronic) 1932-6203 (Linking). https://www.ncbi.nlm.nih.gov/pubmed/25774528 (2015).
3. Venancio-Marques, A. *et al.* Modification-free photocontrol of beta-lactam conversion with spatiotemporal resolution. *ACS Synth Biol* **1,** 526–31. ISSN: 2161-5063 (Electronic) 2161-5063 (Linking). https://www.ncbi.nlm.nih.gov/pubmed/23656229 (2012).
4. Lindemann, A. *et al.* Isovaleryl-homoserine lactone, an unusual branched-chain quorum-sensing signal from the soybean symbiont Bradyrhizobium japonicum. *Proc Natl Acad Sci U S A* **108,** 16765–70. ISSN: 1091-6490 (Electronic) 0027-8424 (Linking). https://www.ncbi.nlm.nih.gov/pubmed/21949379 (2011).
5. Stemmer, W. P., Crameri, A., Ha, K. D., Brennan, T. M. & Heyneker, H. L. Single-step assembly of a gene and entire plasmid from large numbers of oligodeoxyribonucleotides. *Gene* **164,** 49–53. ISSN: 0378-1119 (Print) 0378-1119 (Linking). https://www.ncbi.nlm.nih.gov/pubmed/7590320 (1995).
