# Supplementary material for: Engineering cellular communication between light-activated synthetic cells and bacteria
Source: Nat Chem Biol. 2023 Jul 6;19(9):1138–46. doi: 10.1038/s41589-023-01374-7 (PMC10449621; doi:10.1038/s41589-023-01374-7)
Supplement: Supplementary file 2 — Reporting Summary [file 41589_2023_1374_MOESM2_ESM.pdf]

nature portfolio

Corresponding author(s): Michael Booth

Last updated by author(s): May 2, 2023

Reporting Summary

Nature Portfolio wishes to improve the reproducibility of the work that we publish. This form provides structure for consistency and transparency in reporting. For further information on Nature Portfolio policies, see our Editorial Policies and the Editorial Policy Checklist.

Statistics

For all statistical analyses, confirm that the following items are present in the figure legend, table legend, main text, or Methods section.

n/a	Confirmed	
☐	☒	The exact sample size (n) for each experimental group/condition, given as a discrete number and unit of measurement
☐	☒	A statement on whether measurements were taken from distinct samples or whether the same sample was measured repeatedly
☒	☐	The statistical test(s) used AND whether they are one- or two-sided<br>*Only common tests should be described solely by name; describe more complex techniques in the Methods section.*
☒	☐	A description of all covariates tested
☒	☐	A description of any assumptions or corrections, such as tests of normality and adjustment for multiple comparisons
☐	☒	A full description of the statistical parameters including central tendency (e.g. means) or other basic estimates (e.g. regression coefficient) AND variation (e.g. standard deviation) or associated estimates of uncertainty (e.g. confidence intervals)
☒	☐	For null hypothesis testing, the test statistic (e.g. F, t, r) with confidence intervals, effect sizes, degrees of freedom and P value noted<br>*Give P values as exact values whenever suitable.*
☒	☐	For Bayesian analysis, information on the choice of priors and Markov chain Monte Carlo settings
☒	☐	For hierarchical and complex designs, identification of the appropriate level for tests and full reporting of outcomes
☒	☐	Estimates of effect sizes (e.g. Cohen's d, Pearson's r), indicating how they were calculated

Our web collection on statistics for biologists contains articles on many of the points above.

Software and code

Policy information about availability of computer code

Data collection	Fluorescence spectroscopy data were collected with i-Control software (Version 1.11.1.0) using a Tecan Infinite M1000 fluorescence plate reader. Fluorescence microscopy images were collected in Leica Application Suite X (Version 3.0.0.15697) using a Leica DMI8 epifluorescence microscope, equipped with a Leica DFC7000T camera and Leica EL6000 light source. Agarose gel images were obtained using the transilluminator on a Biorad Gel Doc XR.
Data analysis	Bulk CFPS and E. coli fluorescence intensity data were analysed and plotted using Matlab (Version R2020a). Dose-response curves were fit to a hill function using the nlinfit function in Matlab. Fluorescence microscopy figures were prepared using ImageJ (Version 1.52i). Post brightness/ contrast normalisation using imageJ, a custom MatLab script was used to analyse fluorescence intensity of the vesicles produced in this study. This is available at https://zenodo.org/record/7729425.

For manuscripts utilizing custom algorithms or software that are central to the research but not yet described in published literature, software must be made available to editors and reviewers. We strongly encourage code deposition in a community repository (e.g. GitHub). See the Nature Portfolio guidelines for submitting code & software for further information.

Data

Policy information about availability of data

All manuscripts must include a data availability statement. This statement should provide the following information, where applicable:

- Accession codes, unique identifiers, or web links for publicly available datasets
- A description of any restrictions on data availability
- For clinical datasets or third party data, please ensure that the statement adheres to our policy

Source data for figures 1,3, and 4 can be found within the article. Source data for supplementary figures 2, 3, 4, 6, 7, 8, 9, 11, 12, 13, 14 can be found at https://zenodo.org/record/7808487. The bjaI gene sequence was obtained form the European Nucleotide Archive database (Accession: BA000040.2; ORF_ID: blr1063).

Human research participants

Policy information about studies involving human research participants and Sex and Gender in Research.

Reporting on sex and gender	N/A
Population characteristics	N/A
Recruitment	N/A
Ethics oversight	N/A

Note that full information on the approval of the study protocol must also be provided in the manuscript.

Field-specific reporting

Please select the one below that is the best fit for your research. If you are not sure, read the appropriate sections before making your selection.

☒ Life sciences ☐ Behavioural & social sciences ☐ Ecological, evolutionary & environmental sciences

For a reference copy of the document with all sections, see nature.com/documents/nr-reporting-summary-flat.pdf

Life sciences study design

All studies must disclose on these points even when the disclosure is negative.

Sample size	No statistical methods were used to predetermine sample size. Sample size was determined to be sufficient when a statistical comparision could be made to control samples (typically 3). Sample sizes for each experiment are given in the figure legends. 45 mutants were included in the directed evolution screen as this was the upper limit that could be tested against both -/+ IV-HSL conditions, within a single 96 well plate (allowing for controls).
Data exclusions	No data was excluded.
Replication	n=3 replication was performed for most experiments, with the exact number given for each experiment in figure legends, as is consistent with life science research. All attempts at replication were successful.
Randomization	Randomisation was not relevant to this work. All samples were treated identically for each experiment.
Blinding	Blinding was not relevant to this work

Reporting for specific materials, systems and methods

We require information from authors about some types of materials, experimental systems and methods used in many studies. Here, indicate whether each material, system or method listed is relevant to your study. If you are not sure if a list item applies to your research, read the appropriate section before selecting a response.

Materials & experimental systems

n/a	Involved in the study
☒	☐ Antibodies
☒	☐ Eukaryotic cell lines
☒	☐ Palaeontology and archaeology
☒	☐ Animals and other organisms
☒	☐ Clinical data
☒	☐ Dual use research of concern

Methods

n/a	Involved in the study
☒	☐ ChIP-seq
☒	☐ Flow cytometry
☒	☐ MRI-based neuroimaging
