# Supplementary figures and images for: Engineering cellular communication between light-activated synthetic cells and bacteria
Source: Nat Chem Biol. 2023 Jul 6;19(9):1138–46. doi: 10.1038/s41589-023-01374-7 (PMC10449621; doi:10.1038/s41589-023-01374-7)

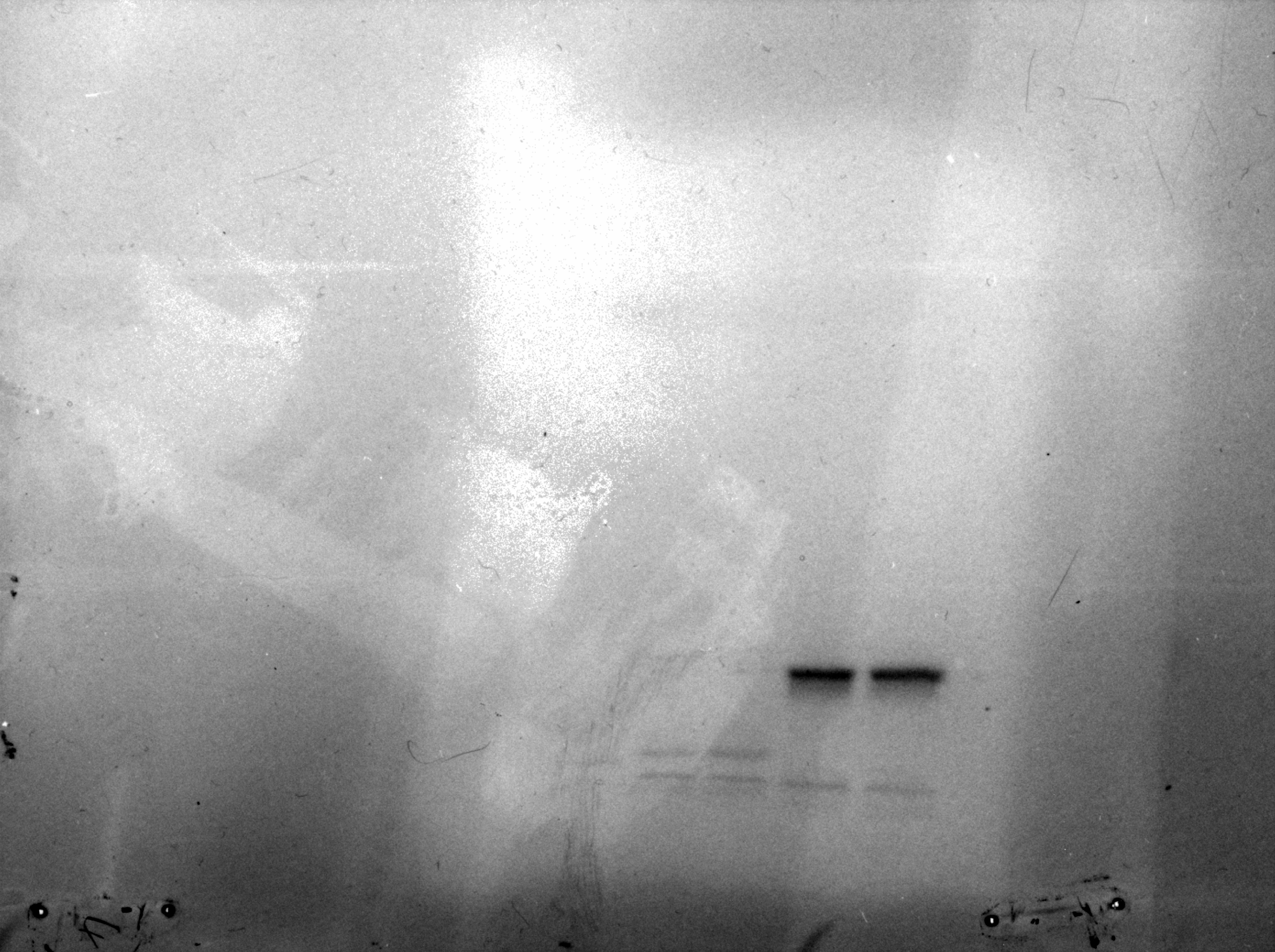

Supplement: Source Data Fig. 4 — Numerical data representing the OD600, fluorescence intensity and calculated OD-normalized fluorescence intensity for BjaR receiver cell dose–response curves using biosynthetic IV-HSL containing samples. Data for each subfigure are given on a separate sheet. An uncropped image of the autoradiograph indicating the location of the bjaI produced by CFPS. An uncropped image of the Coomassie-stained SDS–PAGE gel of the bjaI CFPS reactions. [file 41589_2023_1374_MOESM5_ESM.zip › Figure4b_autoradiograph.tif]

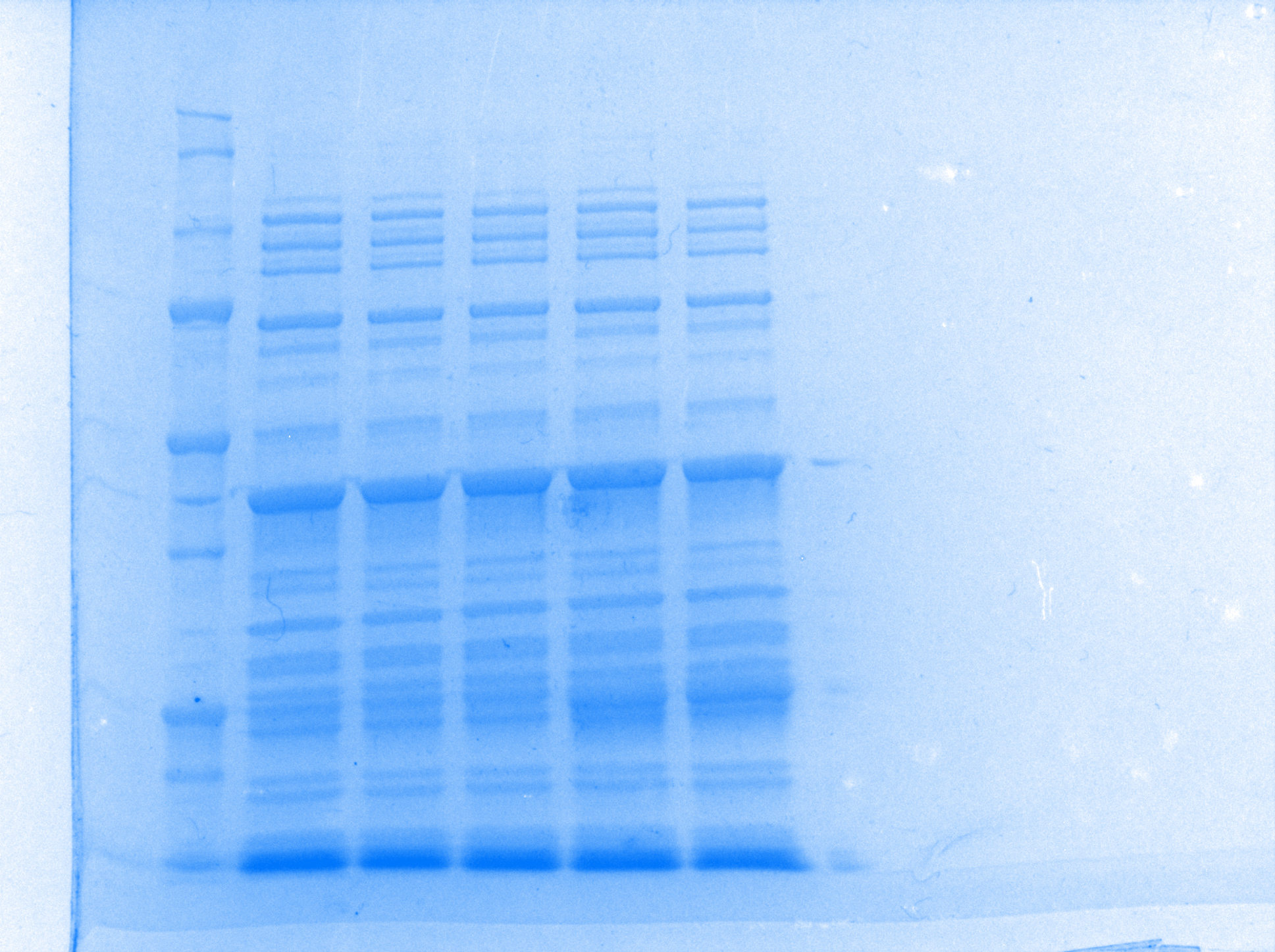

Supplement: Source Data Fig. 4 — Numerical data representing the OD600, fluorescence intensity and calculated OD-normalized fluorescence intensity for BjaR receiver cell dose–response curves using biosynthetic IV-HSL containing samples. Data for each subfigure are given on a separate sheet. An uncropped image of the autoradiograph indicating the location of the bjaI produced by CFPS. An uncropped image of the Coomassie-stained SDS–PAGE gel of the bjaI CFPS reactions. [file 41589_2023_1374_MOESM5_ESM.zip › Figure4b_coomassie.tif]
